# Management of partial-thickness rotator cuff tears with autologous adipose-derived regenerative cells is safe and more effective than injection of corticosteroid

**DOI:** 10.1038/s41598-023-46653-4

**Published:** 2023-11-07

**Authors:** Mark Lundeen, Jason L. Hurd, Matthew Hayes, Meredith Hayes, Tiffany R. Facile, John P. Furia, Nicola Maffulli, Christopher Alt, Eckhard U. Alt, Christoph Schmitz, David A. Pearce

**Affiliations:** 1 Sanford Orthopedics and Sports Medicine Fargo, Fargo, ND USA; 2 Sanford Orthopedics and Sports Medicine Sioux Falls, Sioux Falls, SD USA; 3 Sanford Radiology Clinic, Sioux Falls, SD USA; 4 Sanford Health, Sioux Falls, SD USA; 5 SUN Orthopedics of Evangelical Community Hospital, Lewisburg, PA USA; 6 Department of Trauma and Orthopaedic Surgery, Sapienza University of Rome, Sant’Andrea Hospital, Rome, Italy; 7 Centre for Sports and Exercise Medicine, Barts and The London School of Medicine and Dentistry, Mile End Hospital, Queen Mary University of London, London, UK; 8 School of Pharmacy and Bioengineering, Guy Hilton Research Centre, Keele University School of Medicine, Stoke on Trent, UK; 9 InGeneron, Inc., Houston, TX USA; 10 Institute of Anatomy, Faculty of Medicine, LMU Munich, Munich, Germany; 11 Isar Klinikum, Munich, Germany; 12 Sanford School of Medicine, University of South Dakota, Sioux Falls, SD USA; 13 Heart and Vascular Institute, Department of Medicine, Tulane University Health Science Center, New Orleans, LA USA; 14 Sanford Research, Sioux Falls, SD USA

**Keywords:** Health care, Stem-cell therapies

## Abstract

Symptomatic, partial-thickness rotator cuff tears (sPTRCT) are problematic. This study tested the hypothesis that management of sPTRCT with injection of fresh, uncultured, unmodified, autologous, adipose-derived regenerative cells (UA-ADRCs) is safe and more effective than injection of corticosteroid even in the long run. To this end, subjects who had completed a former randomized controlled trial were enrolled in the present study. At baseline these subjects had not responded to physical therapy treatments for at least 6 weeks, and were randomly assigned to receive respectively a single injection of UA-ADRCs (n = 11) or a single injection of methylprednisolone (n = 5). Efficacy was assessed using the ASES Total score, pain visual analogue scale (VAS), RAND Short Form-36 Health Survey and range of motion at 33.2 ± 1.0 (mean ± SD) and 40.6 ± 1.9 months post-treatment. Proton density, fat-saturated, T2-weighted MRI of the index shoulder was performed at both study visits. There were no greater risks connected with injection of UA-ADRCs than those connected with injection of corticosteroid. The subjects in the UA-ADRCs group showed statistically significantly higher mean ASES Total scores than the subjects in the corticosteroid group. The MRI scans at 6 months post-treatment allowed to “watch the UA-ADRCs at work”.

## Introduction

Symptomatic, partial-thickness rotator cuff tear (sPTRCT) is a common cause of shoulder pain, loss of function and occupational disability^1–3^. Cadaveric and magnetic resonance imaging (MRI) studies reported the incidence of partial-thickness rotator cuff tears between 13 and 25%, with an increasing incidence with age^4–6^. The majority of sPTRCT cases are associated with aging, repetitive overhead use of the arm, sudden and forceful trauma, or a combination of these factors^1–3^.

Current non-surgical and surgical treatment options to address sPTRCT do not offer the potential to naturally replace damaged tendon tissue and often do not improve clinical results. Subacromial injection of corticosteroids, among the most widely used nonoperative treatment options for sPTRCT^7^, can provide short-term pain relief but may not modify the course of the condition^7^. Even worse, subacromial injection of corticosteroid carries the risk that a partial-thickness rotator cuff tear develops into a full-thickness rotator cuff tear^8^. A recent meta-analysis and a recent double-blinded, randomized controlled clinical trial (RCT) concluded that injections of platelet rich plasma might also not be beneficial in non-operative treatment of rotator cuff disease^9,10^. Surgical treatment of sPTRCT is generally successful among patients who, for a period of 3–6 months, unsuccessfully underwent conservative treatment modalities^2^. However, surgical intervention presents potential complications and a more lengthy recovery, and some authors have argued that results from these procedures may not exceed those obtained with conservative management^11^.

A recent, first-in-human RCT^12^ (hereafter: the former study) indicated that management of sPTRCT with fresh, uncultured, unmodified, autologous, adipose-derived regenerative cells (UA-ADRCs) isolated from lipoaspirate at the point of care is safe and leads to improved shoulder function without adverse effects. This study also showed that the risks associated with treating sPTRCT with UA-ADRCs were as low as those associated with injection of corticosteroid over 12 months post-treatment, with no serious adverse events observed for either treatment^12^.

Unlike most other cell preparations currently under investigation for use in regenerative medicine (including cultured adipose derived stem cells (ADSCs), induced pluripotent stem cells, etc.) UA-ADRCs are not expanded in culture, and are therefore not exposed to potential, culture-related mechanic and oxidative stress that could affect their safety as a medicinal product^13^. Furthermore, UA-ADRCs do not share the risk of potentially developing tumors (reported for induced pluripotent stem cells) and immunological defensive reactions (reported for allogeneic adult stem cells)^14,15^. Only 0.001–0.1% of the total population of bone marrow nucleated cells represent mesenchymal stromal cells, whereas these cells can represent up to 12% of the total population of UA-ADRCs^16,17^. Additionally, harvesting adipose tissue is typically much less invasive than harvesting bone marrow^18^.

In the former study^12^ subjects were not followed up beyond 12 months post-treatment. The present study tested the hypothesis that management of sPTRCT with injection of UA-ADRCs is safe and more effective than injection of corticosteroid even in the long run, with a minimum follow-up of 36 months.

## Methods

### Study design

The present study was a long term follow-up study of a first-in-human, two center, prospective, open-label, randomized controlled trial^12^. Both the present and the former studies^12^ were conducted at Sanford Orthopedics and Sports Medicine—Fargo (Fargo, ND, USA; principal investigator (PI): M.L.) and Sanford Orthopedics and Sports Medicine—Sioux Falls (Sioux Falls, SD, USA; PI: J.H.).

**Figure 1:**
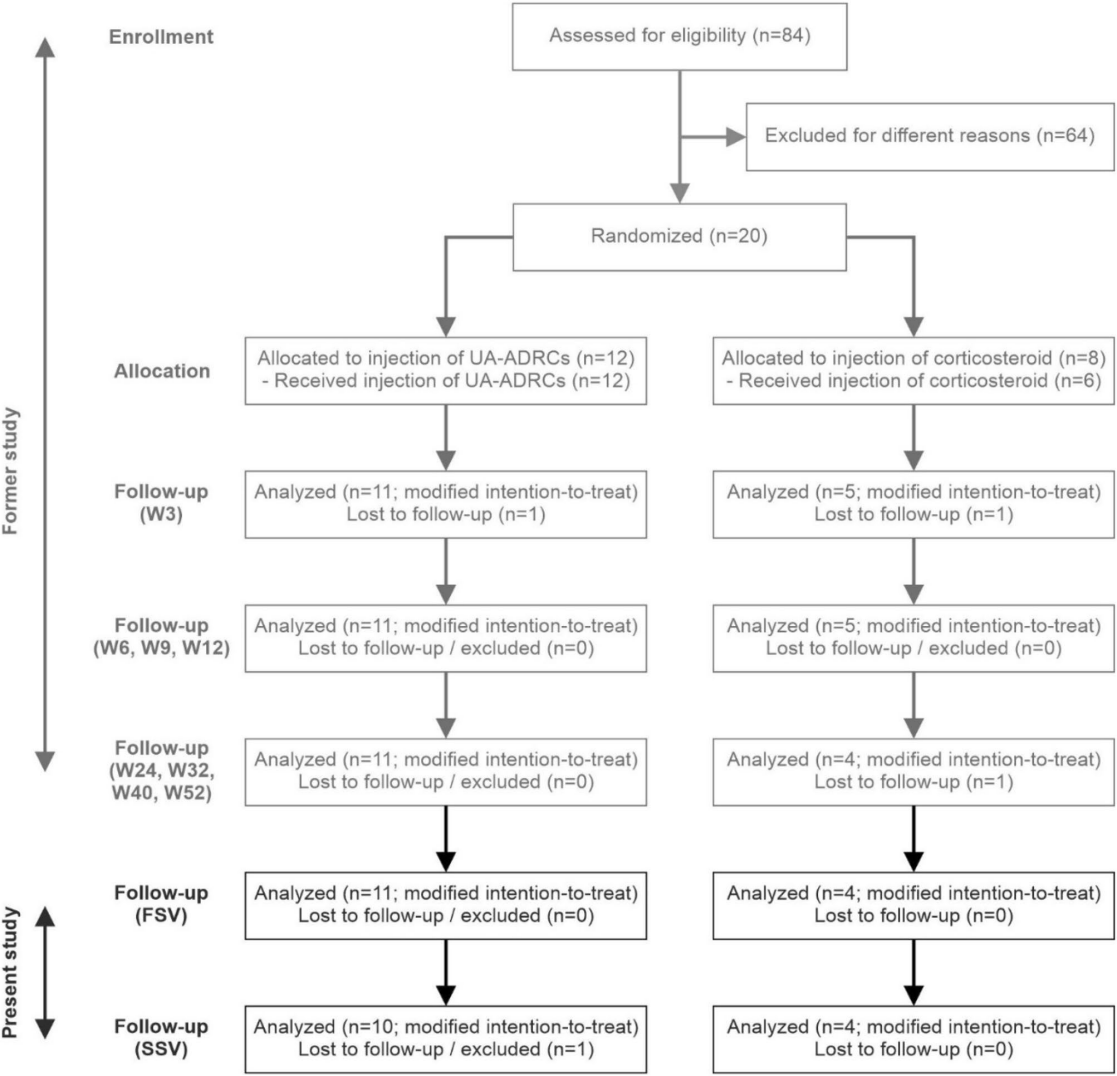
Flow of subjects in the present and the former studies^12^ according to CONSORT^19^. *W3*/*W6*/*W9*/*W12*/*W24*/*W32*/*W40*/*W52* study visits scheduled in the former study^12^ at 3/6/9/12/24/32/40/52 weeks post-treatment, *FSV* first study visit of the present study at 33.2 ± 1.0 (mean ± standard deviation) months post-treatment, *SSV* second study visit of the present study at 40.6 ± 1.9 months post-treatment.

Figure 1 shows the flow of subjects in the present and the former studies^12^ according to the CONSORT statement^19^.

### Ethics

The present study received Investigational Device Exemption (IDE) from the U.S. Food and Drug Administration (FDA) on 13/05/2019 (no. 16956), received Institutional Review Board (IRB) approval from WIRB Copernicus Group, Inc. (Olympia, WA, USA) on 23/07/2019 for study site Sanford USD Medical Center, Sioux Falls, SD, USA (IRB Tracking Number: 20191931; Study Number: 1263181) and on 29/09/2019 for study site Sanford Orthopedic Sports Medicine Clinic, Fargo, ND, USA (IRB Tracking Number: 20191931; Study Number: 1266705), and was registered at Clinicaltrials.gov on 04/09/2019 (NCT04077190). The first subject was enrolled in the present study on 21/11/2019, and the last subject on 20/03/2020. The present study was closed on 05/05/2022 at study site Sanford Orthopedic Sports Medicine Clinic, Fargo, ND, USA, and on 09/05/2022 at study site Sanford USD Medical Center, Sioux Falls, SD, USA.

Written, informed consent to participate was obtained from all subjects.

The former study^12^ received IDE from the FDA on 23/09/2016 (no. 16956), was registered at Clinicaltrials.gov on 28/09/2016 (ID NCT02918136), and received IRB approval of Sanford Health (Sioux Falls, SD, USA) on 04/11/2016 (Sanford IRB #3 registration number 00007985) in accordance with the Declaration of Helsinki. The first subject was enrolled in the former study^12^ on 04/01/2017, and the last subject on 21/04/2017. The study was closed on 07/11/2019. After having received additional IRB approval from Sanford Health on 07/11/2019 (Sanford IRB #3 registration number STUDY00001869) to re-examine MRI scans, the former study^12^ was re-opened on 14/09/2020.

### Participants, randomization and interventions

In brief, all the subjects enrolled in the former study^12^ suffered from a sPTRCT of the supraspinatus tendon at baseline, had not responded to physical therapy treatments for at least 6 weeks, and were randomly assigned to receive either a single injection of an average 11.4 × 10^6^ UA-ADRCs (in 5 mL liquid; mean cell viability: 88%) (n = 11; modified intention-to-treat (mITT) population) (hereafter: UA-ADRCs group) or a single injection of 80 mg of methylprednisolone (40 mg/mL; 2 mL) plus 3 mL of 0.25% bupivacaine (n = 5 in the former study^12^; n = 4 in the present study; mITT population) (hereafter: corticosteroid group). The UA-ADRCs were isolated from lipoaspirate using the Transpose RT system (InGeneron, Houston, TX, USA)^15,20^. The process of isolating UA-ADRCs from lipoaspirate is shown in Supplementary Figs. S1 and S2 online. One subject in the corticosteroid group experienced progression of sPTRCT into a symptomatic, massive full-thickness rotator cuff tear during the former study^12^ and was therefore not enrolled in the present study. For this reason, the baseline data of the subjects in the corticosteroid group enrolled in the present study (summarized in Table 1) slightly differ from the baseline data of those in the corticosteroid group provided in the former study^12^.

**Table 1:** Characteristics of the subjects enrolled in the present study at baseline (modified intention-to-treat population).

Variable	UA-ADRCs group (n = 11)	Corticosteroid group (n = 4)
Age, years, median; mean (SD; min; max)	64.6; 62.3 (9.6; 40; 74)	59.0; 56.8 (7.6; 46; 63)
Woman, n (%)	3 (27.3)	0 (0)
Body weight, kg, median; mean (SD; min; max)	93.9; 88.6 (18.1; 51.6; 111.1)	113.9; 109.9 (25.7; 74.5; 133.7)
Body height, cm, median; mean (SD; min; max)	178; 176 (8.8; 157; 188)	178; 178 (4.8; 173; 185)
Body mass index, kg/m^2^, median; mean (SD; min; max)	29.9; 28.4 (4.1; 20.8; 33.3)	35.7; 34.3 (7.9; 23.4; 42.3)
Affected shoulder, right (%)	9 (81.8)	2 (50.0)
ASES Total score, median; mean (SD; min; max)	56.7; 58.7 (19.2; 30; 92)	46.7; 47.1 (14.6; 30; 65)
SF-36 Total score, median; mean (SD; min; max)	604; 557 (134; 270; 695)	565; 547 (83; 432; 627)
VAS Pain score, median; mean (SD; min; max)	5; 4.7 (2.8; 0; 8)	6; 5.8 (2.1; 3; 8)
ROM—FE_A, degrees, median; mean (SD; min; max)	160.0, 163.4 (10.3; 154; 180)	154.4, 156.0 (6.4; 150; 165)
ROM—FE_P, degrees, median; mean (SD; min; max)	173.9, 174.1 (4.7; 165; 180)	173.9, 172.9 (1.9; 170; 174)
ROM—ER_A, degrees, median; mean (SD; min; max)	68.3, 70.6 (8.6; 50; 80)	68.3, 61.7 (18.1; 35; 75)
ROM—ER_P, degrees, median; mean (SD; min; max)	75.0, 73.2 (9.0; 50;80)	70.0, 61.3 (17.5; 35; 70)
ROM—ER_90_A, degrees, median; mean (SD; min; max)	75.0, 79.6 (7.2; 70; 90)	72.5, 66.3 (14.4; 45; 75)
ROM_ER_90_P, degrees, median; mean (SD; min; max)	82.5; 83.9 (4.1; 80; 90)	82.5; 79.4 (6.3; 70; 83)
Tear volume, mm^3^, median; mean (SD; min; max)	47.3; 58.6 (37.4; 19.8; 128.9)	27.0; 25.1 (8.3; 14.6; 31.7)

*SD* standard deviation, *min* minimum value, *max* maximum value, *VAS* visual analog scale, *ASES* American Shoulder and Elbow Surgeons Standardized Shoulder Assessment Form^21^, *SF-36* RAND Short Form-36 Health Survey^22^, *VAS* visual analogue scale; *ROM*, range of motion; *FE_A*, active forward elevation; *FE_P*, passive forward elevation; *ER_A*, active external rotation with the index arm comfortably at side; *ER_P*, passive external rotation with the index arm comfortably at side; *ER_90_A*, active external rotation with the index arm at 90° abduction; *ER_90_P*, passive external rotation with the index arm at 90° abduction.

### Outcome measurements and assessments

According to the study protocol the primary endpoints of the present study were long-term safety as indicated through the rate of treatment emergent adverse events (TEAEs), and long-term efficacy of pain and function through American Shoulder and Elbow Surgeons Standardized Shoulder Assessment Form^21^ (ASES Total score) and RAND Short Form-36^22^ (SF-36) health questionnaires between the UA-ADRCs group and the corticosteroid group. The secondary endpoint of the present study was long-term efficacy evaluated through VAS Pain score and MRI pre- and post-injection for the therapeutic intent to treat sPTRCT between the UA-ADRCs group and the corticosteroid group. Active and passive range of motion (ROM) of the index arm (forward elevation, external rotation with the index arm comfortably at side and external rotation with the index arm at 90° abduction) were additional endpoints.

Adverse events in the present and the former studies^12^ were defined as any untoward or unfavorable medical occurrence in a subject, including any abnormal sign, symptom or disease temporally associated with the subject’s participation in these studies, whether or not considered related to the subject’s participation in these studies.

In the former study^12^, safety was assessed immediately after treatment and 3 weeks (W3), W6, W9, W12, W24, W32, W40 and W52 post-treatment; ASES Total score, SF-36 Total score, VAS Pain score, and active and passive ROM were assessed at baseline (BL) and at W3, W6, W9, W12, W24, W32, W40 and W52 post-treatment; MRI was performed at BL and at W24 and W52 post-treatment.

In the present study, the aforementioned endpoints were assessed at 33.2 ± 1.0 (mean ± standard deviation) months post-treatment (range, 30.7–34.7) (first study visit; FSV) and at 40.6 ± 1.9 months post-treatment (range, 36.5–44.7) (second study visit; SSV).

### Analysis of MRI scans

Next to the determination of the partial-thickness tear size (calculated as ellipsoid volume), the proton density weighted, fat saturated, T2-weighted (PD FS T2) coronal MRI scans of all subjects who were enrolled in the present study were transferred in digital and fully anonymized form (compliant with the HIPAA regulation)^23^ to C.S. who was only aware of the Subject IDs. Then, C.S. mounted these MRI scans as shown in Supplementary Figs. S3–S17 online, evaluated them and indicated hyperintense structures at the position of the supraspinatus tendon that were present at W24 post-treatment but not at baseline (arrows in Supplementary Figs. S3–S9 and S11–S13 online). Afterwards, the files with the MRI scan montages and the indicated hyperintense structures were transferred in fully anonymized form (even without the Subject IDs) to M.H. and M.H., who performed an independent, blinded re-analysis of the hyperintense structures at the position of the supraspinatus tendon indicated by C.S.

### Estimand strategies for handling intercurrent events

In line with the new *The International Council for Harmonisation of Technical Requirements for Pharmaceuticals for Human Use (ICH) E9 (R1) Addendum* on the use of estimands in clinical trials (i.e., a precise description of the treatment effect to be estimated from a trial (the question)^24–26^) a comprehensive estimand was constructed for the present study. The four components of this estimand (Population (i.e., the target population for the research question), Variables (i.e., the endpoints that were obtained from all subjects), Intercurrent Events (i.e., all events that occurred after treatment initiation and either precluded the observation of a variable, or affected its interpretation) and Population-Level Summary (i.e., the variables on which the comparison between treatments was based) are outlined in Supplementary Part 9 online.

In short, in case of treatment failures (comprising all intercurrent events that required additional injections of corticosteroid into the index shoulder or surgery of the index shoulder that were definitely, probably or possibly related to the study treatments, including development of a full-thickness rotator cuff tear) subjects' data were handled using a combination of the While-on-Treatment Strategy^24–26^ and the Composite Strategy^24–26^. Specifically, response to study treatment before the occurrence of the intercurrent event was handled using the While-on-Treatment Strategy (i.e., subjects' data were used as collected), whereas response to study treatment after the occurrence of the intercurrent event was imputed according to the Composite Variable Strategy as minimum ASES Total score (0), minimum SF-36 Total score (0), maximum VAS pain score (10), minimum active and passive ROM (0) and maximum tear volume measured on MRIs (150 mm^3^, which was greater than all data measured during the present and the former studies^12^).

In contrast, intercurrent events that required additional injections of corticosteroid into the index shoulder or surgery of the index shoulder that were unlikely related or unrelated to the study treatments (e.g., accidents that affected the index shoulder) were handled using a combination of the While-on-Treatment Strategy and a Hyopthetical Strategy^24–26^. Specifically, response to the study treatment before the occurrence of the intercurrent event was handled according to the While-on-Treatment Strategy, whereas response to the study treatment after the occurrence of the intercurrent event was imputed according to a Hypothetical Strategy in which the intercurrent event would not occur. Imputation of subjects' data after occurrence of the intercurrent event was performed using the Last Observation Carried Forward approach^27,28^.

In line with recommendations in the literature^29,30^, the standard of care of symptomatic, full-thickness rotator cuff tears at our clinics is surgery. This was the reason why subjects in the corticosteroid group who experienced progression of sPTRCT into a symptomatic, full-thickness rotator cuff tear during the present and the former studies^12^ were not offered to cross over to the UA-ADRCs group. The latter was equally not offered to subjects in the corticosteroid group who developed increased pain of the index shoulder at any time post-treatment during the present and the former studies^12^ because of their nature as first-in-human studies with respect to the management of sPTRCT with injection of UA-ADRCs.

### Statistical analysis

Statistical analysis of the safety data included group-specific comparisons of the following variables: (i) total number of TEAEs, (ii) number of TEAEs experienced per subject, (iii) number of TEAEs classified as {mild/moderate/severe}, (iv) relationship of TEAEs to treatment classified as {not related/unlikely/possible/probable/definite} and (v) number of TEAEs classified as {mild and unlikely to be related to the investigated treatment/mild and possibly related to the investigated treatment/moderate and unlikely to be related to the investigated treatment/moderate and possibly related to the investigated treatment}. According to the protocol of the present study these comparisons were performed for the following time periods: from BL to W24 post-treatment (considering only data of the former study^12^), from BL to FSV in the present study, and from BL to SSV in the present study (each considering data of the present and the former studies^12^). Comparisons were performed using Chi-square test or Chi-square test for trend, respectively.

Statistical analysis of the efficacy data included calculation of the group specific mean, standard error of the mean and median as well as group-specific comparisons of the following variables: (i) ASES Total score, (ii) SF-36 Total score, (iii) VAS pain score, (iv) active and passive ROM and (v) tear volume measured on MRIs. According to the protocol of the present study these comparisons were performed at BL and at W24 and W52 post-treatment (data of the former study^12^) as well as at FSV and SSV (data of the present study). Given the non-parametric nature of all efficacy data after imputation according to the estimand, comparisons were performed using the Mann–Whitney test.

In all analyses, a p value < 0.05 was considered statistically significant. Calculations were performed using GraphPad Prism (Version 9.4.1 for Windows; GraphPad Software, San Diego, CA, USA).

## Results

### Long-term safety of treating sPTRCT with injection of either UA-ADRCs or corticosteroid

The subjects in the UA-ADRCs group reported a total number of 58 TEAEs (35 TEAEs during the former study^12^ and 23 TEAEs during the present study) (details in Supplementary Table S1 online). The subjects in the corticosteroid group reported a total number of 25 TEAEs (12 TEAEs during the former study^12^ and 13 TEAEs during the present study) (details in Supplementary Table S2 online).

No TEAE that occurred during the present and the former studies^12^ was classified as probably or definitely related to the investigated treatment. Furthermore, all severe TEAEs that occurred during the present and the former studies^12^ were classified as not related to the investigated treatment.

All subjects reported experiencing at least one TEAE. The number of subjects who experienced 1/2/3/4/6/7/8/10/12 TEAEs in the present and the former studies^12^ was 1/0/2/3/1/1/2/1/0 in the UA-ADRCs group (5.3 ± 2.7 (mean ± SEM); median, 4) and 0/1/1/2/0/0/0/0/1 in the corticosteroid group (5.0 ± 1.8; median, 4). These data were not significantly different between the groups (Chi-square test for trend; p = 0.778) (details in Supplementary Table S3 online).

The number of TEAEs classified as {mild/moderate/severe} in the present and the former studies^12^ was 38/16/4 in the UA-ADRCs group and 17/8/0 in the corticosteroid group. These data were not significantly different between the groups (Chi-square test for trend; p = 0.497) (details in Supplementary Tables S4–S6 online).

The relationship of TEAEs to treatment classified as {not related/unlikely/possible/probable/definite} in the present and the former studies^12^ was 48/6/4/0/0 in the UA-ADRCs group and 20/3/2/0/0 in the corticosteroid group. These data were not significantly different between the groups (Chi-square test; p = 0.956) (details in Supplementary Tables S7–S9 online).

The number of TEAEs classified as {mild and unlikely to be related to the investigated treatment/mild and possibly related to the investigated treatment/moderate and unlikely to be related to the investigated treatment/moderate and possibly related to the investigated treatment} in the present and the former studies^12^ was 4/3/2/1 in the UA-ADRCs group and 3/0/0/2 in the corticosteroid group. These data were not significantly different between the groups (Chi-square test for trend; p = 0.757) (details in Supplementary Table S10 online).

The four severe TEAEs that occurred in the UA-ADRCs group during the present and the former studies^12^ were non ST elevation myocardial infarction, ST elevation myocardial infarction, ganglion cyst of the non-index shoulder, and pain in the index shoulder. None of these severe TEAEs were related to treatment (details in Supplementary Tables S11–S13 online).

### Long-term efficacy of treating sPTRCT with injection of either UA-ADRCs or corticosteroid

Four of the 11 subjects (36.4%) in the UA-ADRCs group and three of the five subjects (60.0%) in the corticosteroid group developed additional pathologies of the index shoulder (next to sPTRCT) and/or received additional injections into or surgery of the index shoulder (next to injection of either UA-ADRCs or corticosteroid) during the present and the former studies^12^. For one of the 11 subjects (9%) in the UA-ADRCs group and two of the five subjects (40%) in the corticosteroid group these additional pathologies were considered treatment failure (details in Supplementary Tables S14 and S15 online). After these intercurrent events, individual data related to the efficacy of the investigated treatment were either missing or unsuitable for assessing treatment outcome if they had been collected after the intercurrent event during the present and the former studies^12^. These missing data were imputed according to the estimand of the present study outlined in Supplementary Part 9 online.

The individual ASES Total scores, SF-36 Total scores and VAS pain scores as a function of time post-treatment are shown in Supplementary Figs. S18–S20 online; imputation of missing data is indicated in these figures. Eight of the 11 subjects (72.7%) in the UA-ADRCs group but only one of the five subjects (20%) in the corticosteroid group reached an individual ASES Total score of at least 90 at any time of the present and the former studies^12^. An ASES Total score of 100 (representing no pain and maximum function) was reached by five of the 11 subjects (45.5%) in the UA-ADRCs group but none of the five subjects (0%) in the corticosteroid group at any time of the present and the former studies^12^.

**Figure 2:**
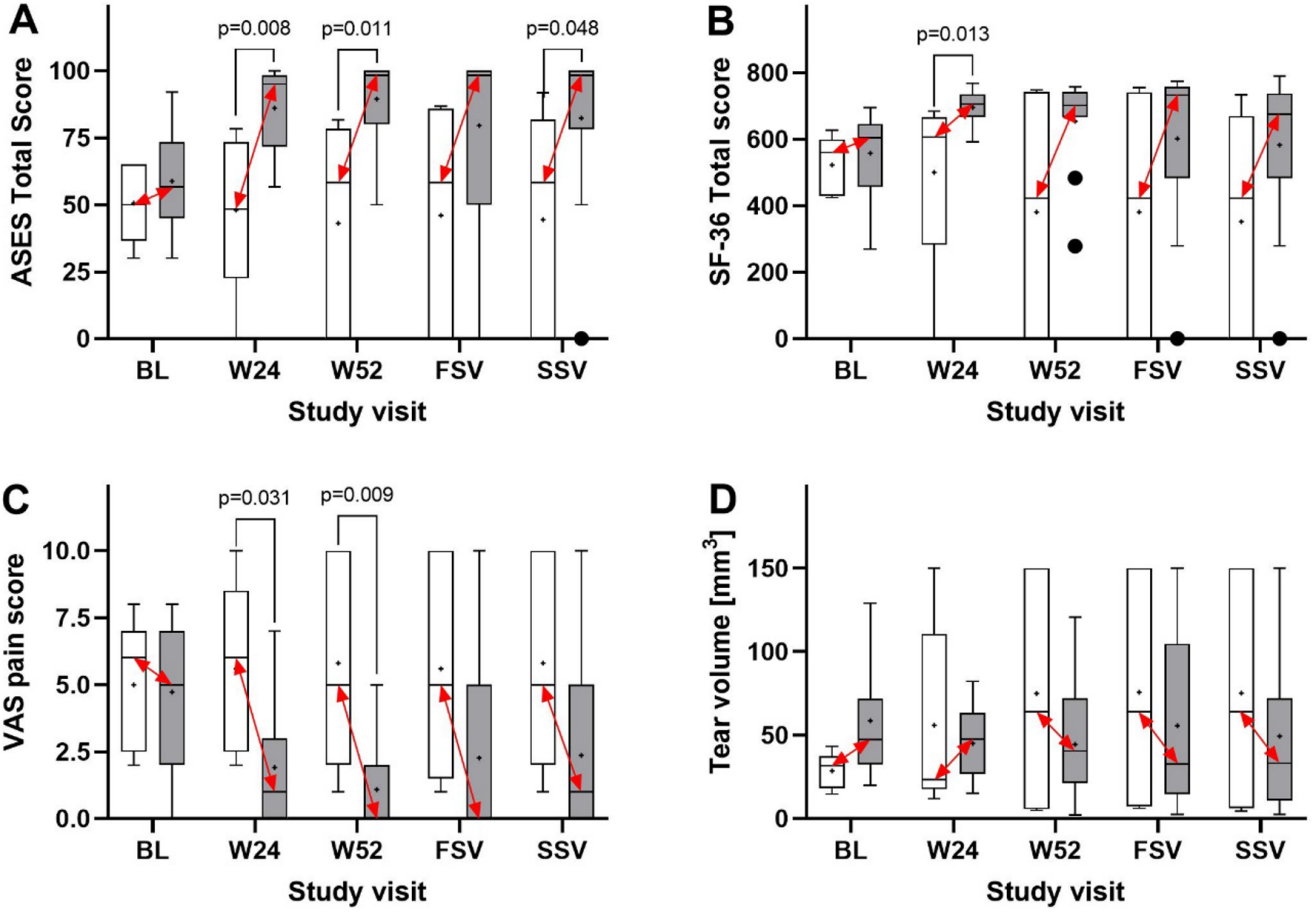
Tukey boxplots of (**A**) ASES Total score, (**B**) SF-36 Total score, (**C**) VAS Pain score (collected together with the ASES score) and (**D**) tear volume measured on MRIs of subjects treated with injection of either UA-ADRCs (gray bars) or corticosteroid (open bars). The red double-arrows indicate corresponding median values. p-values < 0.05 are indicated in (**A**–**C**); all p-values are provided in Supplementary Tables S16–S18 online. *BL* baseline, *W24/W52* study visits scheduled in the former study^12^ at 24 and 52 weeks post-treatment, *FSV* first study visit of the present study at 33.2 ± 1.0 (mean ± standard deviation) months post-treatment, *SSV* second study visit of the present study at 40.6 ± 1.9 months post-treatment.

**Figure 3:**
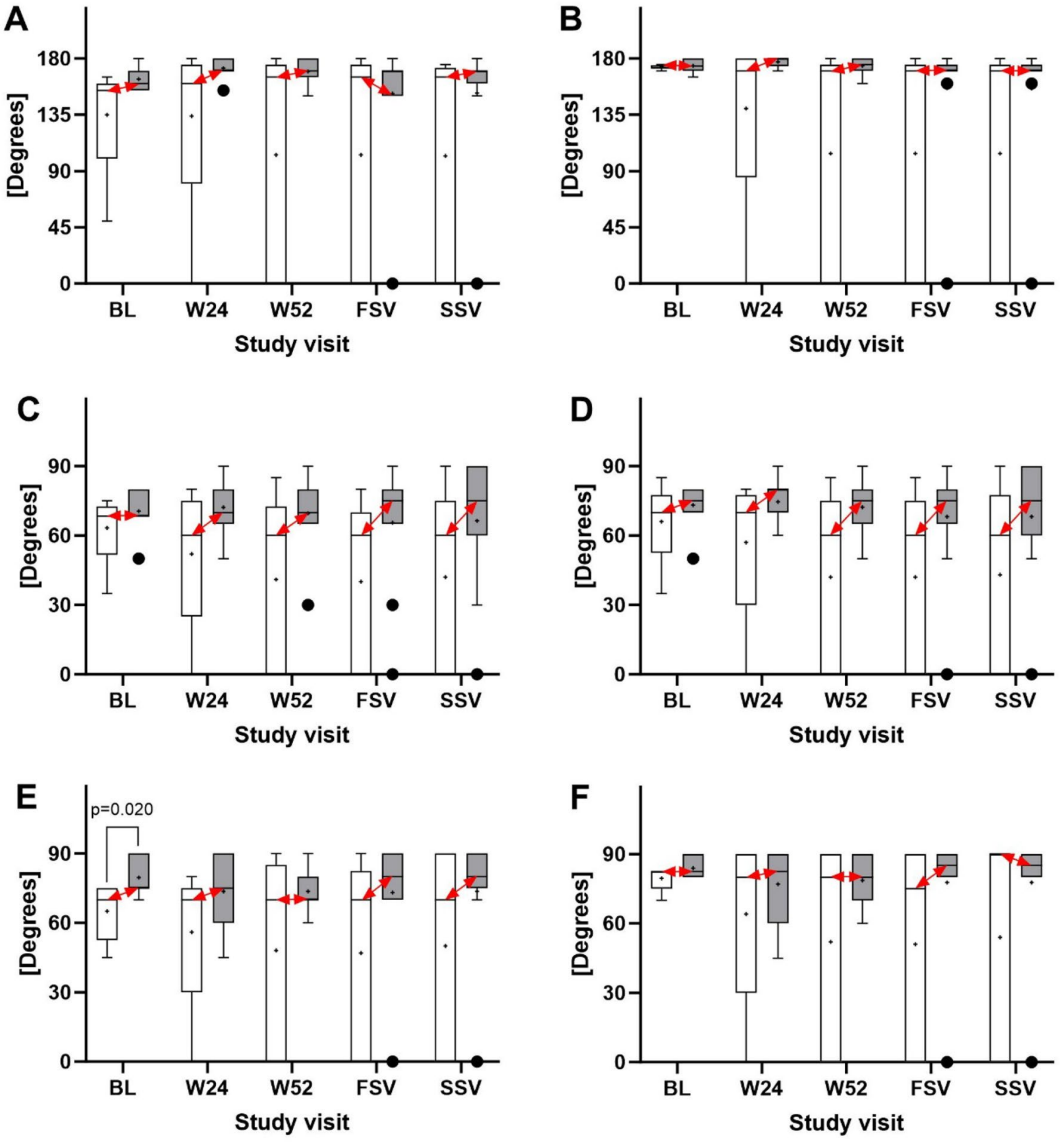
Tukey boxplots of (**A**) active and (**B**) passive forward elevation of the index arm, (**C**) active and (**D**) passive external rotation of the index arm with the latter comfortably at side, and (**E**) active and (**F**) passive external rotation of the index arm with the latter at 90° abduction of subjects treated with injection of either UA-ADRCs (gray bars) or corticosteroid (open bars). The red double-arrows indicate corresponding median values. A p-value < 0.05 is indicated in (**E**). *BL* baseline, *W24/W52* study visits scheduled in the former study^12^ at 24 and 52 weeks post-treatment, *FSV* first study visit of the present study at 33.2 ± 1.0 (mean ± standard deviation) months post-treatment, *SSV* second study visit of the present study at 40.6 ± 1.9 months post-treatment.

Statistical analysis demonstrated that compared with the subjects in the corticosteroid group, the subjects in the UA-ADRCs group had (i) a significantly higher mean ASES Total score at W24 and W52 post-treatment as well as at SSV (i.e., at 40.6 ± 1.9 months post-treatment), (ii) a significantly higher mean SF-36 Total score at W24 post-treatment, and (iii) a significantly higher mean VAS Pain score at W24 and W52 post-treatment (Fig. 2A–C and Supplementary Tables S16–S18 online). Active and passive ROM data showed no significant differences between the subjects in the UA-ADRCs group and the subjects in the corticosteroid group (Fig. 3).

### Partial thickness rotator cuff tear size as a function of time after treatment with injection of either UA-ADRCs or corticosteroid

The individual tear size as a function of time post-treatment is shown in Supplementary Fig. S21 online; imputation of missing data is indicated in this figure. Statistical analysis demonstrated no significant differences between the subjects in the UA-ADRCs group and the subjects in the corticosteroid group (Fig. 2D and Supplementary Table S19 online).

### Detection of hyperintense structures on PD FS T2 coronal MRI scans of the index shoulder at the position of the supraspinatus tendon after injection of UA-ADRCs, but not after injection of corticosteroid, at 24 weeks post-treatment but not at baseline

**Figure 4:**
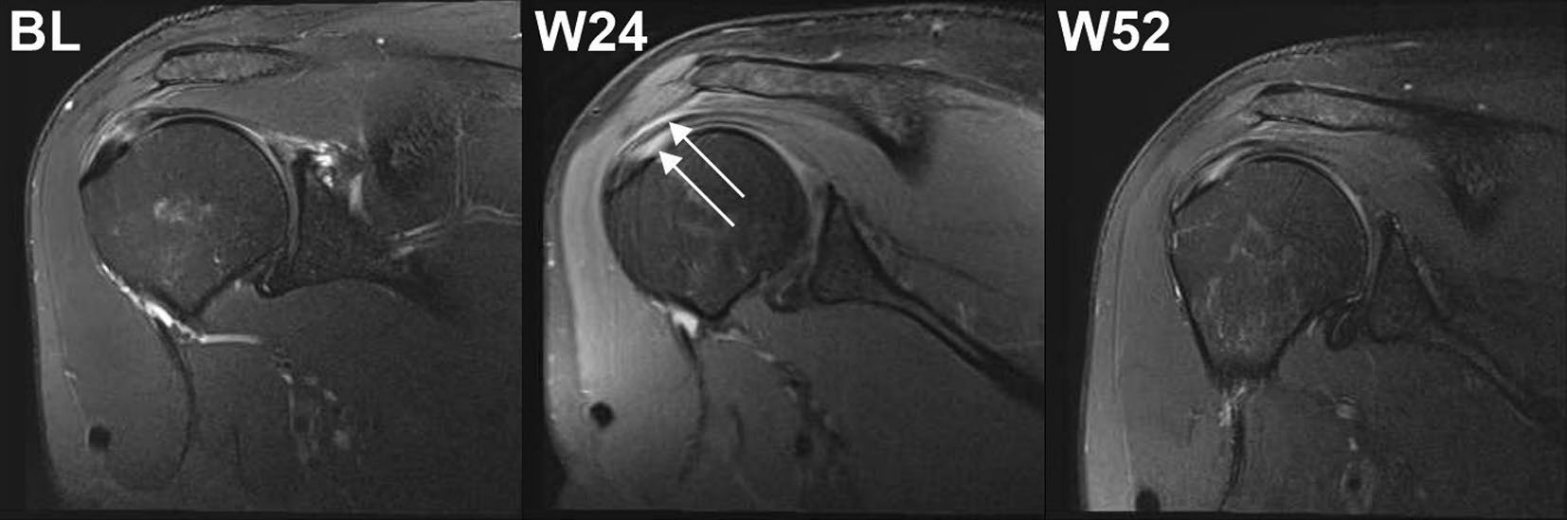
Proton density weighted, fat saturated, T2-weighted, coronal MRI scans of the index shoulder of Subject A4 (injection of UA-ADRCs), showing hyperintense structures at the position of the supraspinatus tendon at 24 weeks post-treatment (arrows) but not at baseline. *BL* baseline, *W24*/*W52* study visits scheduled in the former study^12^.

The PD FS T2 coronal MRI scans of the index shoulder of 10 of 11 subjects (90.9%) in the UA-ADRCs group and none of the subjects (0%) in the corticosteroid group showed hyperintense structures at the position of the supraspinatus tendon at W24 post-treatment but not at baseline. A representative example is shown in Fig. 4; all PD FS T2 coronal MRI scans of Subjects A1–A11 (injection of UA-ADRCs) and Subjects C1–C4 (injection of corticosteroid) are provided in Supplementary Figs. S3–S17 online. No MRI scans of Subject C5 are shown because this subject was not enrolled in the present study, and the study protocol did not allow to re-assess the MRI scans of this subject generated during the former study^12^.

### No relationship between treatment outcome and baseline data

Supplementary Figs. S22 and S23 online show individual ASES Total scores as a function of time post-treatment together with individual data at baseline (ASES Total score, tear volume, age and body mass index, as well as (in case of subjects who were treated with injection of UA-ADRCs) cell yield and cell viability. No relationship between treatment outcome and baseline data was found, including those data characterizing UA-ADRCs that can be collected with a clinical test.

## Discussion

To assess the relevance of the results of the present study in accordance with the current state of knowledge, Table 2 summarizes the essential details of all previously published clinical studies on the management of partial-thickness and full-thickness rotator cuff tears with stem cells^12,31–39^. In most of these studies, stem cells were applied to improve the outcome of surgical treatment. Furthermore, next to the former study^12^, treatment of partial-thickness rotator cuff tears was only investigated in three other studies^35,36,38^, and only three other studies were randomized controlled trials^37–39^. The mean number of subjects treated with stem cells in the studies listed in Table 2 (excluding the former study^12^) was 19.1 ± 5.0 (mean ± SEM) (median, 13.5; range, 7–45).

**Table 2:** Essential details of all published clinical studies on the management of rotator cuff tears with stem cells.

References	^31^	^32^	^33^	^34^	^35,36^^*^	^37^	^12^	^38^	^39^
Year of publication	2012	2014	2015	2017	2018, 2020	2019	2020	2022	2022
RCT	No	No	No	No	No	Yes	Yes	Yes	Yes
n (SCs/C)	14/–	45/–	8/–	35/35	13/3 + 3	8/5	11/5	7/8	23/23
Tear type	n.s.	F	F	F	P	F	P	P	F
Only SCs	No	No	No	No	Yes	No	Yes	Yes	No
Type of SCs	BM	BM	BM	UA-ADRCs	ADSCs	BM	UA-ADRCs	ADSCs	MFF
Autologous SCs	Yes	Yes	Yes	Yes	Yes	Yes	Yes	No	Yes
Surgery adjunct	Yes	Yes	Yes	Yes	No	Yes	No	No	Yes
Arthroscopy	n/a	n/a	n/a	n/a	Yes	n/a	No	No	n/a
Stat Plan	No	Yes (b)	No	Yes (b/w)	Yes (b)	Yes (w)	Yes (b/w)	Yes (b/w)	Yes (b/w)
Follow-up	M12	M120	M6	M28**	M6/M24	M12	M12	M24	M24
Outcome	n/a	n/a	n/a	No	n/a	***	Yes	No	Yes

*RCT* randomized controlled trial, *n* number of subjects, *SCs* stem cells, *C* control treatment, *n.s.* not specified, *F* full-thickness rotator cuff tears, *P* partial-thickness rotator cuff tears, *BM* bone marrow-derived mesenchymal stem cells, *UA-ADRCs* fresh, uncultured, unmodified, autologous, adipose-derived regenerative cells, *ADSCs* cultured adipose-derived stem cells, *MFF* microfragmented fat, *n/a* not applicable, *Stat Plan* statistical analysis of differences between (b) and/or within (w) the groups to assess the therapeutic outcome achieved, *M12*/*M120*/*M6*/*M29*/*M24* 12/120/6/29/24 months post-treatment, *M24 follow-up study of^35^, **28.3 ± 3.8 months in the injection group and 28.8 ± 4.2 months in the conventional group, ***this study was stopped due to adverse effects observed in both groups; most probably the reason was the use of a xenogenic scaffold (OrthADAPT Bioimplant; Synovis Orthopedic and Woundcare Inc., Irvine, CA, USA) in both groups.

With respect to the long-term safety we evidenced that treatment of sPTRCT with injection of UA-ADRCs did not result in serious adverse events by 40.6 ± 1.9 months post-treatment. There were no greater risks connected with injection of UA-ADRCs than those connected with injection of corticosteroid in treatment of sPTRCT. In summary, the results of the present study suggest that the use of UA-ADRCs in subjects with sPTRCT is safe. The only other study listed in Table 2 (excluding the former study^12^) in which UA-ADRCs were applied^34^ did not address the safety of the procedure.

Regarding the long-term efficacy, we evidenced that the subjects in the UA-ADRCs group had significantly higher mean ASES Total scores than the subjects in the corticosteroid group at W24 and W52 post-treatment as well as at the second study visit of the present study, significantly higher SF-36 Total scores at W24 post-treatment, and significantly higher VAS-Pain scores at W24 and W52 post-treatment. These findings were in line with the findings of the former study^12^ that treatment of sPTRCT with injection of UA-ADRCs leads to improved shoulder function. We hypothesize that the negative outcome (i.e., no significant difference in mean data between the groups) observed for the ASES Total score at the first study visit of the present study as well as the SF-36 Total score and the VAS Pain score at the first and second study visits of the present study were consequent to the small sample size. Using an adequate sample size of n = 246 subjects an ongoing pivotal clinical trial is currently testing the hypothesis that treatment of sPTRCT with injection of UA-ADRCs is more effective than treatment of sPTRCT with injection of corticosteroid^40^.

We would like to point to the following, additional results of the present and the former studies^12^: (i) The results obtained in the former study^12^ after treatment of sPTRCT with injection of corticosteroid were in line with other studies which investigated the efficacy of treating sPTRCT with injection of corticosteroid^41–45^ (details in Supplementary Tables S20 and S21 online). (ii) Six subjects in the UA-ADRCs group but no subject in the corticosteroid group reached an ASES Total score of 100 over time after treatment (Supplementary Fig. S18 online). Among these six subjects in the UA-ADRCs group, five reported an ASES Total score of 100 both at the end of the former study^12^ and throughout the present study (Supplementary Fig. S18 online). (iii) One subject in the corticosteroid group (Subject C5 in Supplementary Figs. S18–S20 online) developed a full thickness tear during the former study^12^, and another subject in the corticosteroid group (Subject C4 in Supplementary Figs. S18–S20 online) developed pain in the index shoulder at 1.4 months post-treatment and was treated with another injection of corticosteroid at 7.4 months post-treatment during the former study^12^. In contrast, except for one subject in the UA-ADRCs group (Subject A9 in Supplementary Figs. S18–S20 online) who reported an accident with involvement of the index shoulder at 1.0 months post-treatment, no subject in the UA-ADRCs group required additional treatment of the index shoulder during the former study^12^. In summary, these results reinforce the general need to individually examine the clinical course after an initial treatment, and to identify all possible interfering influences that could have negatively impacted the success of the therapy under study. Furthermore, these results support our hypothesis that treatment of UA-ADRCs with injection of sPTRCT is effective, and is more effective than treatment of sPTRCT with injection of corticosteroid.

The only other published study to date that investigated treatment of sPTRCT with injection of stem cells without surgery found no benefit of injection of cultured adipose-derived stem cells (ADSCs) for 24 months post-treatment, and the results obtained after injection of ADSCs did not differ from the results obtained after injection of saline^38^. This negative result could have been caused by at least three circumstances: (i) the use of allogeneic cells, with the possible inability of new cells derived from the stem cells to integrate into the host tissue because of immunological incompatibility^14^; (ii) the need for culturing the cells, with the possible reduction of the life span of the cells by shortening the telomeres following repetitive cell divisions, and possible negative effects on the safety of the cells as a medicinal product^13^; and (iii) the selection of a single cell type, with the consequence of limited functionality of the cells^14,46^. All this is prevented by the use of fresh UA-ADRCs in the present and the former studies^12^, and may explain the discrepancy between the negative result in^38^ and the positive results in the present and the former studies^12^.

It is beyond the scope of the present study to provide a comprehensive explanation why selection of stem cells (i.e., the use of cultured ADSCs or cultured MSCs in general) is inferior to the use of fresh UA-ADRCs in treatment of musculoskeletal pathologies. Here we report just three of the most important reasons: (i) unlike cultured ADSCs, fresh UA-ADRCs express those growth factors that are needed to stimulate cultured ADSCs towards tenogenic differentiation in culture^47^; (ii) these growth factors are expressed by M2 macrophages^48–51^, and M2 macrophages are contained in the UA-ADRCs used in the present and the former studies^12,20^, but are missing in any cultured stem cells; and (iii) M2 macrophages are mainly involved in anti-inflammatory responses^52,53^, and the presence of M2 macrophages in UA-ADRCs may explain the very early treatment success observed after treating sPTRCT with UA-ADRCs in the former study^12^, which cannot be explained by the formation of new tendon tissue (Supplementary Fig. S18 online). In summary, there are a number of possible explanations of the discrepancy between the negative result in^38^ and the positive results in the present and the former studies^12^ with respect to treatment of sPTRCT with injection of stem cells.

Regarding the analysis of MRIs pre- and post-injection, we found no significant improvement of the mean tear volume over time, nor any significant difference between the results obtained after injection of UA-ADRCs and those obtained after injection of corticosteroid (Supplementary Table S19 online). Of note, these findings are not in line with the results related to the long term efficacy (improvement in ASES Total score) outlined above. The main reason for this discrepancy may be the mechanisms of action of UA-ADRCs in tendon repair. Initially one could assume that UA-ADRCs would mainly fill the gap in the tendon tissue caused by a partial-thickness tear. However, the location of the hyperintense structures in PD FS T2 MRI scans at the position of the supraspinatus tendon present at 24 weeks post-treatment but not at baseline in 10 of the 11 subjects in the UA-ADRCs group (Supplementary Figs. S3–S13 online) and none of the subjects in the corticosteroid group (Supplementary Figs. S14–S17 online) indicate that this may not be the case. Rather, these hyperintense structures in PD FS T2 MRI scans may indicate formation of new tendon tissue following injection of UA-ADRCs in a different location than the original tear, possibly primarily following individual biomechanical requirements. This may explain why subjects who are suffering from sPTRCT experience fast (the former study^12^) and lasting (the present study) recovery from pain and impaired function without disappearance of the rotator cuff tears on MRI scans even at 41 months post-treatment.

The presence of hyperintense structures in PD FS T2 MRI scans at the position of a tendon with partial-thickness tear a few months after injection of UA-ADRCs has only been reported in a recent single case report^54^. Without additional investigations, it is unclear whether these hyperintense structures in PD FS T2 MRI scans indeed represent formation of new tendon tissue. These investigations must be performed on biopsies of tendons with partial-thickness tear that were treated with injection of UA-ADRCs. On the other hand, there are two indications supporting the hypothesis that these hyperintense structures in PD FS T2 MRI scans indeed represent formation of new tendon tissue: (i) the analysis of the biopsy reported in the recent case report^54^ indicated newly formed tendon tissue which did not resemble scar tissue (the biopsy was taken at the position of the hyperintense structure found in the corresponding MRI scans 10 weeks post-treatment); and (ii) this biopsy showed a dense network of newly formed microvessels next to the position of newly formed tendon tissue^54^. Blood flow in these newly formed microvessels may indeed explain the occurrence of hyperintense structures in PD FS T2 MRI scans after treatment of sPTRCT with injection of UA-ADRCs. Furthermore, the full or partial disappearance of these hyperintense structures in PD FS T2 MRI scans at 52 weeks post-treatment (Supplementary Figs. S3–S13 online) may indicate that tendon regeneration was complete, or almost complete, at this time.

In summary, the results of the present study indicate that treatment success after treating sPTRCT with UA-ADRCs cannot be assessed using measurements of tear volume on MRI scans. On the other hand, PD FS T2 MRI scans taken a few months after treatment of sPTRCT with injection of UA-ADRCs may allow to “watch the UA-ADRCs at work”. The latter finding may inform researchers about optimal times for taking biopsies in future research into the mechanisms of action of UA-ADRCs in tendon repair.

Based on the outcome of the analysis shown in Supplementary Fig. S22 online we hypothesize that individual treatment success after treating sPTRCT with injection of UA-ADRCs cannot be predicted based on the following, individual values at baseline: ASES Total score, tear volume, age and BMI, as well as on the cell yield and cell viability of the final cell suspension. This finding is important because it may render individual bedside testing of the final cell suspension in clinical use of UA-ADRCs irrelevant.

It is currently unknown whether individual treatment success after treating sPTRCT with injection of UA-ADRCs can be predicted using the colony forming unit (CFU) assay^15^ and/or determination of cell surface markers using fluorescence-activated cell scanning^20^. In any case, these analyses take between several days (determination of surface markers) and more than 2 weeks (CFU assay). Thus, they are not suitable for clinical testing of the final cell suspension in clinical use of UA-ADRCs.

The limitations of the present study are the same as the limitations of the former study^12^: only a small sample of subjects suffering from sPTRCT was investigated, only a limited number of clinical examination methods was applied, no power analysis was carried out, and neither the subjects nor the physicians who performed treatment and the assessors who performed baseline and follow-up examinations were blinded (only the physicians who analyzed the MRI scans were blinded). We believe that the ongoing clinical trial^40^ will demonstrate with sufficient statistical power that treatment of sPTRCT with injection of UA-ADRCs is more effective than treatment of sPTRCT with injection of corticosteroid.

## Conclusions

The present investigation further supports treatment of sPTRCT with injection of UA-ADRCs. Once this therapy is approved in the US, clinicians should consider injection of UA-ADRCs instead of injection of corticosteroids. In the long run treatment of sPTRCT with injection of UA-ADRCs may delay or even prevent surgical treatment of sPTRCT.

### Supplementary Information

Supplementary Information.

## Data Availability

The datasets used and analyzed during the current study are available from the corresponding author on reasonable request, taking into account any confidentiality.

## References

[1] Matava MJ, Purcell DB, Rudzki JR (2005). Partial-thickness rotator cuff tears. Am. J. Sports Med..

[2] Matthewson G (2015). Partial thickness rotator cuff tears: Current concepts. Adv. Orthop..

[3] Via AG, De Cupi SM, Spoliti M, Oliva F (2013). Clinical and biological aspects of rotator cuff tears. Muscles Ligaments Tendons J..

[4] Cotton RE, Rideout DF (1964). Tears of the humeral rotator cuff. J. Bone Joint Surg..

[5] Fukuda H (2000). Partial-thickness rotator cuff tears: A modern view on Codman's classic. J. Shoulder Elbow Surg..

[6] Sher JS, Uribe JW, Posada A, Murphy BJ, Zlatkin MB (1995). Abnormal findings on magnetic resonance images of asymptomatic shoulders. J. Bone Joint Surg. Am..

[7] Coombes BK, Bisset L, Vicenzino B (2010). Efficacy and safety of corticosteroid injections and other injections for management of tendinopathy: A systematic review of randomised controlled trials. Lancet.

[8] Ramírez J (2014). Incidence of full-thickness rotator cuff tear after subacromial corticosteroid injection: A 12-week prospective study. Mod. Rheumatol..

[9] Hurley ET (2019). Nonoperative treatment of rotator cuff disease with platelet-rich plasma: A systematic review of randomized controlled trials. Arthroscopy.

[10] Schwitzguebel AJ (2019). Efficacy of platelet-rich plasma for the treatment of interstitial supraspinatus tears: A double-blinded, randomized controlled trial. Am. J. Sports Med..

[11] Kukkonen J (2014). Treatment of non-traumatic rotator cuff tears: A randomised controlled trial with one-year clinical results. Bone Joint J..

[12] Hurd JL (2020). Safety and efficacy of treating symptomatic, partial-thickness rotator cuff tears with fresh, uncultured, unmodified, autologous adipose-derived regenerative cells (UA-ADRCs) isolated at the point of care: A prospective, randomized, controlled first-in-human pilot study. J. Orthop. Surg. Res..

[13] Cossu G (2018). Lancet Commission: Stem cells and regenerative medicine. Lancet.

[14] Furia JP (2022). Why and how to use the body's own stem cells for regeneration in musculoskeletal disorders: A primer. J. Orthop. Surg. Res..

[15] Winnier GE (2019). Isolation of adipose tissue derived regenerative cells from human subcutaneous tissue with or without the use of an enzymatic reagent. PLoS One.

[16] Aust L (2004). Yield of human adipose-derived adult stem cells from liposuction aspirates. Cytotherapy.

[17] Yu H, Lu K, Zhu J, Wang J (2017). Stem cell therapy for ischemic heart diseases. Br. Med. Bull..

[18] Zhu Y (2008). Adipose-derived stem cell: A better stem cell than BMSC. Cell Biochem. Funct..

[19] Boutron I (2017). CONSORT statement for randomized trials of nonpharmacologic treatments: A 2017 update and a CONSORT extension for nonpharmacologic trial abstracts. Ann. Intern. Med..

[20] Schmitz C (2022). The composition of adipose-derived regenerative cells isolated from lipoaspirate using a point of care system does not depend on the subject's individual age, sex, body mass index and ethnicity. Cells.

[21] Angst F, Schwyzer HK, Aeschlimann A, Simmen BR, Goldhahn J (2011). Measures of adult shoulder function: Disabilities of the Arm, Shoulder, and Hand Questionnaire (DASH) and its short version (QuickDASH), Shoulder Pain and Disability Index (SPADI), American Shoulder and Elbow Surgeons (ASES) Society standardized shoulder assessment form, Constant (Murley) Score (CS), Simple Shoulder Test (SST), Oxford Shoulder Score (OSS), Shoulder Disability Questionnaire (SDQ), and Western Ontario Shoulder Instability Index (WOSI). Arthritis Care Res..

[22] Hays RD, Sherbourne CD, Mazel RM (1993). The RAND 36-Item Health Survey 1.0. Health Econ..

[23] Moore W, Frye S (2020). Review of HIPAA, Part 2: Limitations, rights, violations, and role for the imaging technologist. J. Nucl. Med. Technol..

[24] U.S. Food and Drug Administration. E9(R1) statistical principles for clinical trials: Addendum: Estimands and sensitivity analysis in clinical trials https://www.fda.gov/regulatory-information/search-fda-guidance-documents/e9r1-statistical-principles-clinical-trials-addendum-estimands-and-sensitivity-analysis-clinical (2021).

[25] European Medicines Agency. ICH E9 (R1) addendum on estimands and sensitivity analysis in clinical trials to the guideline on statistical principles for clinical trials https://www.ema.europa.eu/en/documents/scientific-guideline/ich-e9-r1-addendum-estimands-sensitivity-analysis-clinical-trials-guideline-statistical-principles_en.pdf (2020).

[26] Cro S (2022). Evaluating how clear the questions being investigated in randomised trials are: Systematic review of estimands. Br. Med. J..

[27] Salkind NJ (2010). Encyclopedia of Research Design.

[28] Fletcher C, Tsuchiya S, Mehrotra DV (2017). Current practices in choosing estimands and sensitivity analyses in clinical trials: Results of the ICH E9 Survey. Ther. Innov. Regul. Sci..

[29] Greenspoon JA, Petri M, Warth RJ, Millett PJ (2015). Massive rotator cuff tears: Pathomechanics, current treatment options, and clinical outcomes. J. Shoulder Elbow Surg..

[30] American Academy of Orthopaedic Surgeons. Management of rotator cuff injuries. Evidence-based clinical practice guideline. http://www.aaos.org/rccpg (2019).

[31] Ellera Gomes JL, da Silva RC, Silla LM, Abreu MR, Pellanda R (2012). Conventional rotator cuff repair complemented by the aid of mononuclear autologous stem cells. Knee Surg. Sports Traumatol. Arthrosc..

[32] Hernigou P (2014). Biologic augmentation of rotator cuff repair with mesenchymal stem cells during arthroscopy improves healing and prevents further tears: A case-controlled study. Int. Orthop..

[33] Havlas V (2015). Kultivovaných lidských autologních kmenových buněk kostní dřeně při rekonstrukci ruptury rotátorové manžety—studie bezpečnosti metody, předběžné výsledky (Czech) [Use of cultured human autologous bone marrow stem cells in repair of a rotator cuff tear: Preliminary results of a safety study]. Acta Chir. Orthop. Traumatol. Cech..

[34] Kim YS, Sung CH, Chung SH, Kwak SJ, Koh YG (2017). Does an injection of adipose-derived mesenchymal stem cells loaded in fibrin glue influence rotator cuff repair outcomes? A clinical and magnetic resonance imaging study. Am. J. Sports Med..

[35] Jo CH (2018). Intratendinous injection of autologous adipose tissue-derived mesenchymal stem cells for the treatment of rotator cuff disease: A first-in-human trial. Stem Cells.

[36] Jo CH, Chai JW, Jeong EC, Oh S, Yoon KS (2020). Intratendinous injection of mesenchymal stem cells for the treatment of rotator cuff disease: A 2-year follow-up study. Arthroscopy.

[37] Lamas JR (2019). Adverse effects of xenogenic scaffolding in the context of a randomized double-blind placebo-controlled study for repairing full-thickness rotator cuff tears. Trials.

[38] Chun SW (2022). A randomized controlled trial of stem cell injection for tendon tear. Sci. Rep..

[39] Randelli PS (2022). Arthroscopic rotator cuff repair augmentation with autologous microfragmented lipoaspirate tissue is safe and effectively improves short-term clinical and functional results: A prospective randomized controlled trial with 24-month follow-up. Am. J. Sports Med..

[40] Hurd, J. Autologous adult adipose-derived regenerative cell injection into chronic partial-thickness rotator cuff tears. https://www.clinicaltrials.gov/ct2/show/NCT03752827 (2018).

[41] Alvarez CM, Litchfield R, Jackowski D, Griffin S, Kirkley A (2005). A prospective, double-blind, randomized clinical trial comparing subacromial injection of betamethasone and xylocaine to xylocaine alone in chronic rotator cuff tendinosis. Am. J. Sports Med..

[42] von Wehren L (2016). The effect of subacromial injections of autologous conditioned plasma versus cortisone for the treatment of symptomatic partial rotator cuff tears. Knee Surg. Sports Traumatol. Arthrosc..

[43] Cole B, Lam P, Hackett L, Murrell GAC (2018). Ultrasound-guided injections for supraspinatus tendinopathy: Corticosteroid versus glucose prolotherapy—A randomized controlled clinical trial. Shoulder Elbow.

[44] Damjanov N (2018). The efficacy and safety of autologous conditioned serum (ACS) injections compared with betamethasone and placebo injections in the treatment of chronic shoulder joint pain due to supraspinatus tendinopathy: A prospective, randomized, double-blind, controlled study. Med. Ultrason..

[45] Sari A, Eroglu A (2020). Comparison of ultrasound-guided platelet-rich plasma, prolotherapy, and corticosteroid injections in rotator cuff lesions. J. Back Musculoskelet. Rehabil..

[46] Andia I, Maffulli N, Burgos-Alonso N (2019). Stromal vascular fraction technologies and clinical applications. Expert Opin. Biol. Ther..

[47] Polly SS (2019). Adipose-derived stromal vascular fraction and cultured stromal cells as trophic mediators for tendon healing. J. Orthop. Res..

[48] Dai M, Sui B, Xue Y, Liu X, Sun J (2018). Cartilage repair in degenerative osteoarthritis mediated by squid type II collagen via immunomodulating activation of M2 macrophages, inhibiting apoptosis and hypertrophy of chondrocytes. Biomaterials.

[49] Liu Z, Kuang W, Zhou Q, Zhang Y (2018). TGF-β1 secreted by M2 phenotype macrophages enhances the stemness and migration of glioma cells via the SMAD2/3 signalling pathway. Int. J. Mol. Med..

[50] Lv J (2021). M2-like tumour-associated macrophage-secreted IGF promotes thyroid cancer stemness and metastasis by activating the PI3K/AKT/mTOR pathway. Mol. Med. Rep..

[51] Spiller KL (2014). The role of macrophage phenotype in vascularization of tissue engineering scaffolds. Biomaterials.

[52] Yunna C, Mengru H, Lei W, Weidong C (2020). Macrophage M1/M2 polarization. Eur. J. Pharmacol..

[53] Scala P (2021). Stem cell and macrophage roles in skeletal muscle regenerative medicine. Int. J. Mol. Sci..

[54] Alt E (2021). First immunohistochemical evidence of human tendon repair following stem cell injection: A case report and review of literature. World J. Stem Cells.

