# Supplementary material for: Management of partial-thickness rotator cuff tears with autologous adipose-derived regenerative cells is safe and more effective than injection of corticosteroid
Source: Sci Rep. 2023 Nov 7;13:19348. doi: 10.1038/s41598-023-46653-4 (PMC10630470; doi:10.1038/s41598-023-46653-4)
Supplement: Supplementary file 1 — Supplementary Information. [file 41598_2023_46653_MOESM1_ESM.docx]

**Supplement**

**Management of partial-thickness rotator cuff tears with autologous adipose-derived regenerative cells is safe and more effective than injection of corticosteroid**

Mark Lundeen^1^, Jason L. Hurd^2^, Matthew Hayes^3^, Meredith Hayes^3^, Tiffany R. Facile^4^, John P. Furia^5^, Nicola Maffulli^6,7,8^, Christopher Alt^9,10,11^, Eckhard U. Alt^9,11,12,13^, Christoph Schmitz^10^, David A. Pearce^4,12,14,*^

^1^ Sanford Orthopedics & Sports Medicine Fargo, Fargo, ND, USA.

^2^ Sanford Orthopedics & Sports Medicine Sioux Falls, Sioux Falls, SD, USA.

^3^ Sanford Radiology Clinic, Sioux Falls, SD, USA.

^4^ Sanford Health, Sioux Falls, SD, USA.

^5^ SUN Orthopedics of Evangelical Community Hospital, Lewisburg, PA, USA.

^6^ Department of Trauma and Orthopaedic Surgery, Sapienza University of Rome, Sant'Andrea Hospital, Rome, Italy.

^7^ Centre for Sports and Exercise Medicine, Barts and The London School of Medicine and Dentistry, Mile End Hospital, Queen Mary University of London, London, UK.

^8^ School of Pharmacy and Bioengineering, Guy Hilton Research Centre, Keele University School of Medicine, Stoke on Trent, UK.

^9^ InGeneron, Inc., Houston, TX, USA.

^10^ Institute of Anatomy, Faculty of Medicine, LMU Munich, Munich, Germany.

^11^ Isar Klinikum, Munich, Germany.

^12^ Sanford School of Medicine, University of South Dakota, Sioux Falls, SD, USA.

^13^ Heart and Vascular Institute, Department of Medicine, Tulane University Health Science Center, New Orleans, LA, USA.

^14^ Sanford Research, Sioux Falls, SD, USA.

*Correspondence:

David A. Pearce

david.pearce@sanfordhealth.org

Note: throughout this Supplement the term "former study" refers to the following study:

Hurd, J.L. *et al.* Safety and efficacy of treating symptomatic, partial-thickness rotator cuff tears with fresh, uncultured, unmodified, autologous, adipose-derived regenerative cells (UA-ADRCs) isolated at the point of care: a prospective, randomized, controlled first-in-human pilot study. *J. Orthop. Surg. Res.* **15**(1), 122 (2020).

**Part 1 –** **Isolation of unmodified, autologous, adipose-derived regenerative cells from lipoaspirate using the Transpose RT system (InGeneron, Houston, TX, USA)**

**Supplementary Figure S1.** The Transpose RT System (InGeneron). The system consists of (A) the Transpose Ultra Regenerative Cell Kit (all disposable components) and the Autoclavable Tube Rack (red arrow in A), (B) the Tissue Processing Unit and the Processing Unit Rotor (red arrow in B), and (C) the Matrase Reagent. Abbreviations: WT, wash tubes; PT, processing tubes; F, 200 µm filters; S3, 3 ml syringes; S10, 10 mL syringes; S60, 60 mL syringes; LA, luer adapter; SLA; spike luer adapter; N, needle; MV, Matrase vial; SW, sterile water. The blue and yellow arrows in (C) are explained in the legend of Supplementary Fig. S2.

**
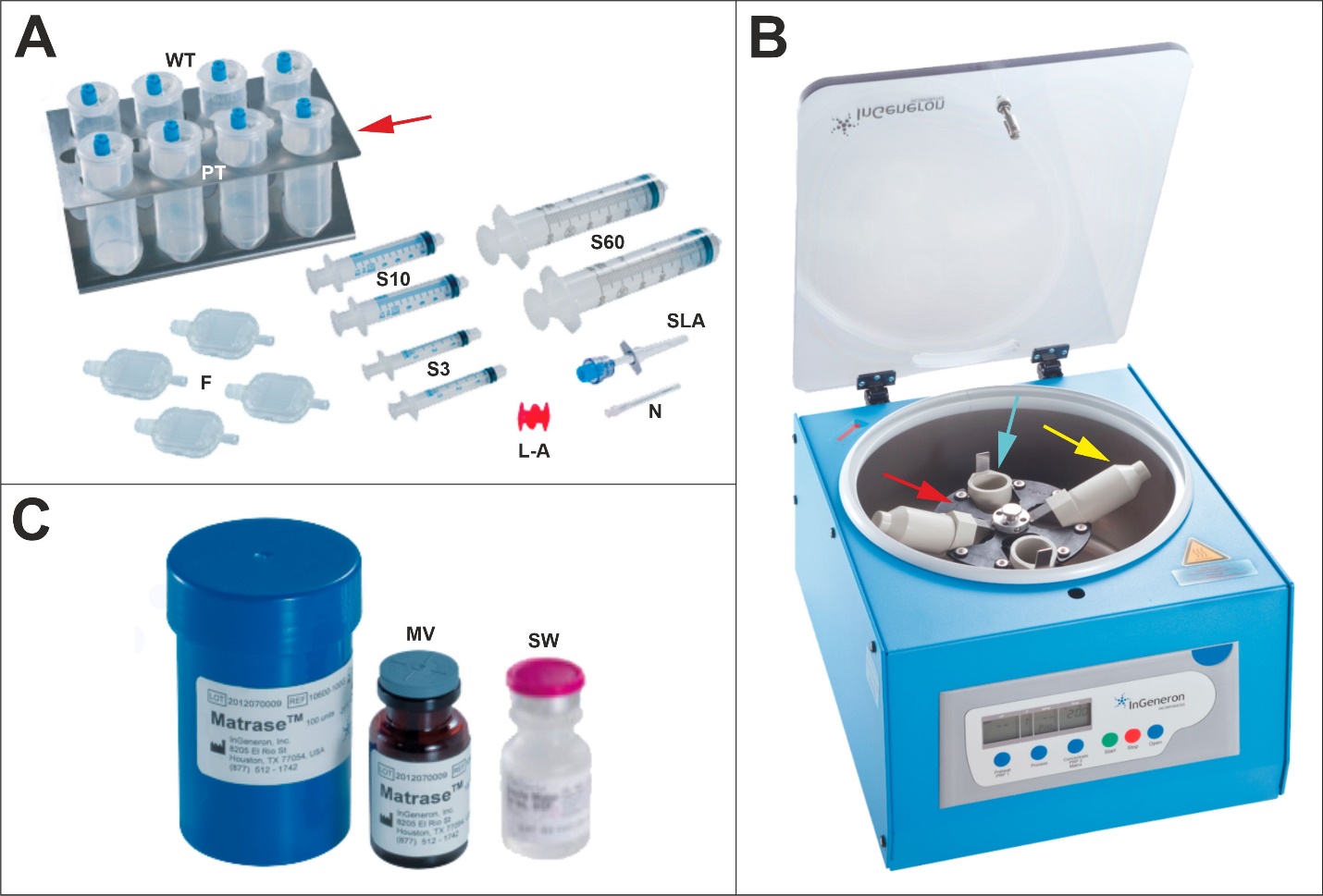
**

**Supplementary Figure S2.** Isolation of unmodified, autologous, adipose-derived regenerative cells (UA-ADRCs) from adipose tissue using the Transpose RT system (InGeneron) (modified from the former study). (A) Recovered lipoaspirate (25 ml) is loaded together with 2.5 ml reconstituted Matrase and sterile isotonic solution (such as lactated Ringer’s solution or saline) (preheated to 39° C) into a processing tube up to the MAX FILL line. (B) The filled processing tubes are subjected in an inverted position (indicated by the yellow arrow in Supplementary Fig. S1) inside the Tissue Processing Unit to repetitive acceleration and deceleration for 30 minutes at 39° C. (C) The processed lipoaspirate solution is filtered through a 200 µm filter and transferred into a wash tube. (D) After filling the wash tube with saline (room temperature) up to the MAX FILL line, the cells are separated from the rest of the tissue by centrifugation at 600g for 5 minutes at room temperature (the position of the wash tube in the Processing Unit Rotor during centrifugation is indicated by the blue arrow in Supplementary Fig. S1). (E) The UA-ADRCs (approximately 2 ml) are extracted through a swabable luer vial adapter at the bottom of the wash tube, and the remaining substances (fat, debris and liquid) are discarded. (F) The cells are returned into the empty wash tube and (after adding fresh saline up to the MAX FILL line) centrifugated again for 5 minutes. The extractions of two tubes are in this step pooled into one wash tube. (G,H) The previous washing step is repeated. (I) The concentrated UA-ADRCs (approximately 3 ml) are extracted and slowly pushed through a luer coupler into a new sterile syringe. At this point the UA-ADRCs are ready for application to the subject.


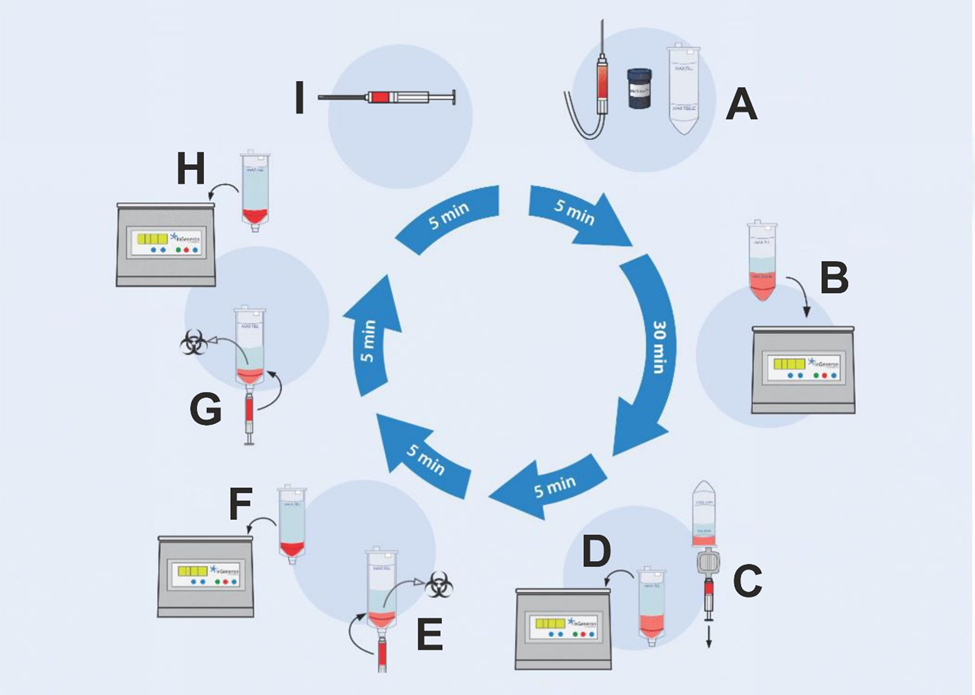


**Part 2 – Original MRI scans**

**Supplementary Figure S3.** Proton density weighted, fat saturated, T2-weighted, coronal magnetic resonance imaging (MRI) scans of the index shoulder of Subject A1 treated with injection of UA-ADRCs, generated during the present and the former studies. Panels A-L show the same (or nearly the same) image planes at different times, with Panels A showing the most ventral image plane and Panels L the most dorsal image plane. The arrows in Panels F_W24_, G_W24_, H_W24_, I_W24_ and J_W24_ indicate a hyperintense structure at the position of the supraspinatus tendon that was found at 24 weeks post-treatment but not at baseline. *BL* baseline, *W24 / W52* 24 / 52 weeks post-treatment, *M34 / M41* 34 / 41 months post-treatment.


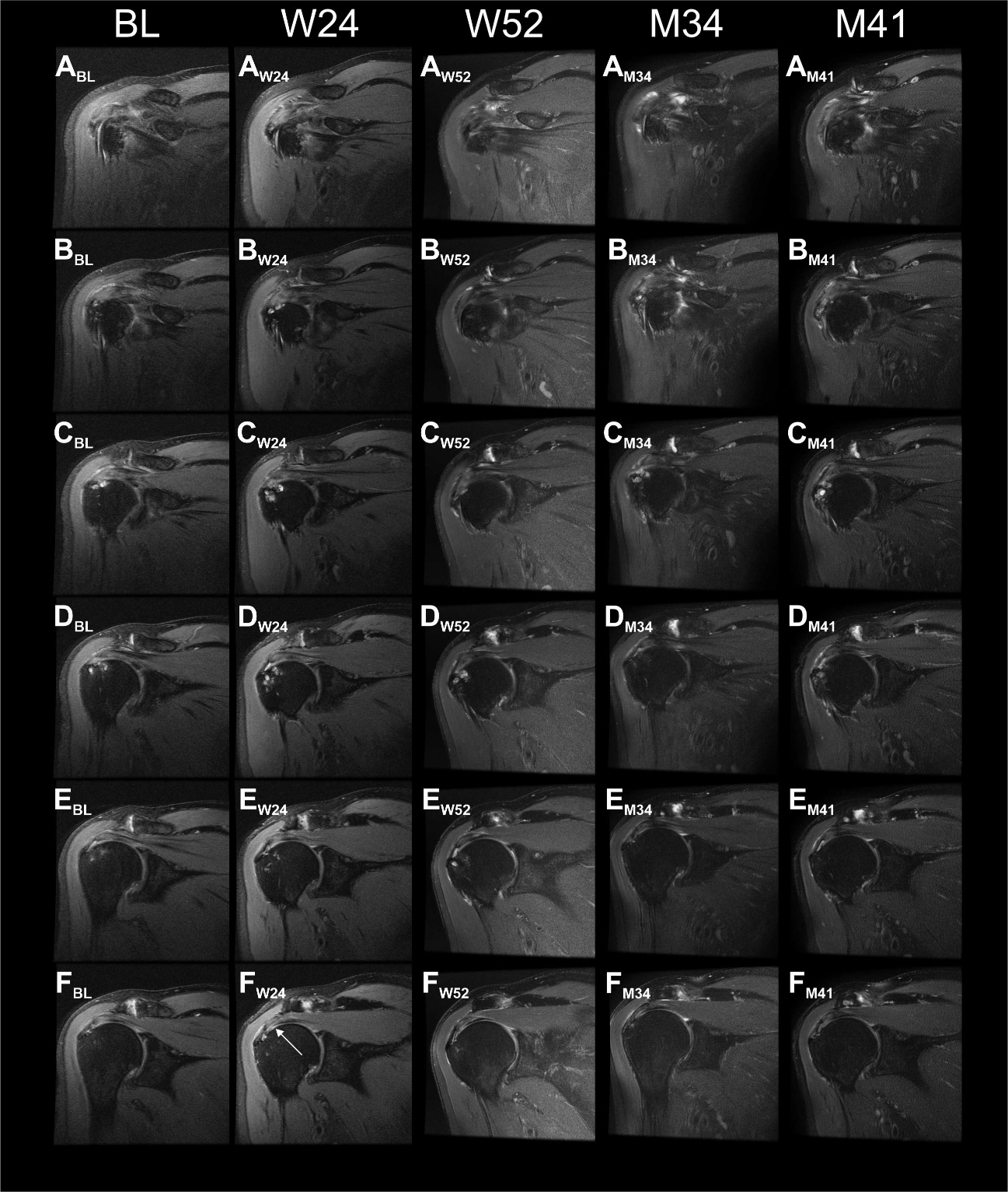


**Supplementary Figure S3 (cont.)**


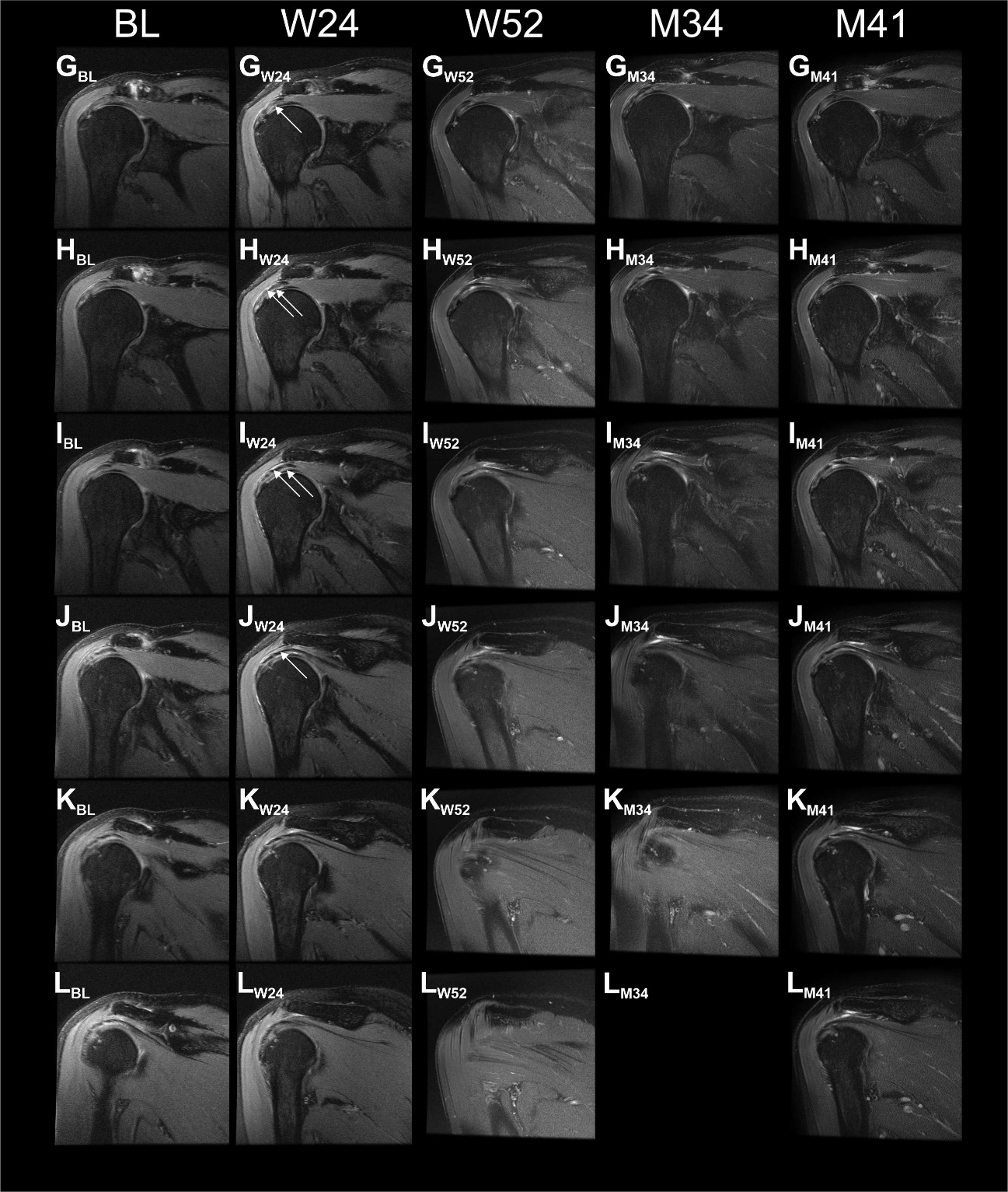


**Supplementary Figure S4.** Proton density weighted, fat saturated, T2-weighted, coronal magnetic resonance imaging (MRI) scans of the index shoulder of Subject A2 treated with injection of UA-ADRCs, generated during the present and the former studies. Panels A-L show the same (or nearly the same) image planes at different times, with Panels A showing the most ventral image plane and Panels L the most dorsal image plane. The arrows in Panels G_W24_, H_W24_ and I_W24_ indicate a hyperintense structure at the position of the supraspinatus tendon that was found at 24 weeks post-treatment but not at baseline. *BL* baseline, *W24 / W52* 24 / 52 weeks post-treatment, *M33 / M41* 33 / 41 months post-treatment.


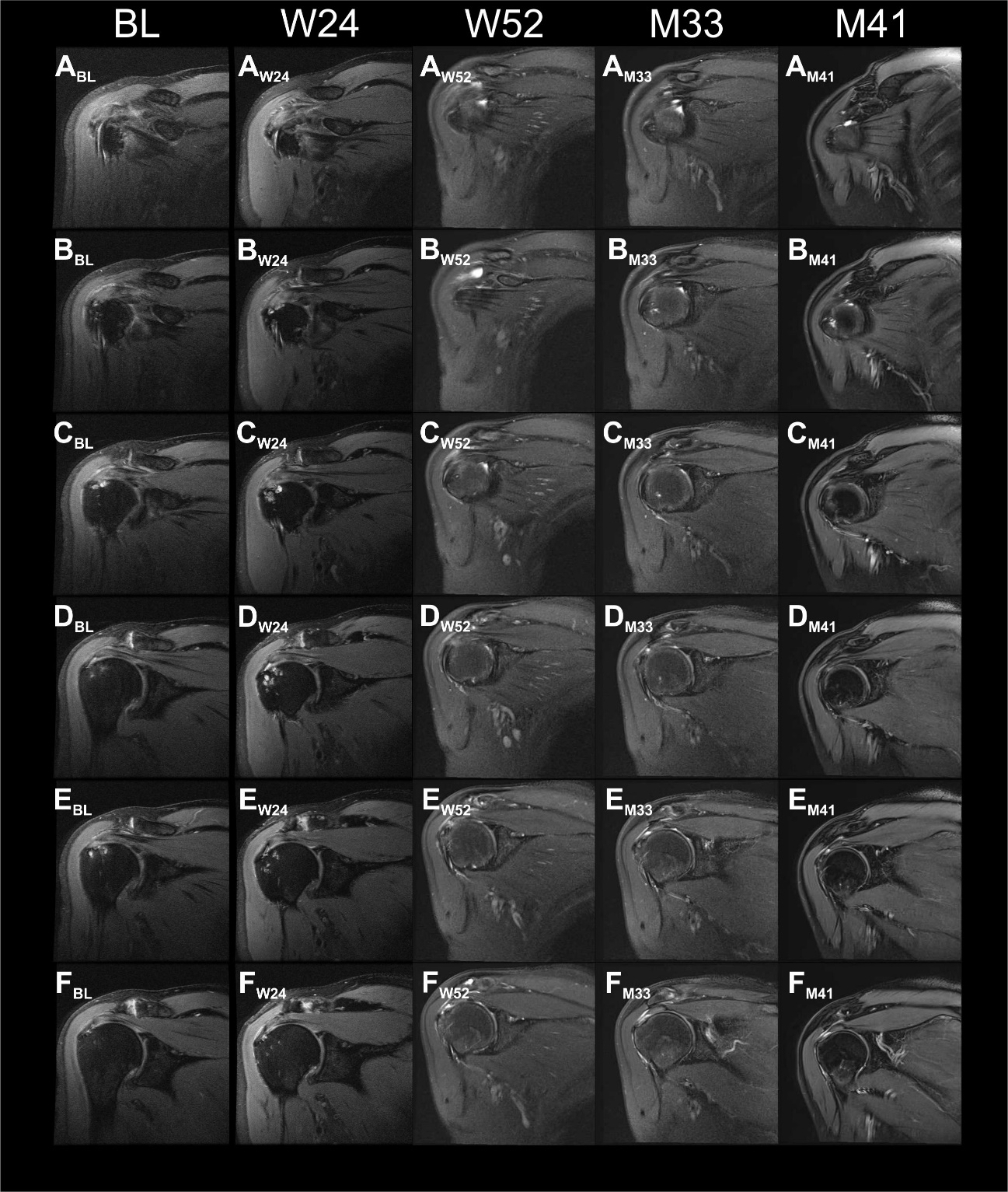


**Supplementary Figure S4 (cont.)**


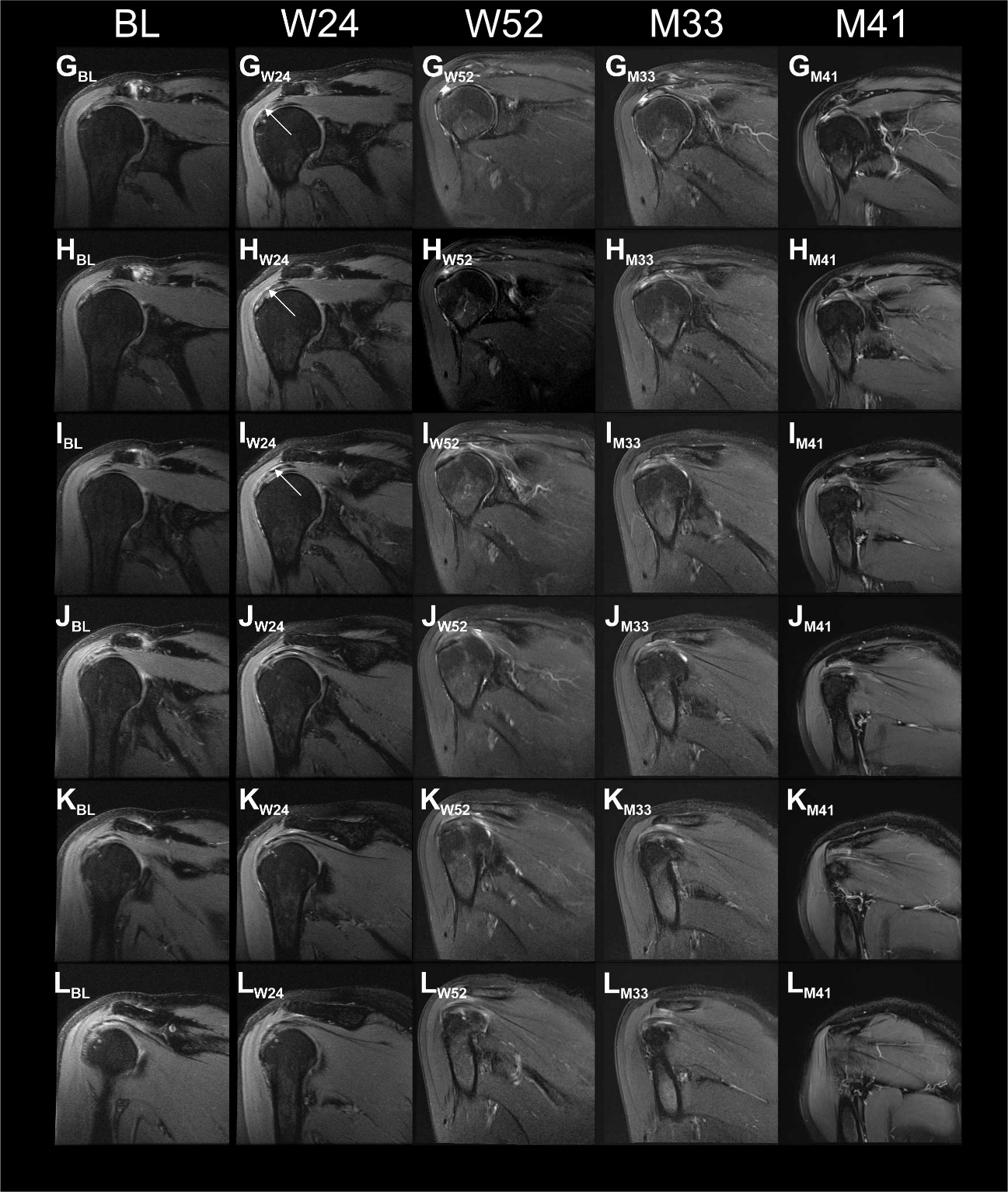


**Supplementary Figure S5.** Proton density weighted, fat saturated, T2-weighted, coronal magnetic resonance imaging (MRI) scans of the index shoulder of Subject A3 treated with injection of UA-ADRCs, generated during the present and the former studies. Panels A-L show the same (or nearly the same) image planes at different times, with Panels A showing the most ventral image plane and Panels L the most dorsal image plane. The arrows in Panels C_W24_, D_W24_, E_W24_, F_W24_, G_W24_ and H_W24_ indicate a hyperintense structure at the position of the supraspinatus tendon that was found at 24 weeks post-treatment but not at baseline. *BL* baseline, *W24 / W52* 24 / 52 weeks post-treatment, *M32 / M39* 32 / 39 months post-treatment.


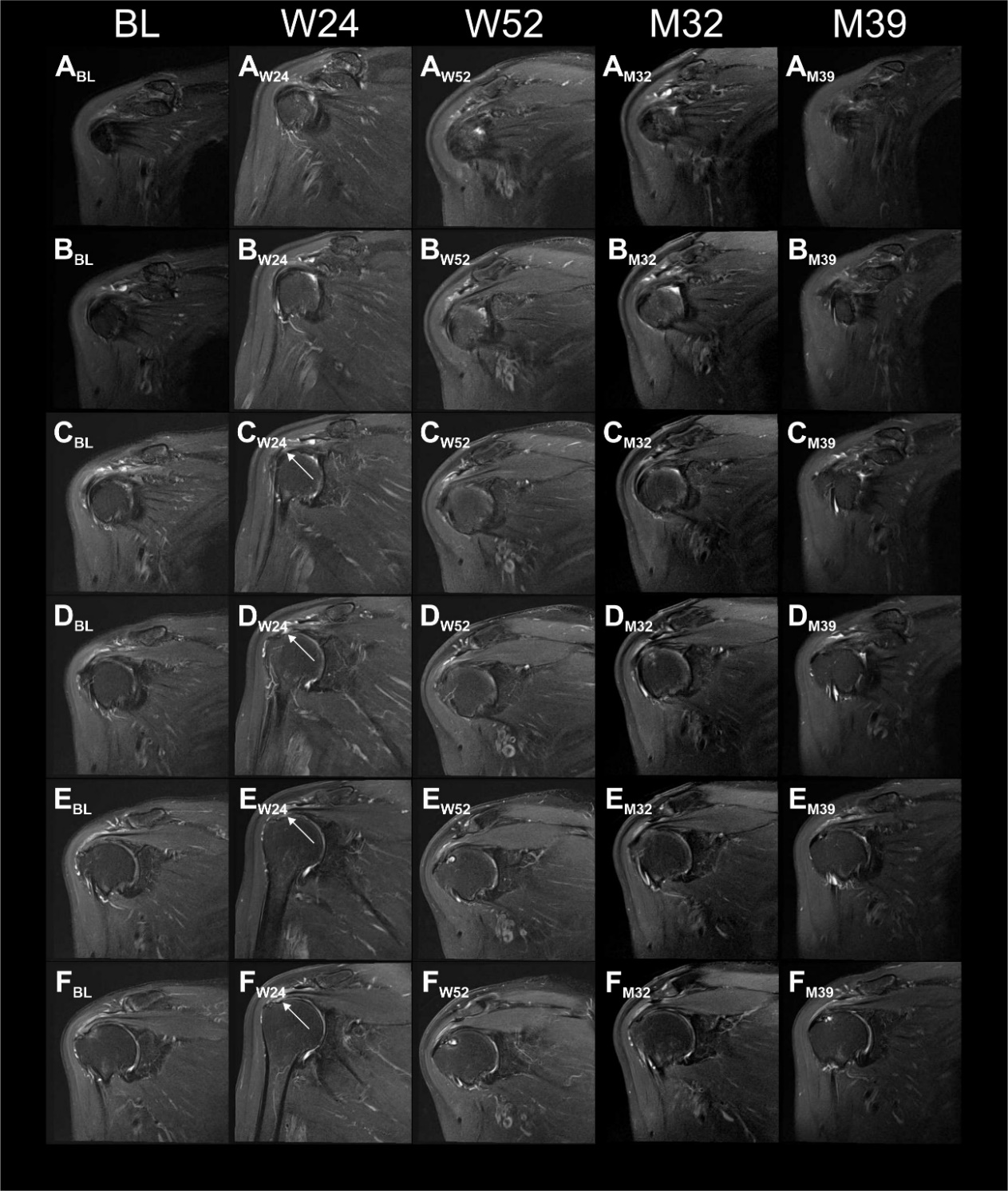


**Supplementary Figure S5 (cont.)**


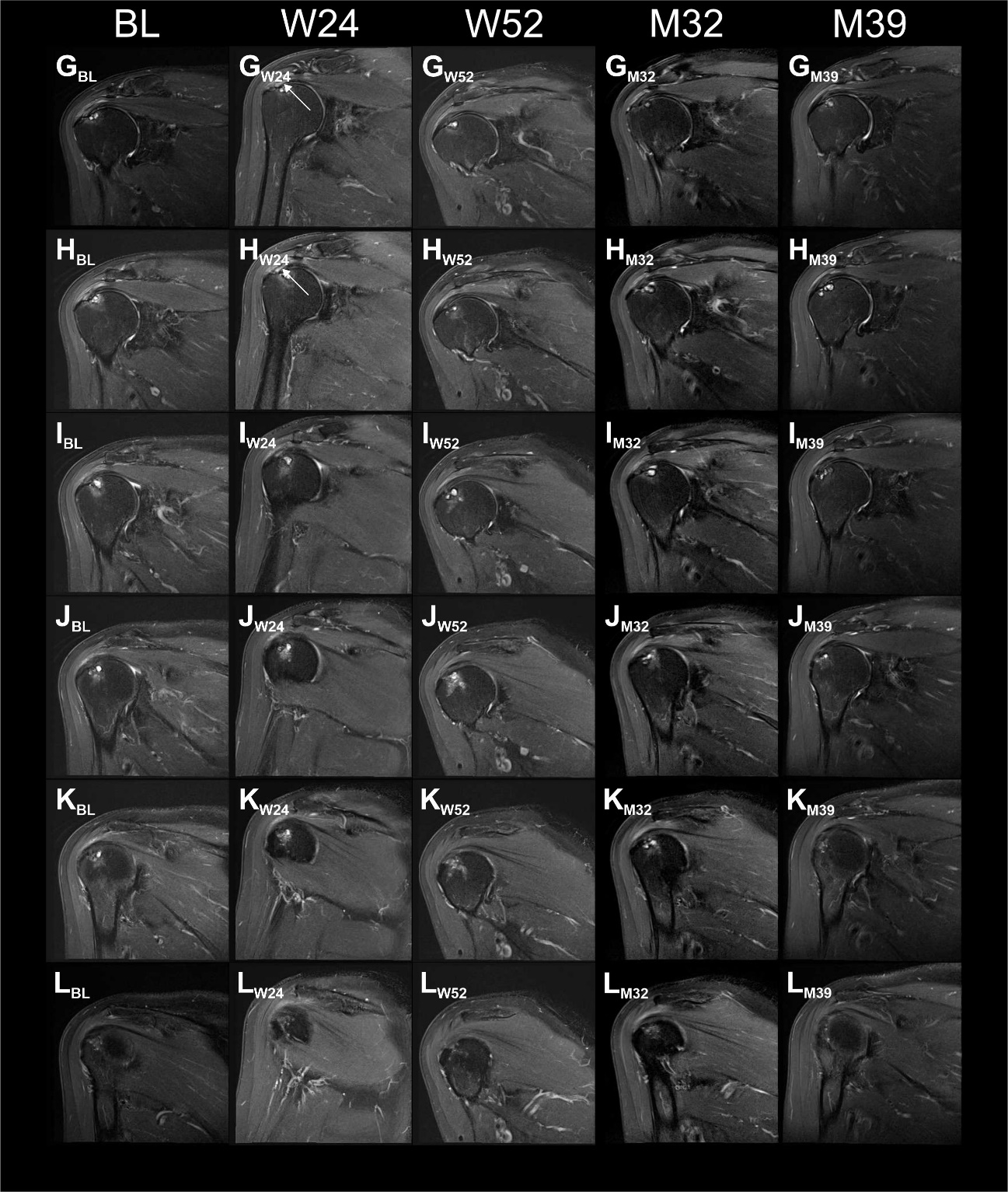


**Supplementary Figure S6.** Proton density weighted, fat saturated, T2-weighted, coronal magnetic resonance imaging (MRI) scans of the index shoulder of Subject A4 treated with injection of UA-ADRCs, generated during the present and the former studies. Panels A-L show the same (or nearly the same) image planes at different times, with Panels A showing the most ventral image plane and Panels L the most dorsal image plane. The arrows in Panels F_W24_, G_W24_, H_W24_ and I_W24_ indicate a hyperintense structure at the position of the supraspinatus tendon that was found at 24 weeks post-treatment but not at baseline. *BL* baseline, *W24 / W52* 24 / 52 weeks post-treatment, *M34* 34 months post-treatment (no MRI was performed during the second visit of Subject A3).


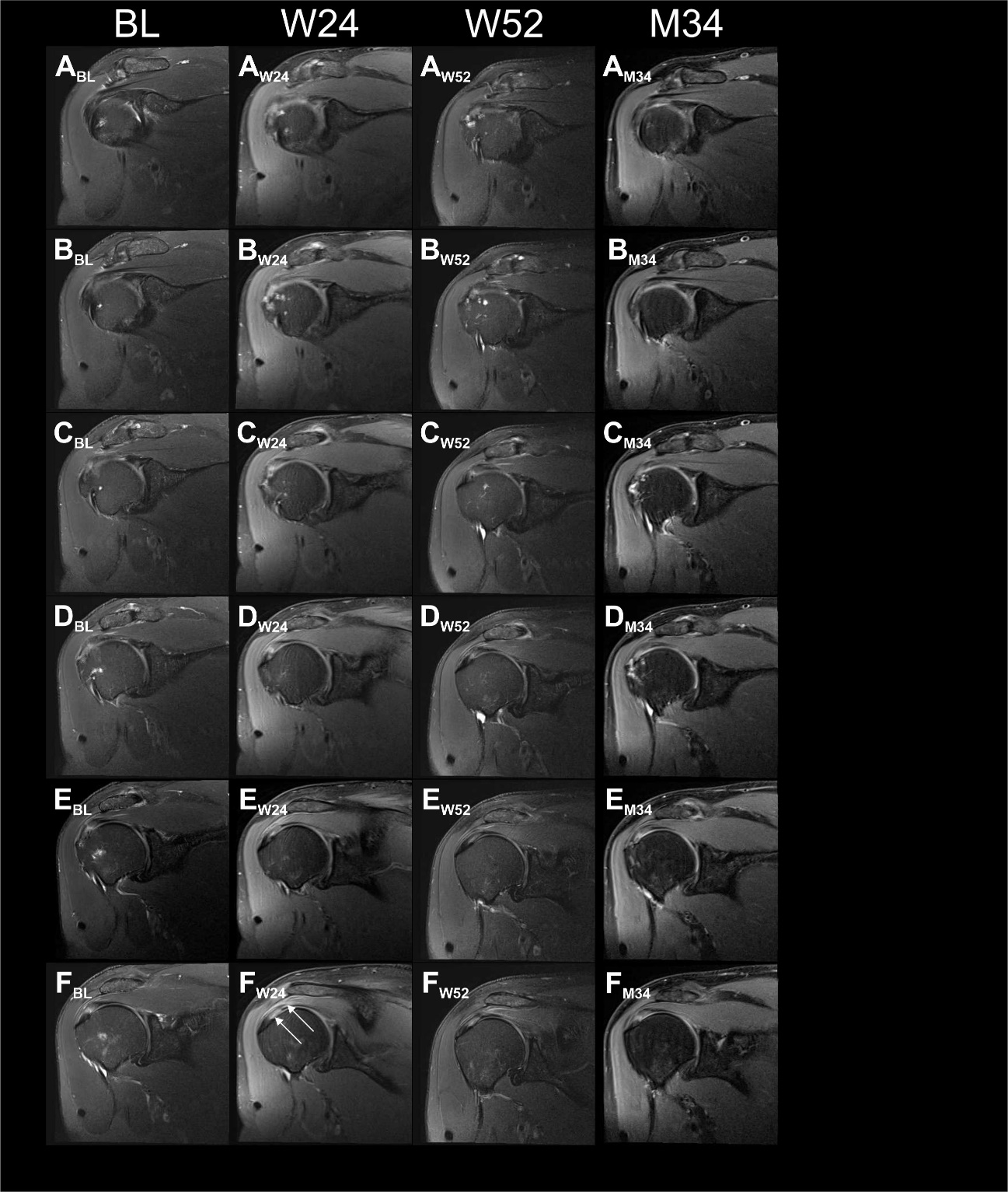


**Supplementary Figure S6 (cont.)**


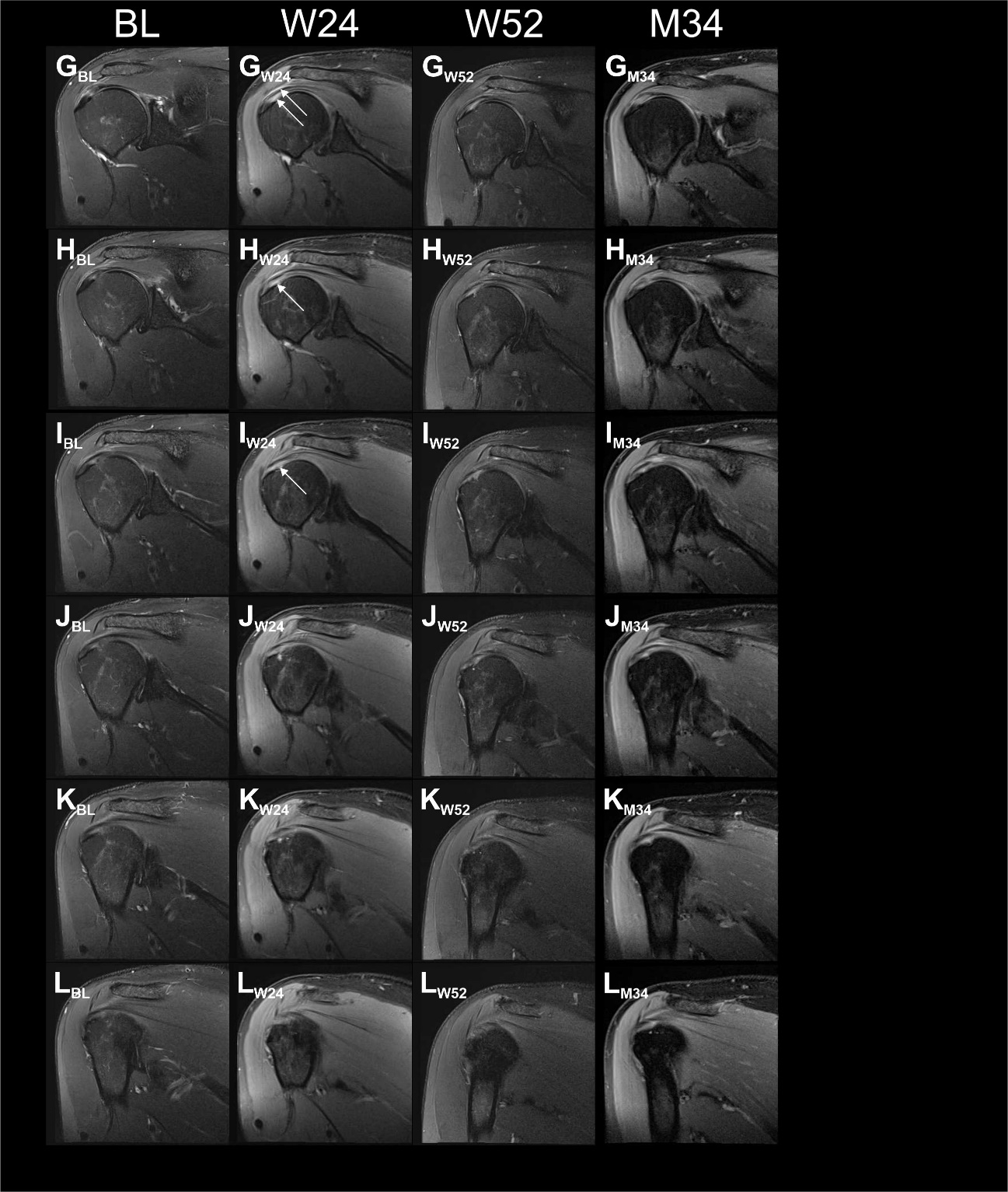


**Supplementary Figure S7.** Proton density weighted, fat saturated, T2-weighted, coronal magnetic resonance imaging (MRI) scans of the index shoulder of Subject A5 treated with injection of UA-ADRCs, generated during the present and the former studies. Panels A-L show the same (or nearly the same) image planes at different times, with Panels A showing the most ventral image plane and Panels L the most dorsal image plane. The arrows in Panels B_W24_, C_W24_, D_W24_, E_W24_, F_W24_, G_W24_ H_W24_, I_W24_ and J_W24_ indicate a hyperintense structure at the position of the supraspinatus tendon that was found at 24 weeks post-treatment but not at baseline. *BL* baseline, *W24 / W52* 24 / 52 weeks post-treatment, *M33 / M41* 33 / 41 months post-treatment.


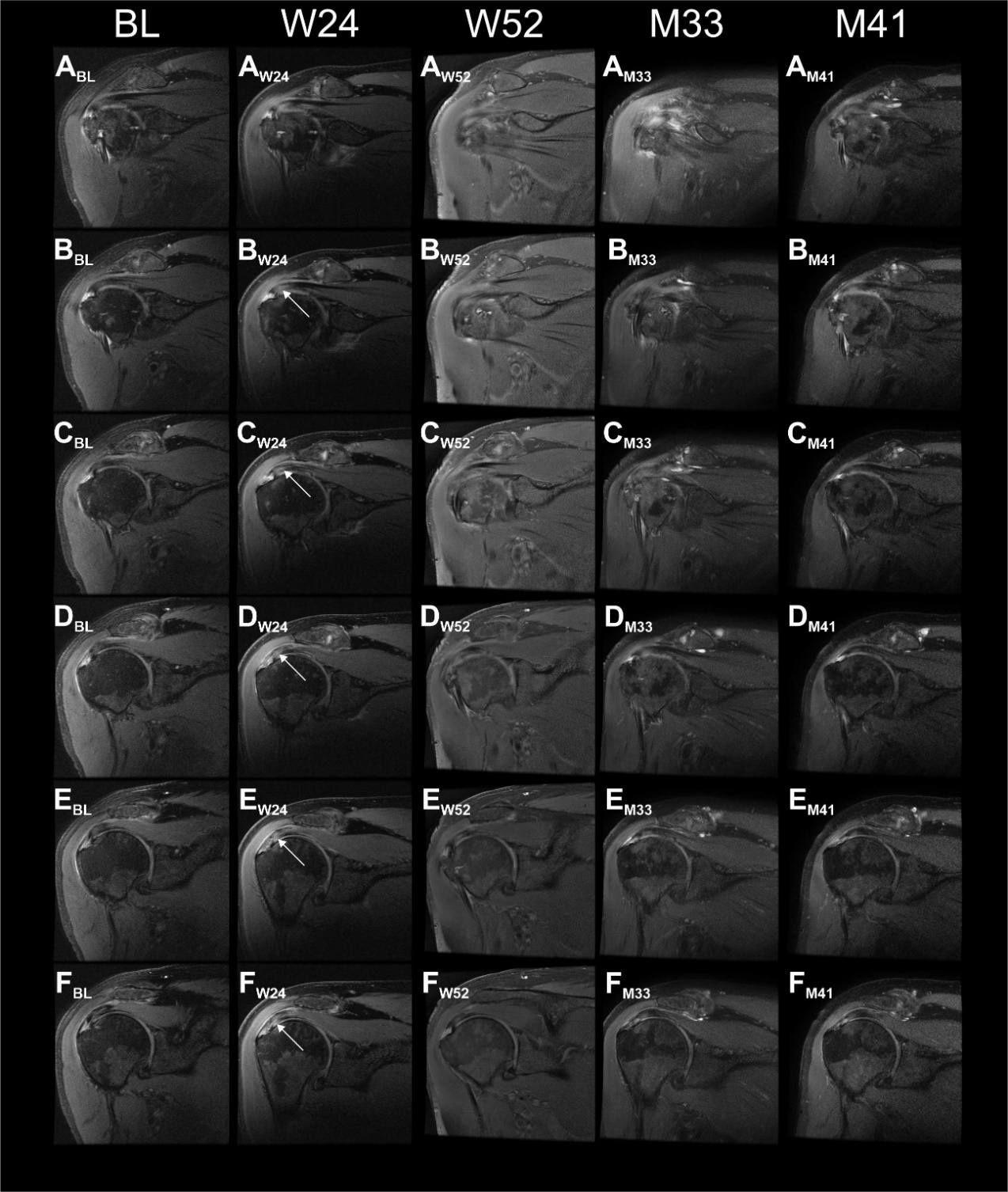


**Supplementary Figure S7 (cont.)**


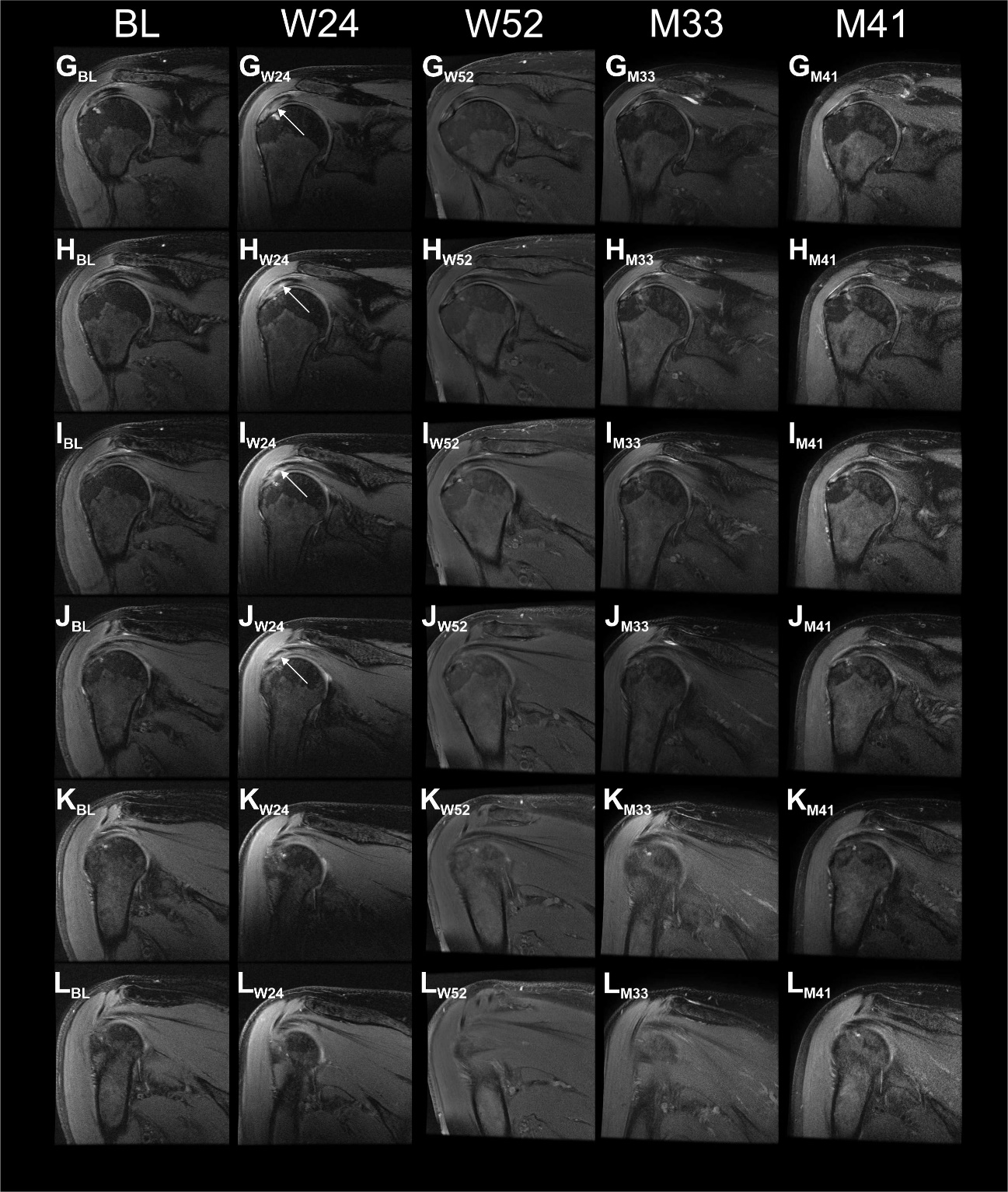


**Supplementary Figure S8.** Proton density weighted, fat saturated, T2-weighted, coronal magnetic resonance imaging (MRI) scans of the index shoulder of Subject A6 treated with injection of UA-ADRCs, generated during the present and the former studies. Panels A-L show the same (or nearly the same) image planes at different times, with Panels A showing the most ventral image plane and Panels L the most dorsal image plane. The arrows in Panels D_W24_, E_W24_ and F_W24_ indicate a hyperintense structure at the position of the supraspinatus tendon that was found at 24 weeks post-treatment but not at baseline. *BL* baseline, *W24 / W52* 24 / 52 weeks post-treatment, *M33 / M41* 33 / 41 months post-treatment.


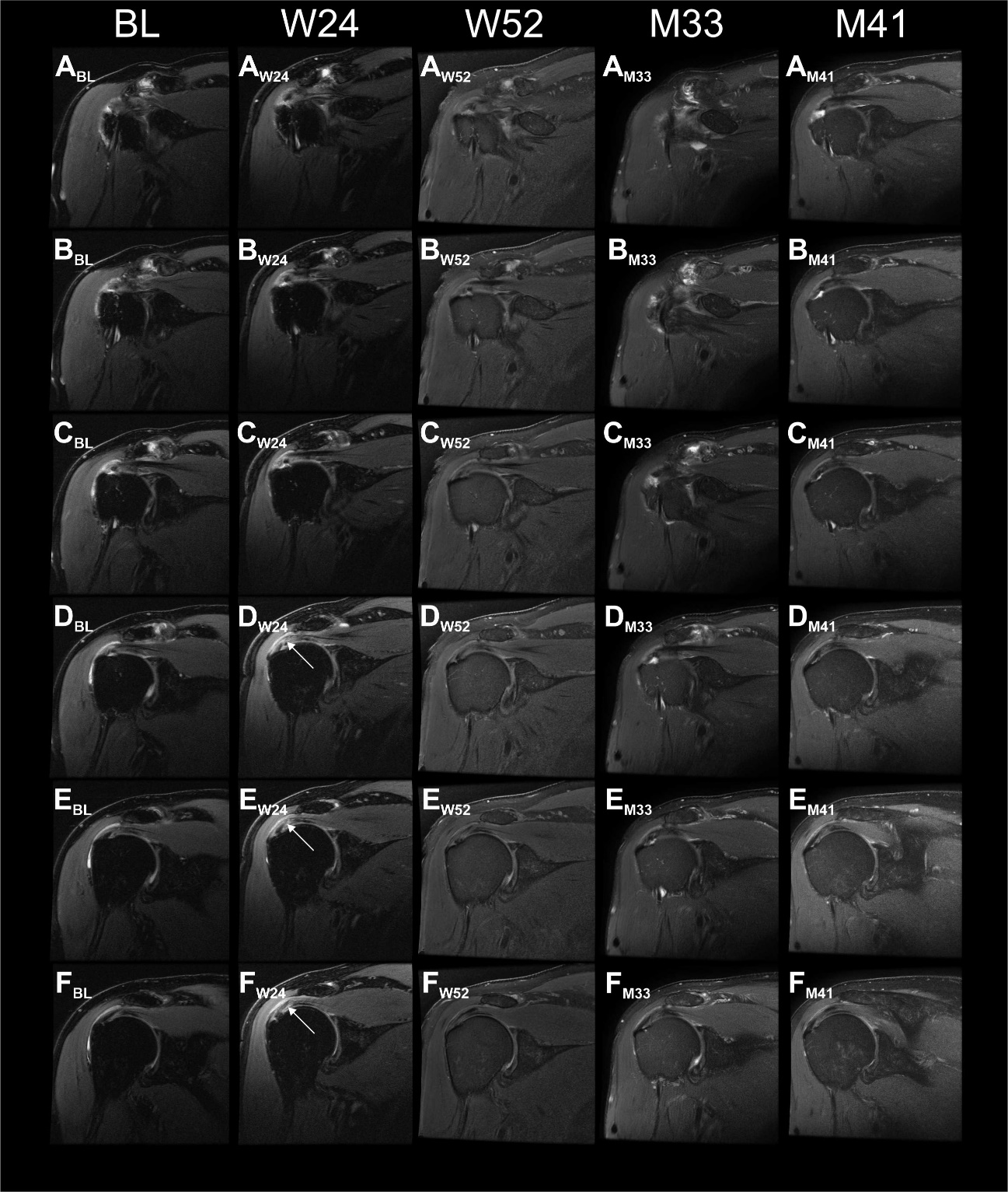


**Supplementary Figure S8 (cont.)**


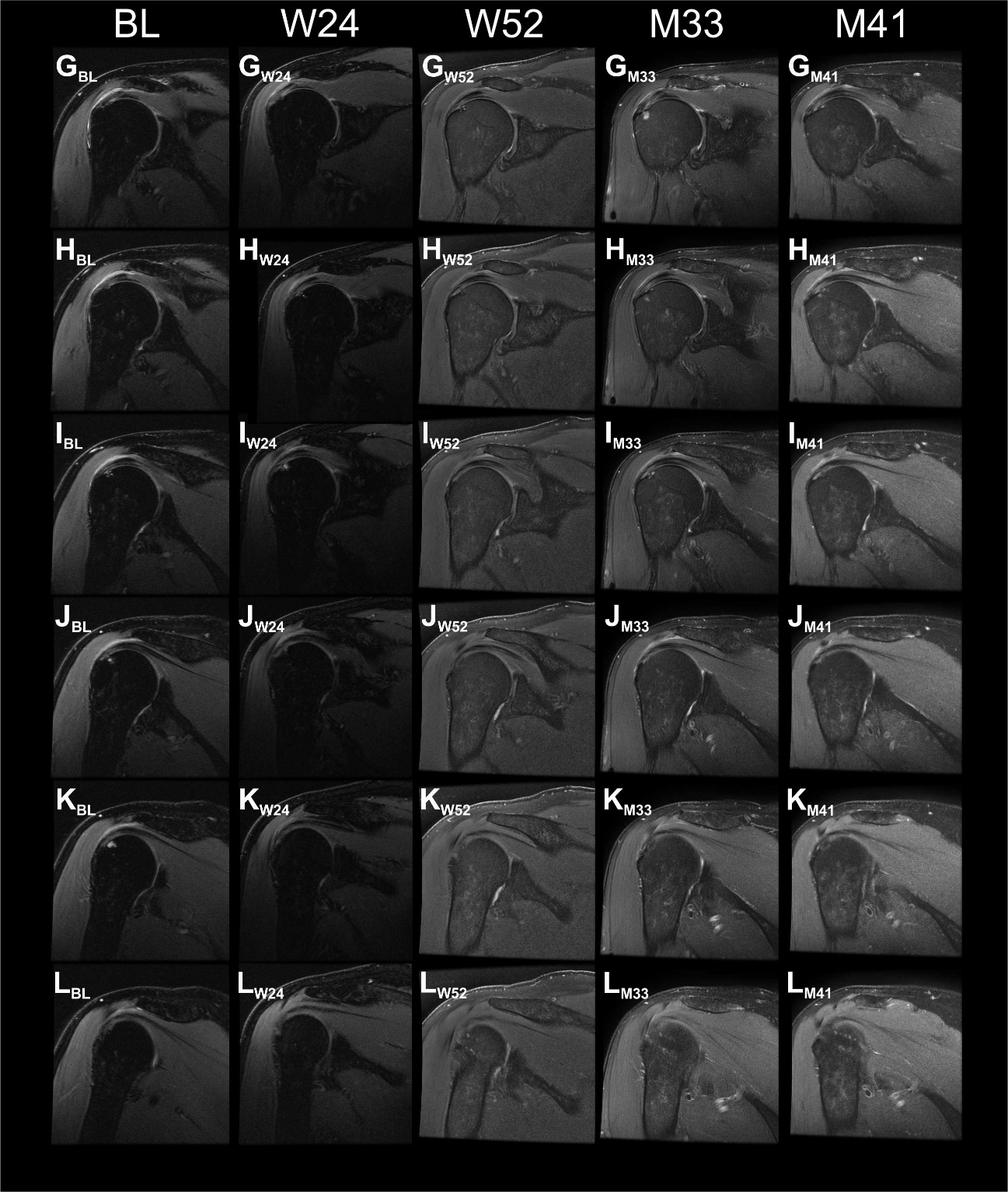


**Supplementary Figure S9.** Proton density weighted, fat saturated, T2-weighted, coronal magnetic resonance imaging (MRI) scans of the index shoulder of Subject A7 treated with injection of UA-ADRCs, generated during the present and the former studies. Panels A-L show the same (or nearly the same) image planes at different times, with Panels A showing the most ventral image plane and Panels L the most dorsal image plane. The arrows in Panels H_W24_ and I_W24_ indicate a hyperintense structure at the position of the supraspinatus tendon that was found at 24 weeks post-treatment but not at baseline. *BL* baseline, *W24 / W52* 24 / 52 weeks post-treatment, *M33 / M41* 33 / 41 months post-treatment.


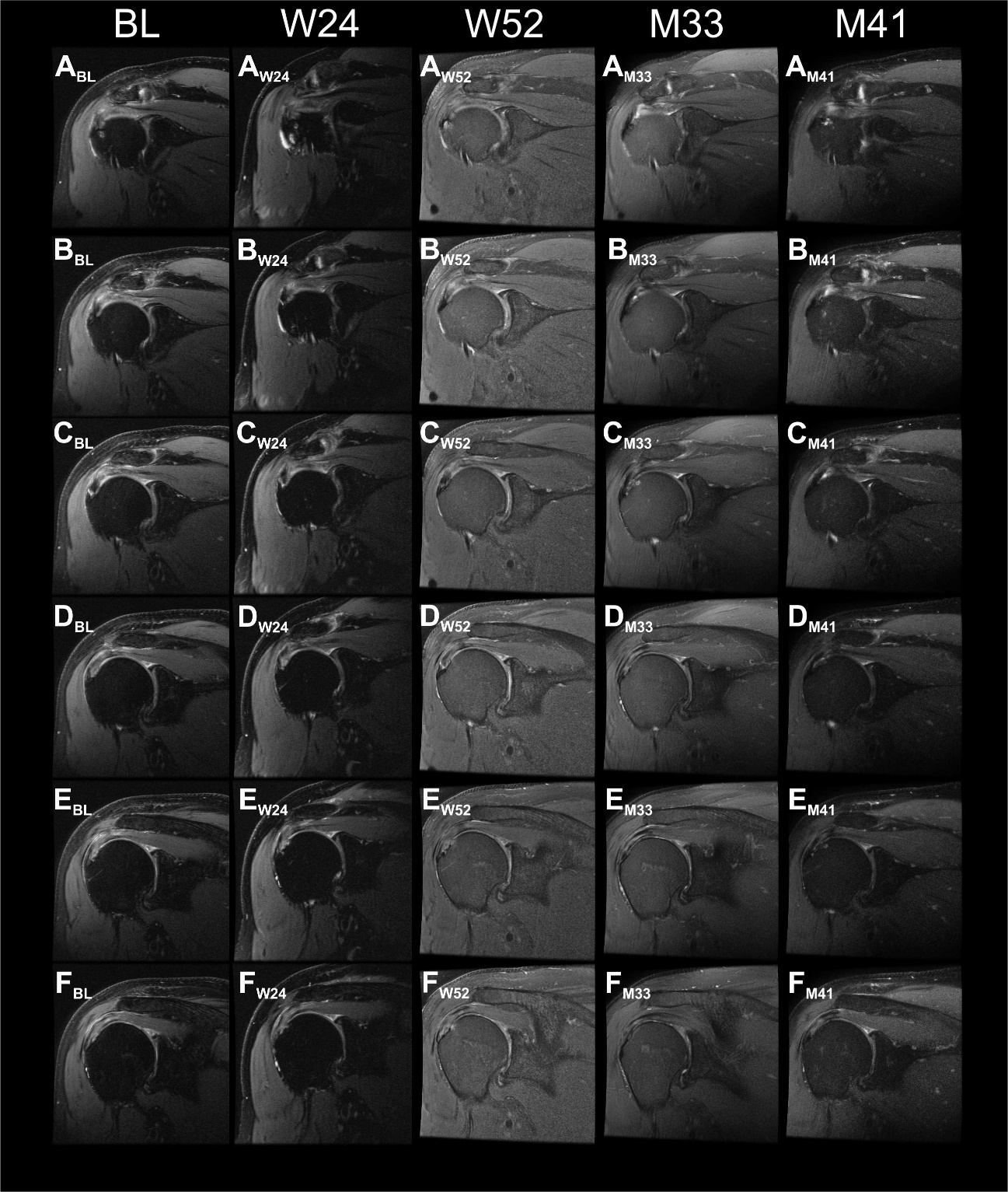


**Supplementary Figure S9 (cont.)**


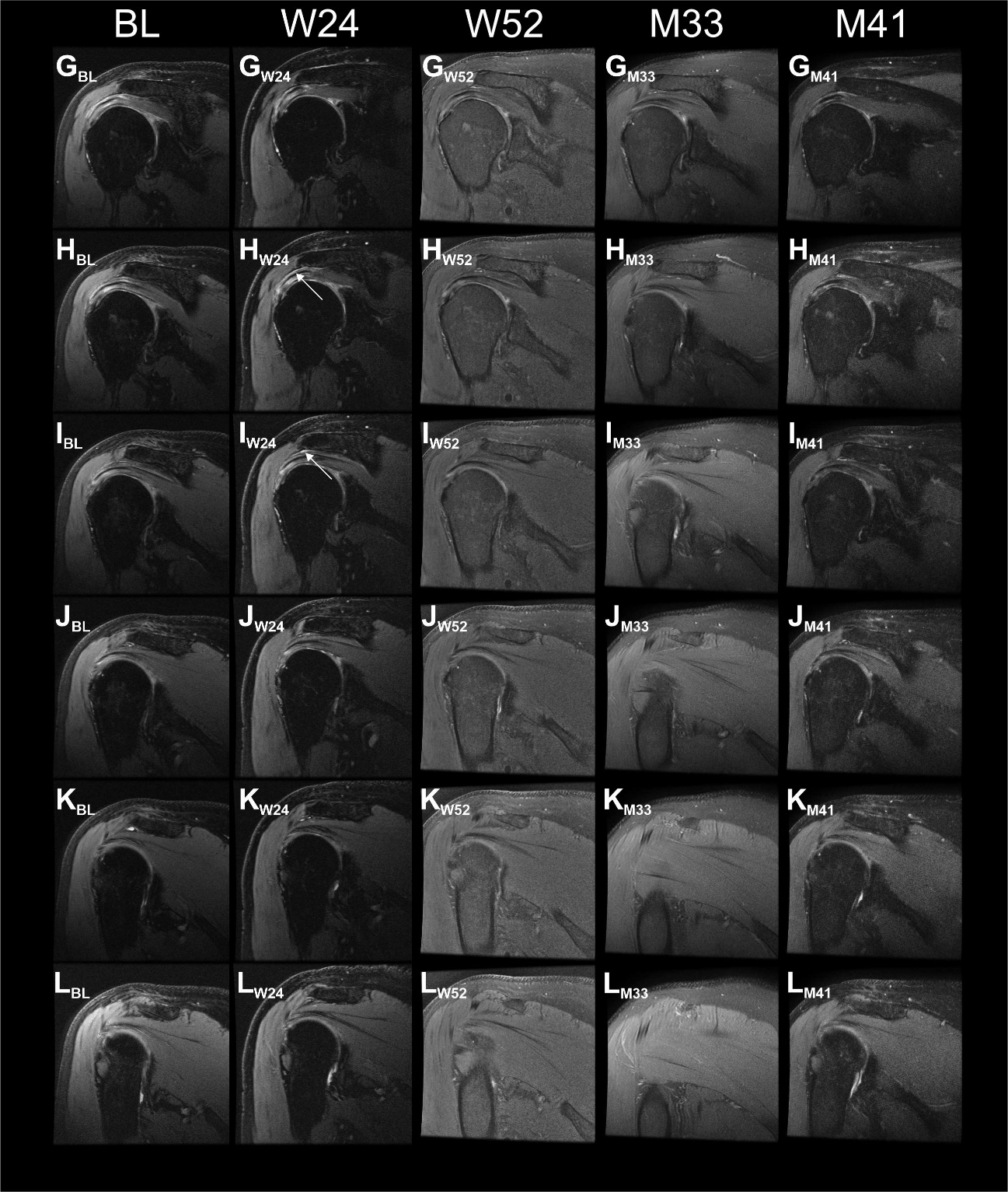


**Supplementary Figure S10.** Proton density weighted, fat saturated, T2-weighted, coronal magnetic resonance imaging (MRI) scans of the index shoulder of Subject A8 treated with injection of UA-ADRCs, generated during the present and the former studies. Panels A-L show the same (or nearly the same) image planes at different times, with Panels A showing the most ventral image plane and Panels L the most dorsal image plane. *BL* baseline, *W24 / W52* 24 / 52 weeks post-treatment, *M33 / M41* 31 / 39 months post-treatment.


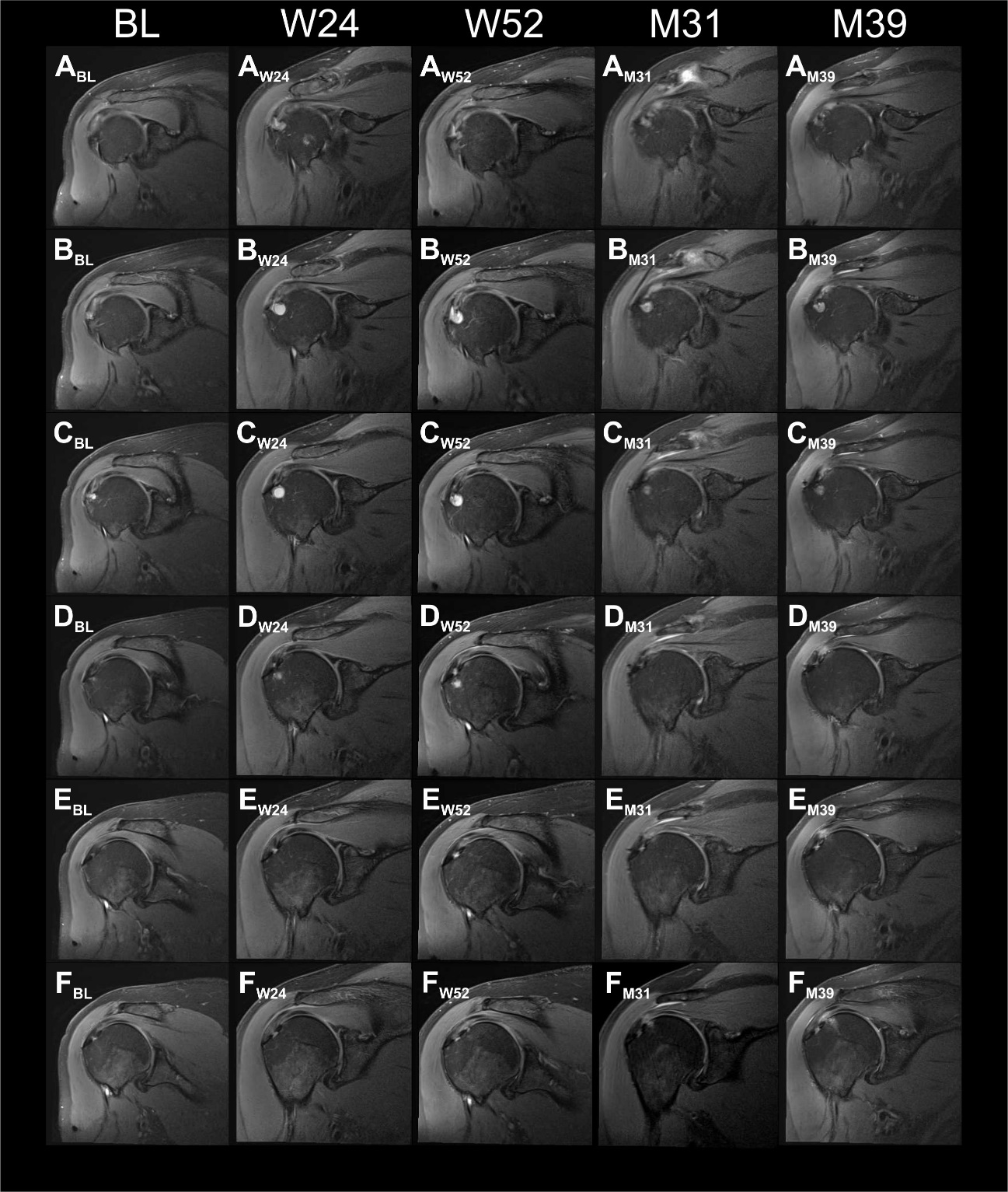


**Supplementary Figure S10 (cont.)**


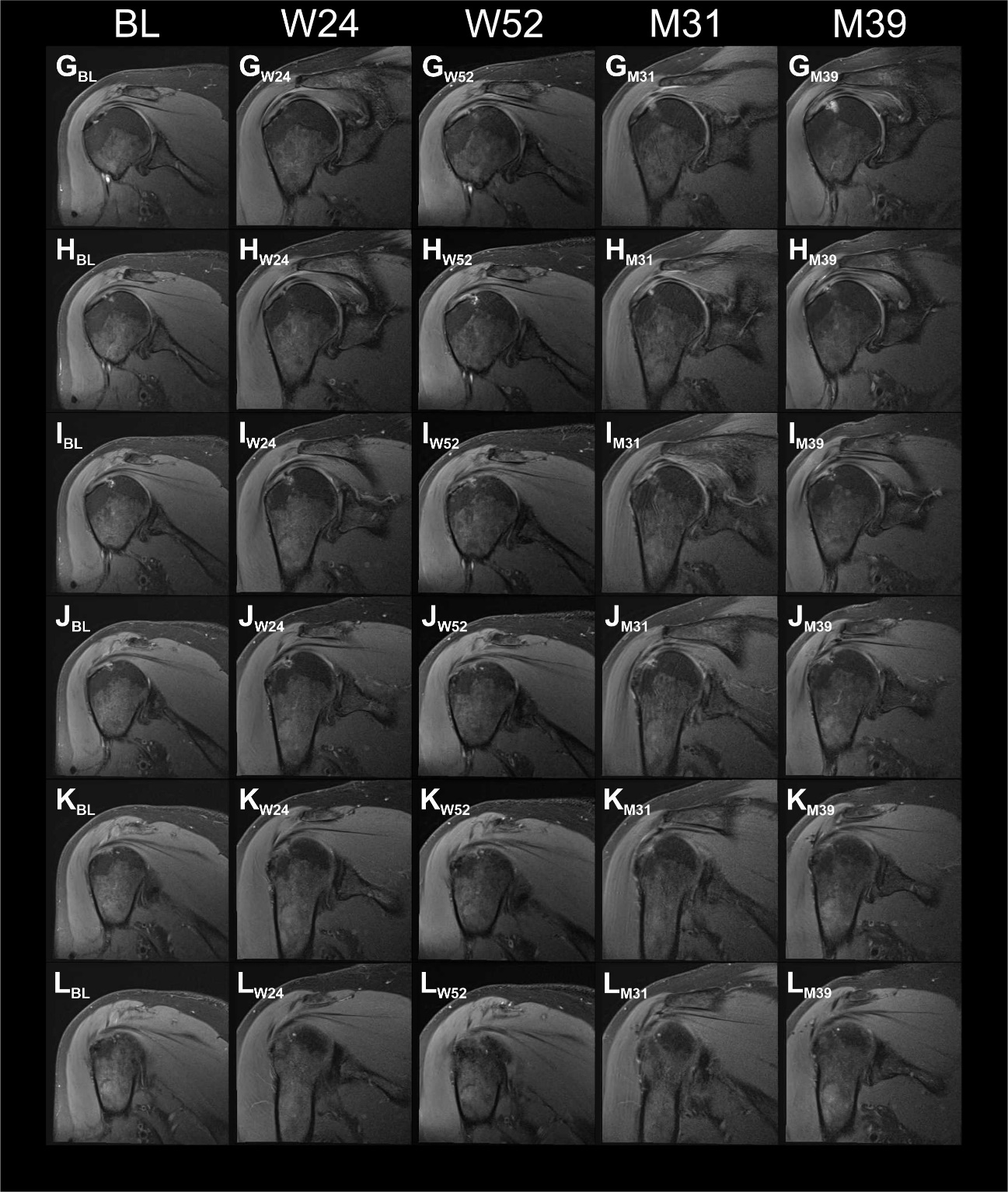


**Supplementary Figure S11.** Proton density weighted, fat saturated, T2-weighted, coronal magnetic resonance imaging (MRI) scans of the index shoulder of Subject A9 treated with injection of UA-ADRCs, generated during the present and the former studies. Panels A-L show the same (or nearly the same) image planes at different times, with Panels A showing the most ventral image plane and Panels L the most dorsal image plane. The arrows in Panels B_W24_, C_W24_ and D_W24_ indicate a hyperintense structure at the position of the supraspinatus tendon that was found at 24 weeks post-treatment but not at baseline. *BL* baseline, *W24 / W52* 24 / 52 weeks post-treatment, *M33* 33 months post-treatment (no MRI was performed during the second visit of Subject A9).


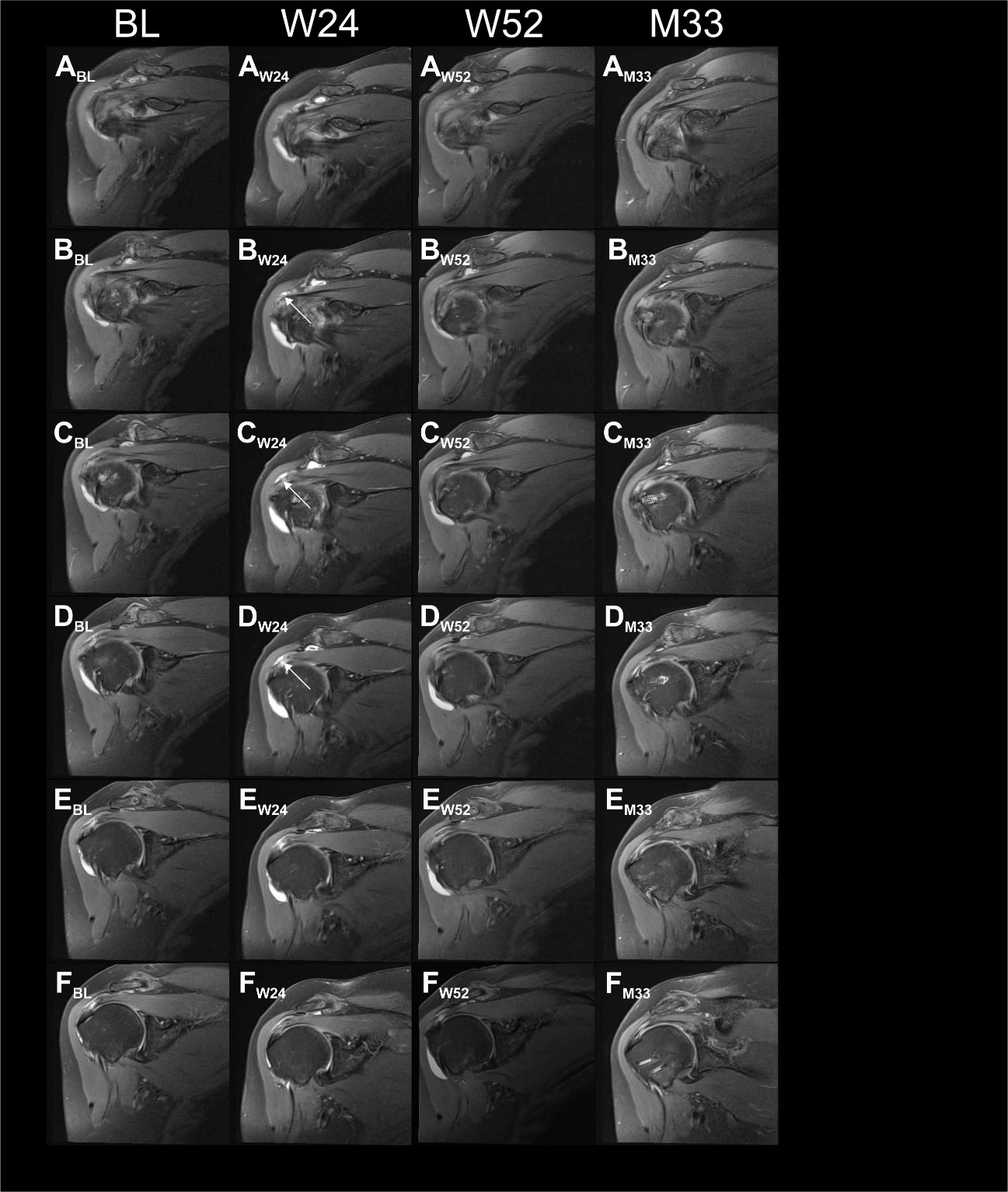


**Supplementary Figure S11 (cont.)**


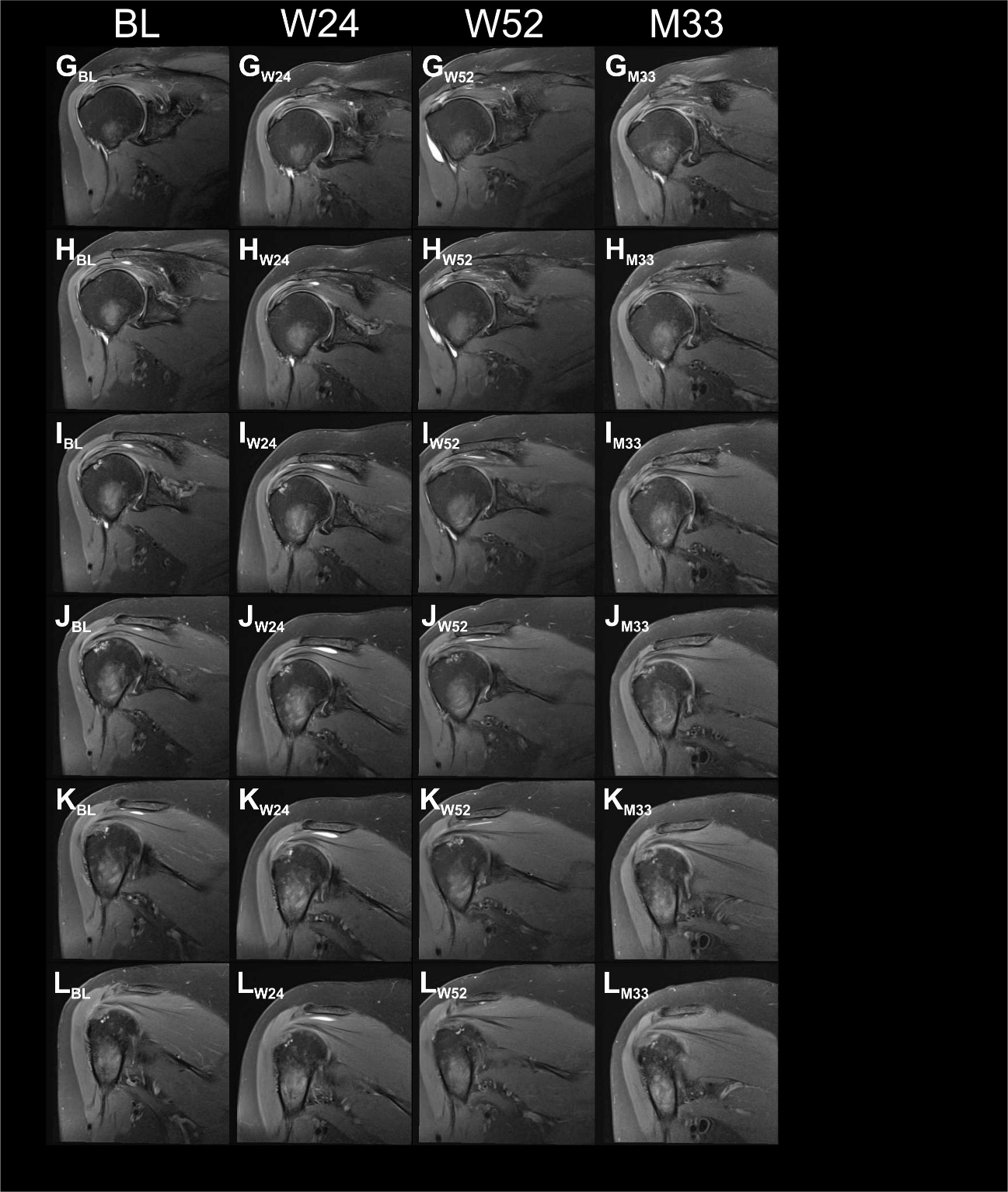


**Supplementary Figure S12.** Proton density weighted, fat saturated, T2-weighted, coronal magnetic resonance imaging (MRI) scans of the index shoulder of Subject A10 treated with injection of UA-ADRCs, generated during the present and the former studies. Panels A-L show the same (or nearly the same) image planes at different times, with Panels A showing the most ventral image plane and Panels L the most dorsal image plane. The arrows in Panels C_W24_ and D_W24_ indicate a hyperintense structure at the position of the supraspinatus tendon that was found at 24 weeks post-treatment but not at baseline. *BL* baseline, *W24 / W52* 24 / 52 weeks post-treatment, *M35 / M41* 35 / 41 months post-treatment.


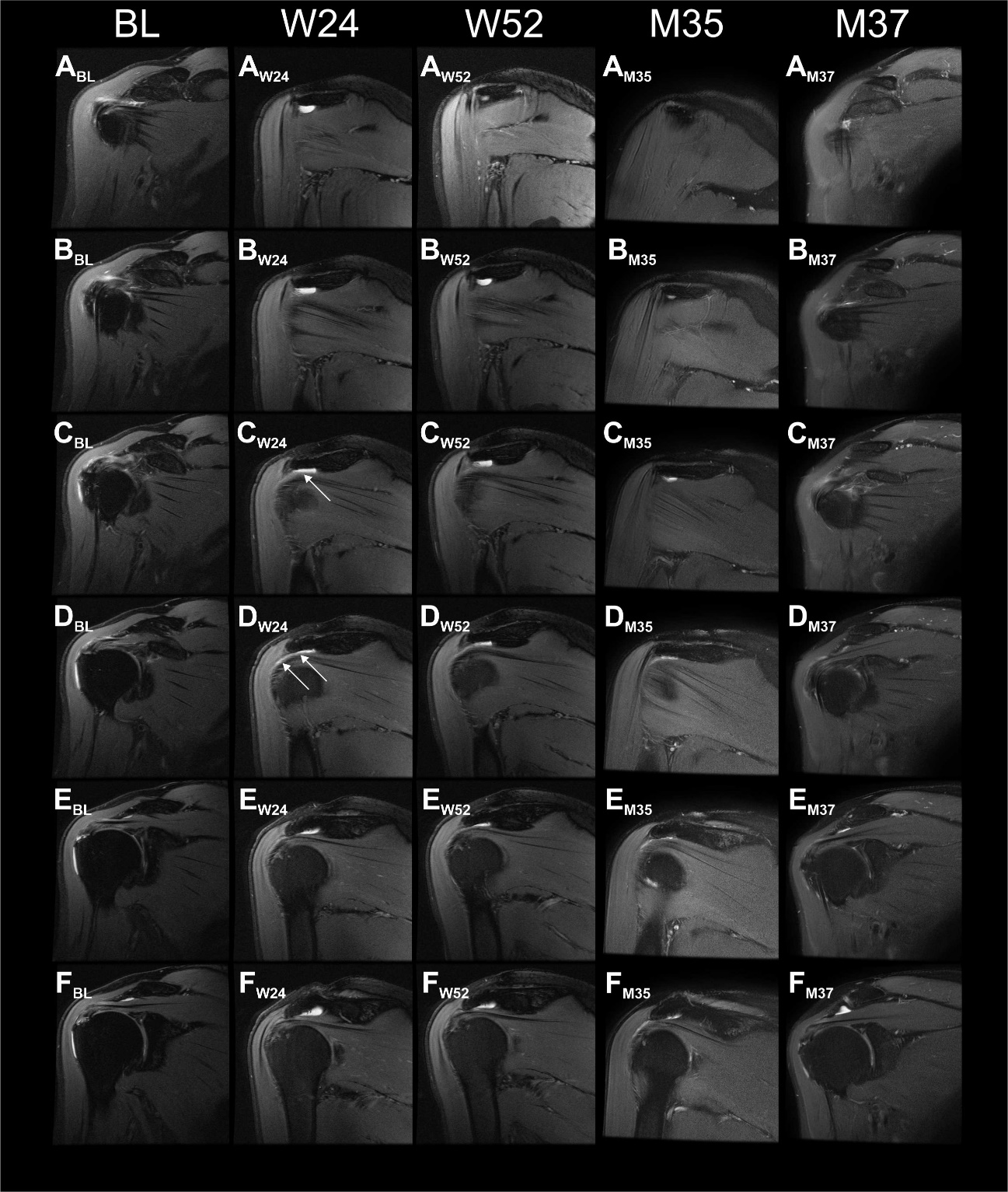


**Supplementary Figure S12 (cont.)**


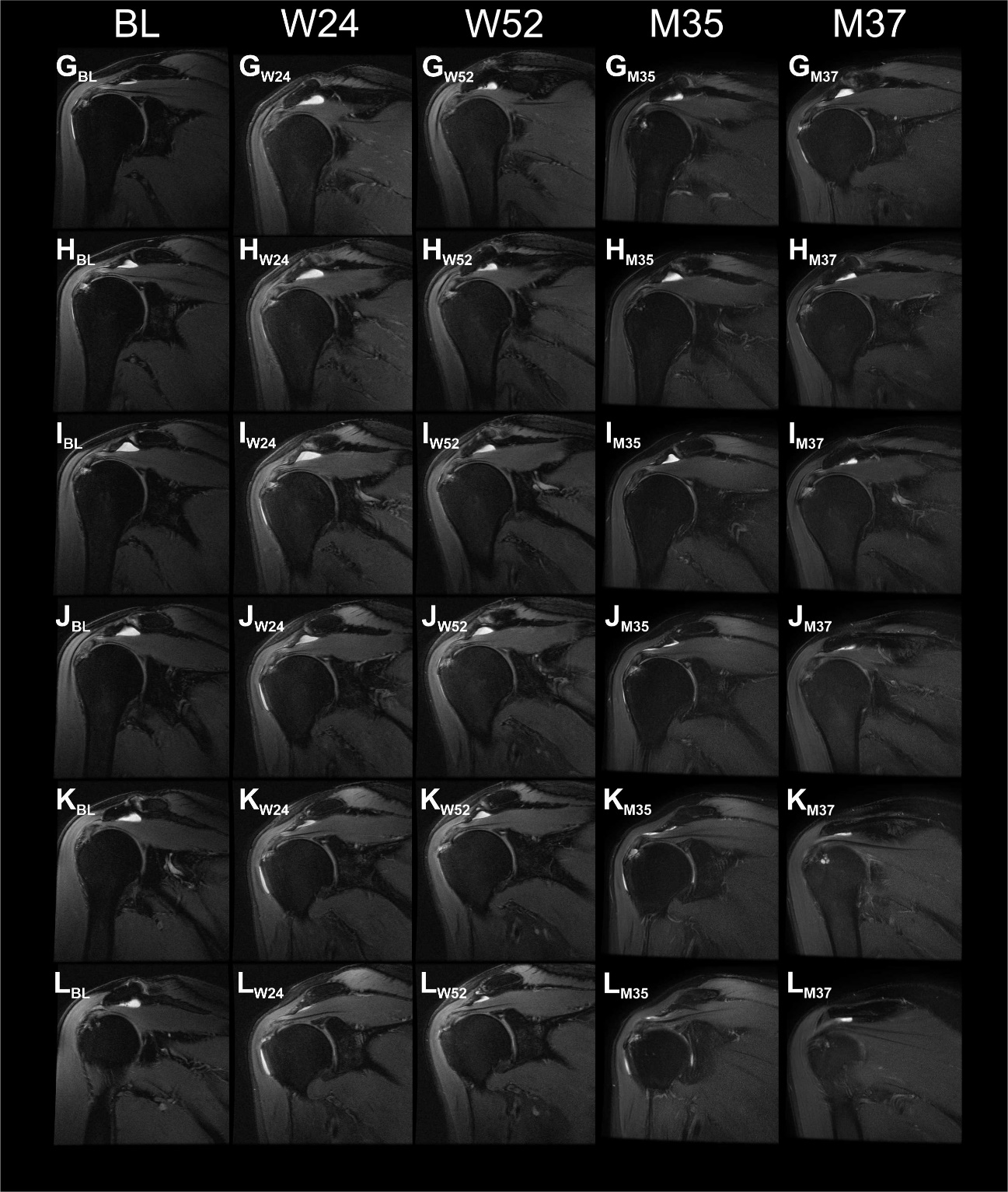


**Supplementary Figure S13.** Proton density weighted, fat saturated, T2-weighted, coronal magnetic resonance imaging (MRI) scans of the index shoulder of Subject A11 treated with injection of UA-ADRCs, generated during the present and the former studies. Panels A-L show the same (or nearly the same) image planes at different times, with Panels A showing the most ventral image plane and Panels L the most dorsal image plane.The arrows in Panels C_W24_, D_W24_, H_W24_, I_W24_, J_W24_, K_W24_ and L_W24_ indicate a hyperintense structure at the position of the supraspinatus tendon that was found at 24 weeks post-treatment but not at baseline. *BL* baseline, *W24 / W52* 24 / 52 weeks post-treatment, *M33* 33 months post-treatment (note that no MRI was performed during the second visit of Subject A11).


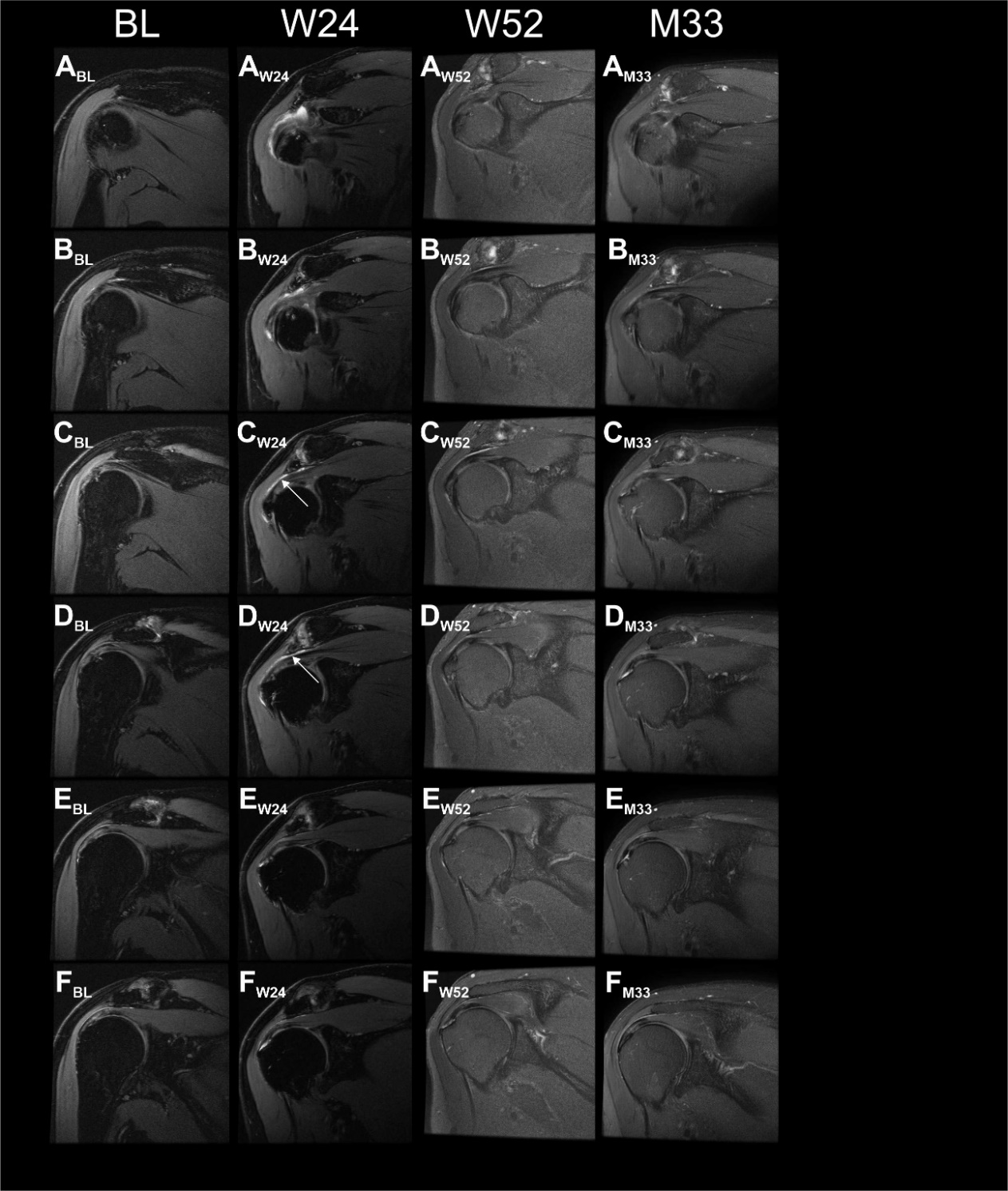


**Supplementary Figure S13 (cont.)**


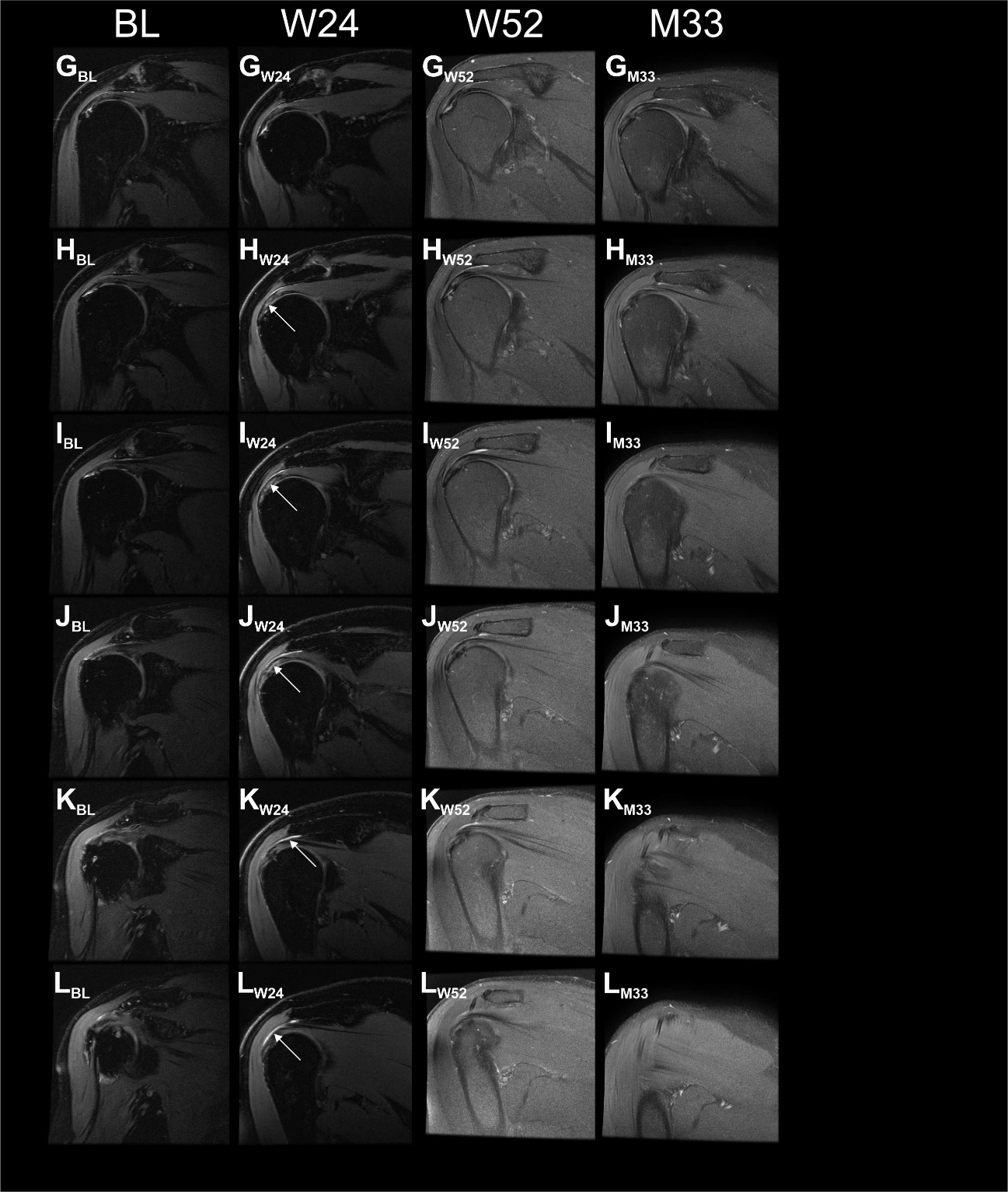


**Supplementary Figure S14.** Proton density weighted, fat saturated, T2-weighted, coronal magnetic resonance imaging (MRI) scans of the index shoulder of Subject C1 treated with injection of corticosteroid, generated during the present and the former studies. Panels A-L show the same (or nearly the same) image planes at different times, with Panels A showing the most ventral image plane and Panels L the most dorsal image plane. *BL* baseline, *W24 / W52* 24 / 52 weeks post-treatment, *M33 / M41* 33 / 41 months post-treatment.


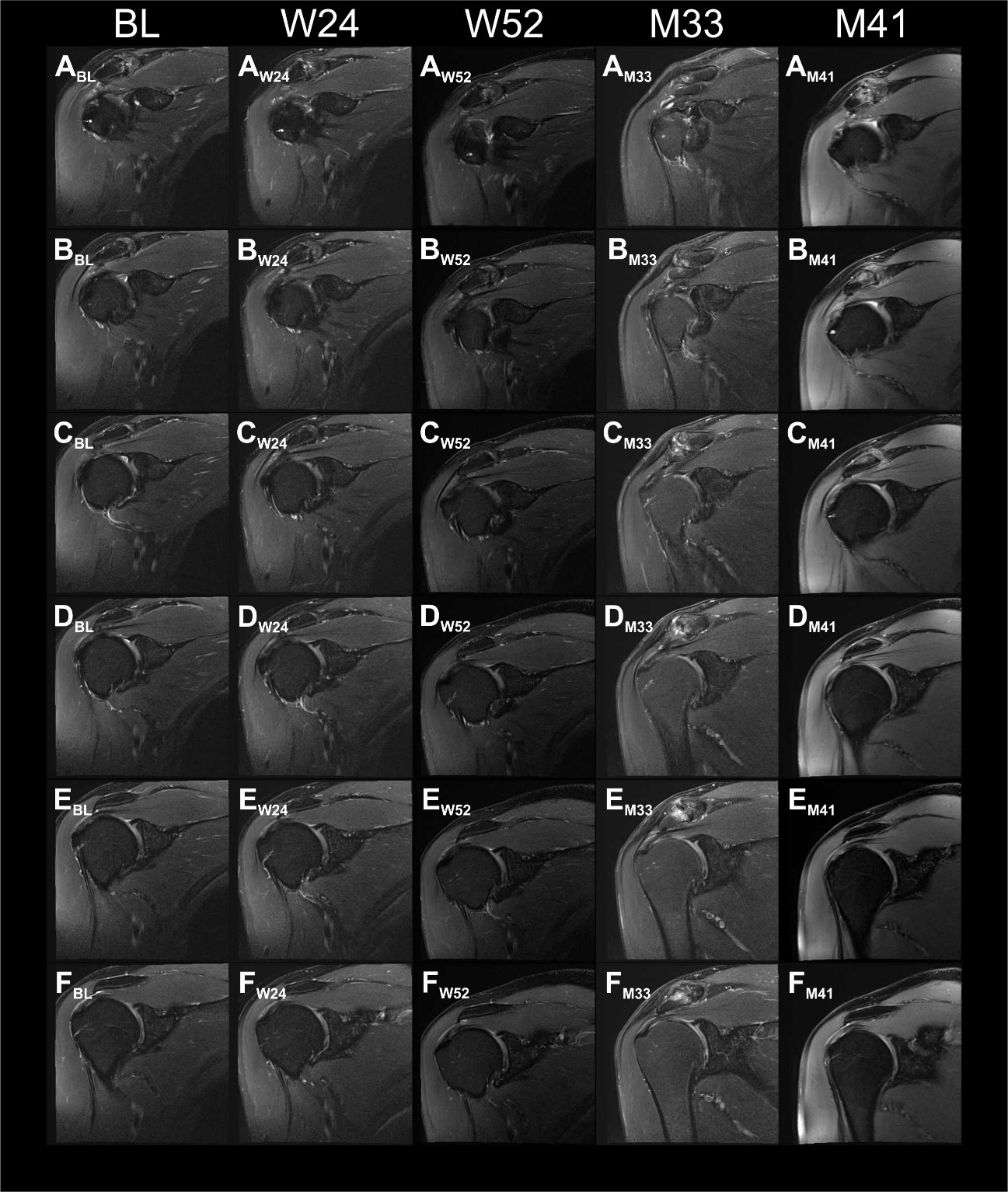


**Supplementary Figure S14 (cont.)**


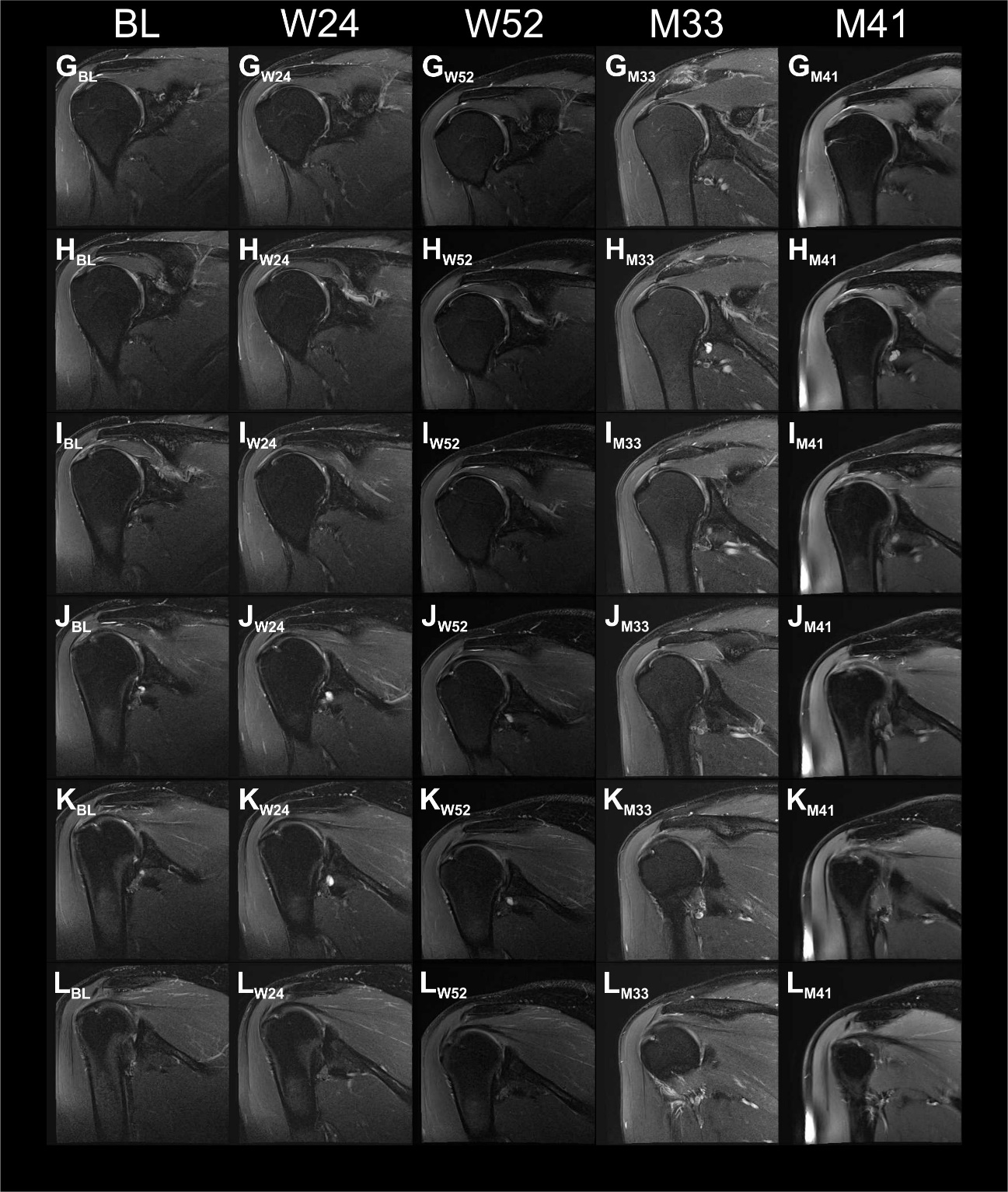


**Supplementary Figure S15.** Proton density weighted, fat saturated, T2-weighted, coronal magnetic resonance imaging (MRI) scans of the index shoulder of Subject C2 treated with injection of corticosteroid, generated during the present and the former studies. Panels A-L show the same (or nearly the same) image planes at different times, with Panels A showing the most ventral image plane and Panels L the most dorsal image plane. *BL* baseline, *W24 / W52* 24 / 52 weeks post-treatment, *M34 / M42* 34 / 42 months post-treatment.


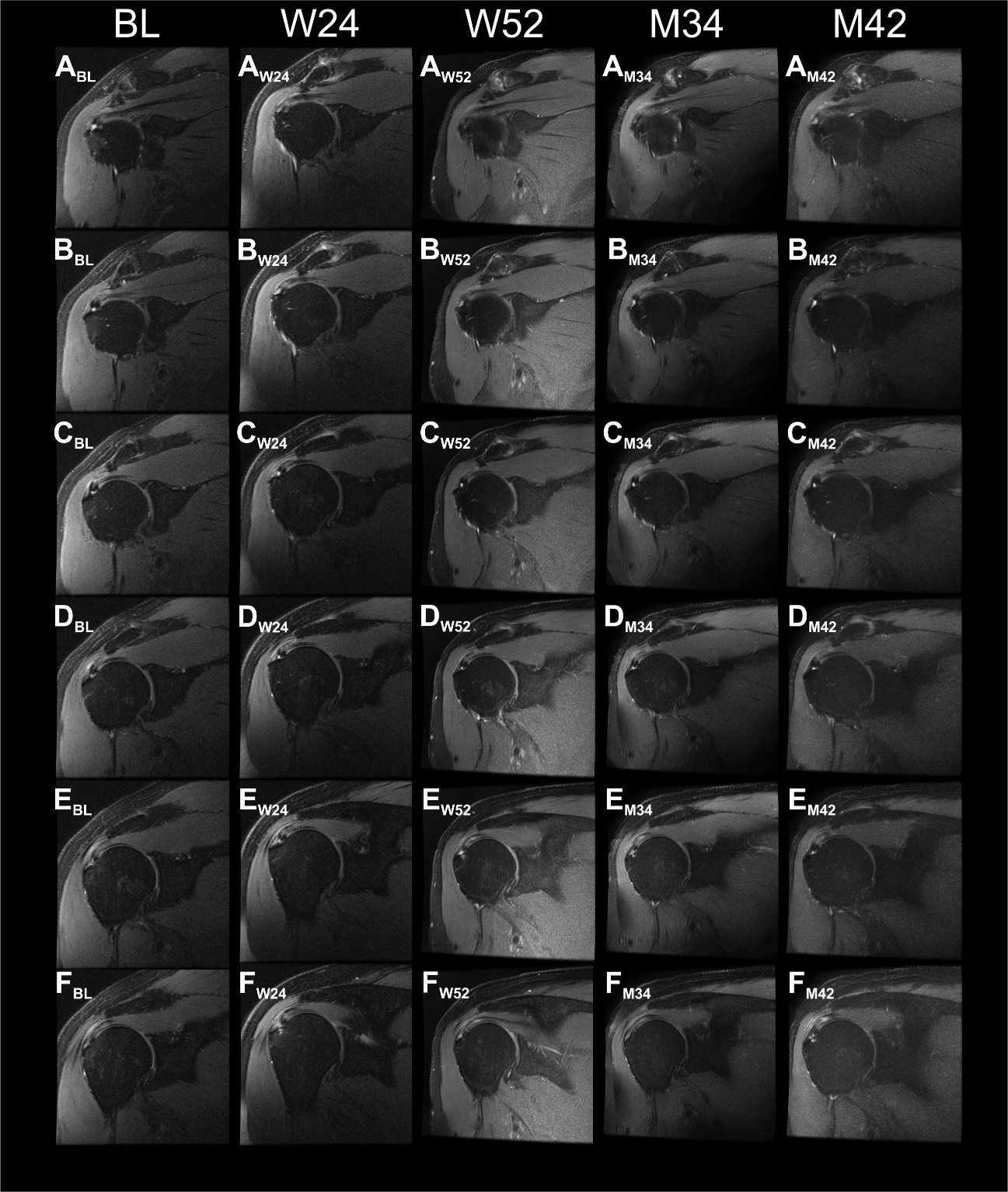


**Supplementary Figure S15 (cont.)**


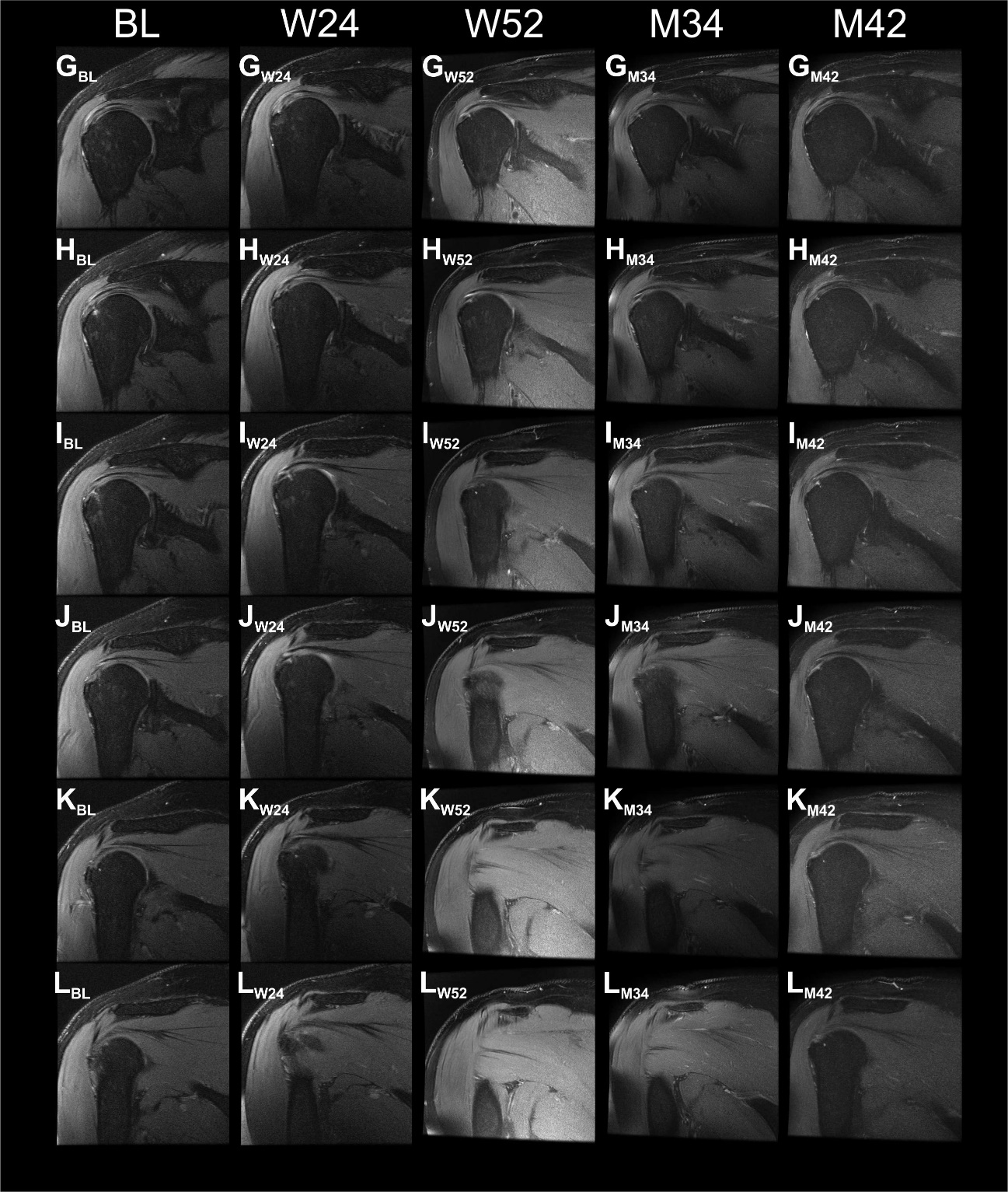


**Supplementary Figure S16.** Proton density weighted, fat saturated, T2-weighted, coronal magnetic resonance imaging (MRI) scans of the index shoulder of Subject C3 treated with injection of corticosteroid, generated during the present and the former studies.

Panels A-L show the same (or nearly the same) image planes at different times, with Panels A showing the most ventral image plane and Panels L the most dorsal image plane.

Abbreviations: BL, baseline; W24 / W52, 24 / 52 weeks post-treatment; M33 / M41, 33 / 41 months post-treatment.


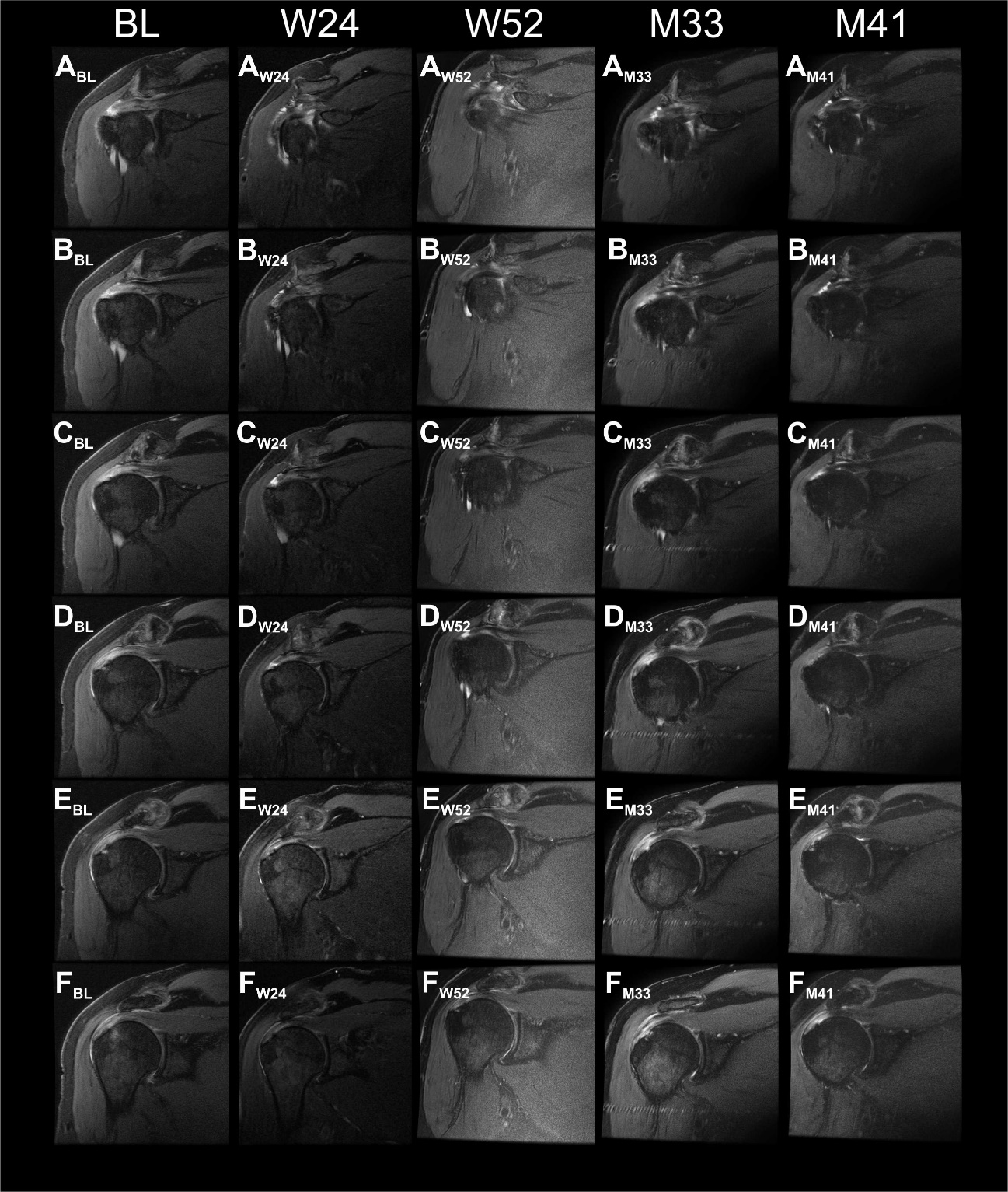


**Supplementary Figure S16 (cont.)**


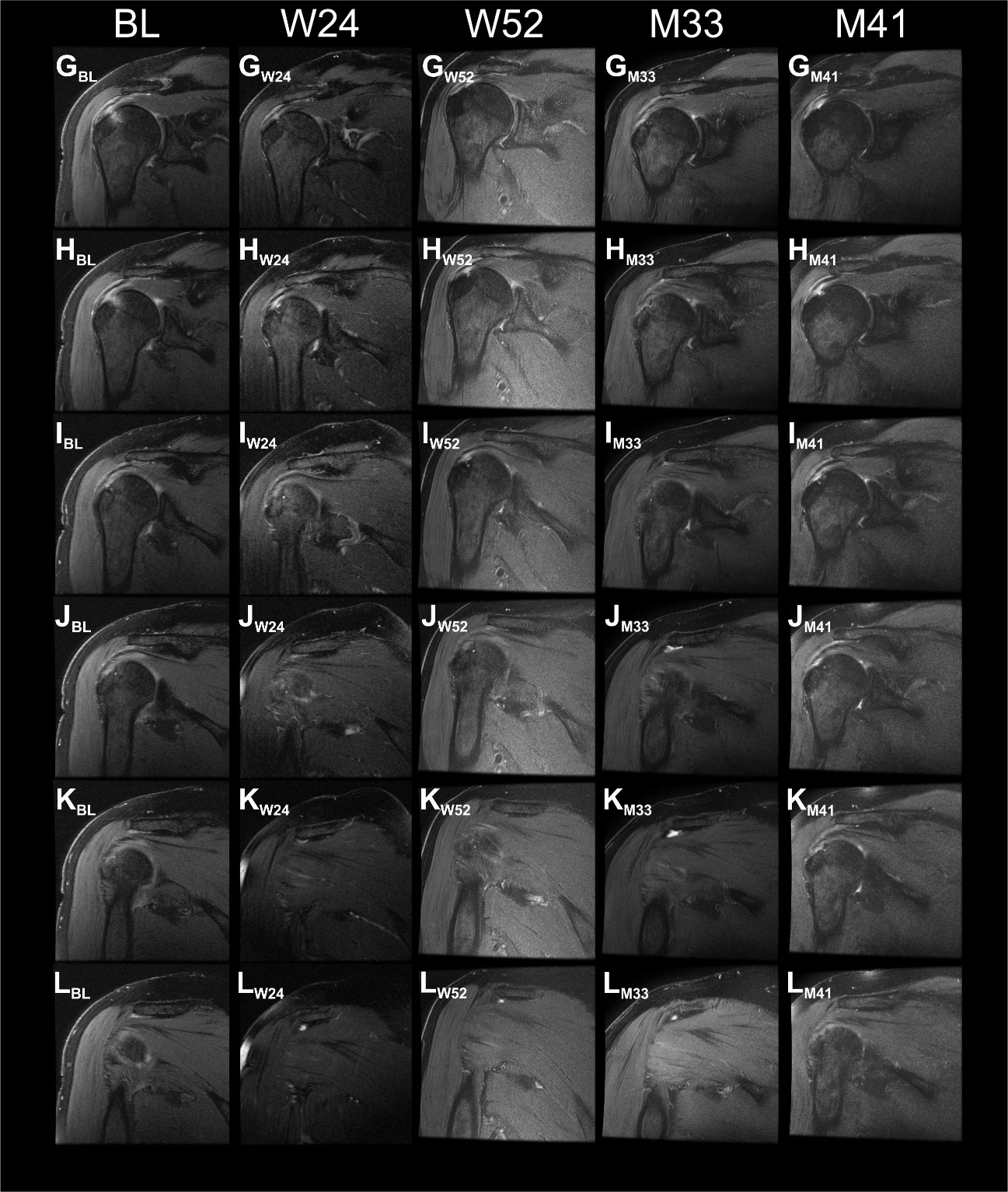


**Supplementary Figure S17.** Proton density weighted, fat saturated, T2-weighted, coronal magnetic resonance imaging (MRI) scans of the index shoulder of Subject C4 treated with injection of corticosteroid, generated during the present and the former studies. Panels A-L show the same (or nearly the same) image planes at different times, with Panels A showing the most ventral image plane and Panels L the most dorsal image plane. *BL* baseline, *W24 / W52* 24 / 52 weeks post-treatment, *M35 / M40* 35 / 40 months post-treatment.


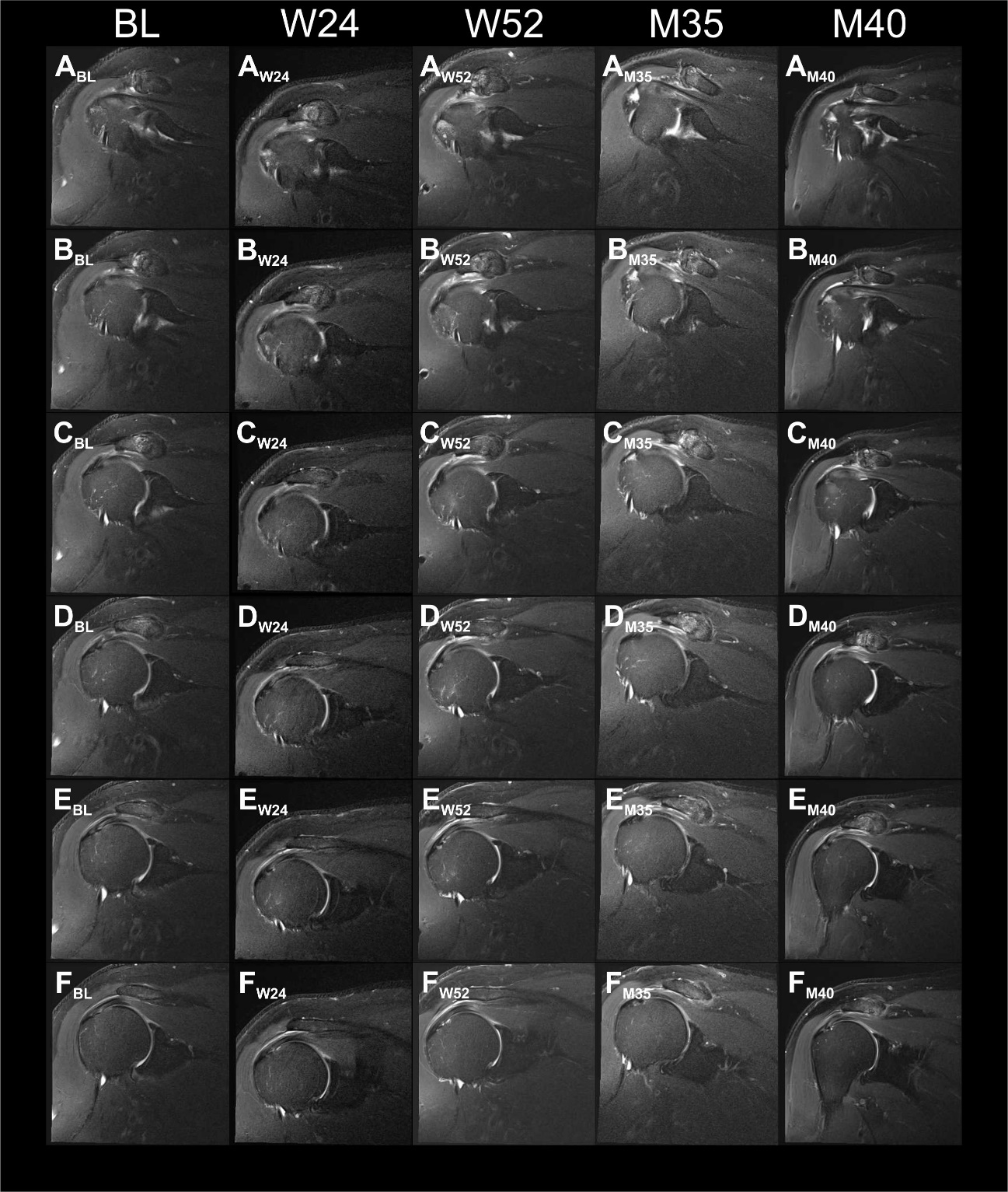


**Supplementary Figure S17 (cont.)**


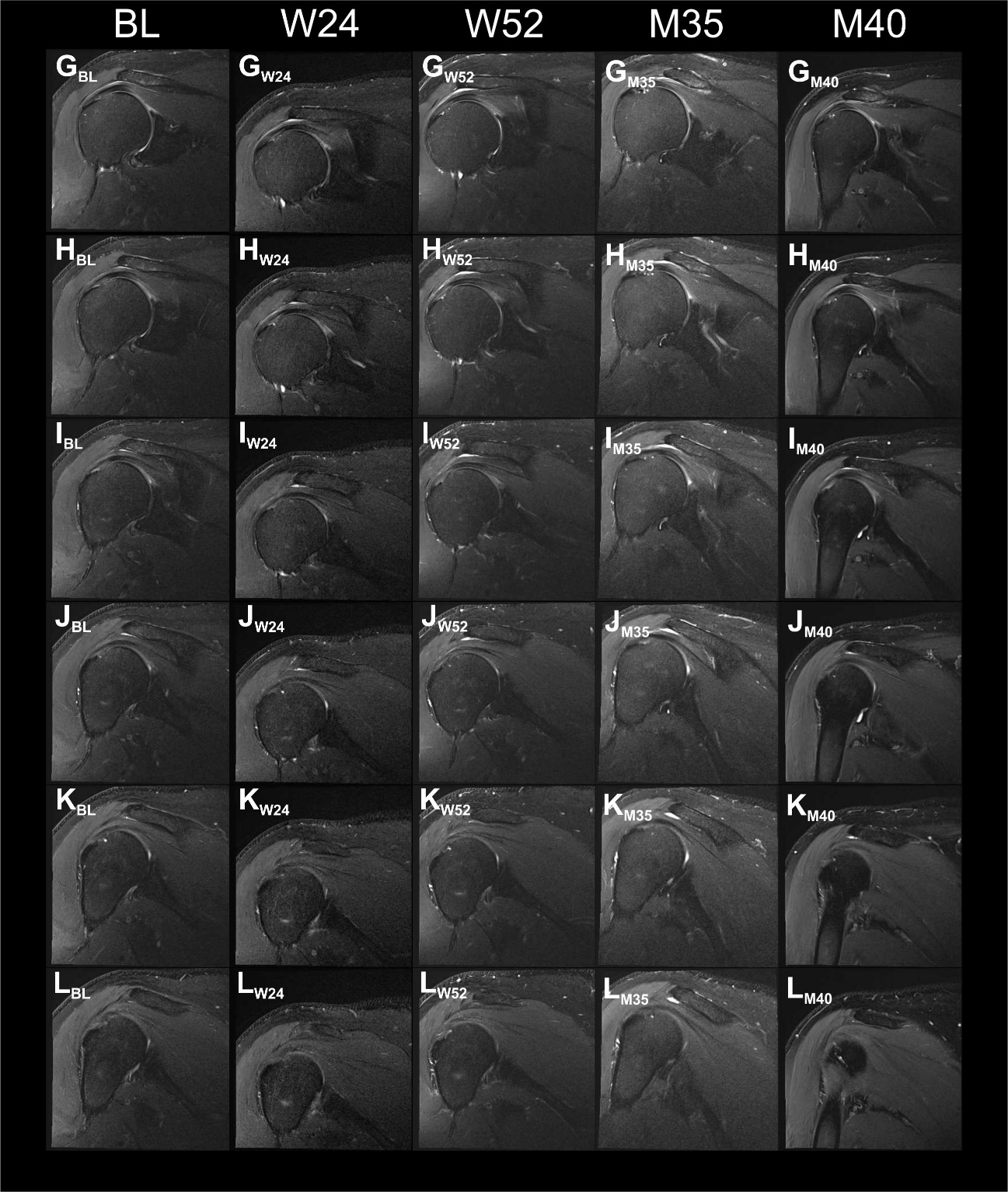


**Part 3 – Treatment-emergent adverse events that occurred during the present study and the former study**

**2.1. Details of all treatment-emergent adverse events** **that occurred during the present study and the former study**

Supplementary Tables S1 and S2 provide details of all treatment emergent adverse events (TEAEs) reported in the present study and the former study, stratified by the relation to the investigated treatment, severity and month post-treatment during which the TEAEs occurred.

**Supplementary Table S1.** Treatment-emergent adverse events reported by those subjects who were treated with injection of UA-ADRCs. Adverse events related to the index shoulder are given boldface. *UA-ADRCs* fresh, uncultured, unmodified, autologous, adipose-derived regenerative cells, *M* month post-treatment when the treatment-emergent adverse event started, *F* former study, *P* present study, *R* relation to the investigated treatment, *^a^* outlined in detail in Supplementary Table S8, *^b^* outlined in detail in Supplementary Table S13, *^c^* this subject reported at 33.3 months post-treatment that he was doing very well until about 30.3 months post-treatment. At this time he moved into a new house and was doing a lot of work that involved repetitive use of his right (index) arm. He started to have increased pain in his right shoulder. Because of this he had a recent subacromial corticosteroid injection (i.e., before the first study visit). At the time of the first study visit he had no pain in his right shoulder.

| **Subject** | **M** | **Study** | **R** | **Severity** | **Descriptions/Comments** |
| --- | --- | --- | --- | --- | --- |
| A11 | 0.1 | F | Possible | Moderate | **Index arm pain^a^** |
| A3 | 0.0 | F | Possible | Mild | Left mid-abdomen pain**^a^** |
| A5 | 0.0 | F | Possible | Mild | Pain mid-abdomen**^a^** |
| A2 | 0.5 | F | Possible | Mild | Cold**^a^** |
| A2 | 0.9 | F | Unlikely | Moderate | Shortness of Breath**^a^** |
| A5 | 1.2 | F | Unlikely | Moderate | Pulp necrosis with asymptomatic apical periodontitis Tooth #15**^a^** |
| A4 | 0.7 | F | Unlikely | Mild | **Worsening index shoulder pain^a^** |
| A1 | 1.4 | F | Unlikely | Mild | **Worsening index shoulder pain^a^** |
| A11 | 18.7 | P | Unlikely | Mild | **Index shoulder pain^a^** |
| A1 | 25.5 | P | Unlikely | Mild | **Index shoulder pain^a^** |
| A4 | 3.0 | F | Not Related | Severe | Non ST elevation myocardial infarction**^b^** |
| A4 | 4.1 | F | Not Related | Severe | ST elevation myocardial infarction**^b^** |
| A9 | 7.4 | F | Not Related | Severe | **Index shoulder pain^b^** |
| A7 | 34.4 | P | Not Related | Severe | Ganglion cyst of the non-index shoulder**^b^** |
| A7 | 1.3 | F | Not Related | Moderate | Concussion |
| A6 | 1.9 | F | Not Related | Moderate | Worsening tendonitis left wrist |
| A9 | 7.6 | F | Not Related | Moderate | Bronchitis |
| A7 | 8.0 | F | Not Related | Moderate | Esophageal dysphagia |
| A7 | 9.7 | F | Not Related | Moderate | Chronic obstructive lung disease |
| A1 | 9.8 | F | Not Related | Moderate | Diverticulitis |
| A7 | 12.0 | F | Not Related | Moderate | Shortness of breath |
| A3 | 33.5 | P | Not Related | Moderate | **Index shoulder pain^c^** |
| A4 | 34.2 | P | Not Related | Moderate | Interstitial pneumonia |
| A8 | 34.9 | P | Not Related | Moderate | Alcoholic relapse |
| A4 | 36.8 | P | Not Related | Moderate | Right leg pain |
| A8 | 38.7 | P | Not Related | Moderate | Dyspnea |
| A7 | 40.4 | P | Not Related | Moderate | Muscle spasms |
| A9 | -0.3 | F | Not Related | Mild | Asthmatic bronchitis |
| A9 | 0.3 | F | Not Related | Mild | Left knee pain |
| A8 | 0.6 | F | Not Related | Mild | Dysuria |

**Supplementary Table S1 (cont)**.

| **Subject** | **M** | **Study** | **R** | **Severity** | **Descriptions/Comments** |
| --- | --- | --- | --- | --- | --- |
| A9 | 1.0 | F | Not Related | Mild | Contusion of right elbow |
| A3 | 1.1 | F | Not Related | Mild | Worsening lower back pain |
| A7 | 1.6 | F | Not Related | Mild | Sinusitis |
| A2 | 2.1 | F | Not Related | Mild | Worsening allergic rhinitis |
| A7 | 2.8 | F | Not Related | Mild | Abdominal discomfort |
| A11 | 3.9 | F | Not Related | Mild | Left hand pain |
| A10 | 4.1 | F | Not Related | Mild | Toenail thickening and discoloration |
| A10 | 4.1 | F | Not Related | Mild | Seborrheic keratosis |
| A2 | 4.2 | F | Not Related | Mild | Essential hypertension |
| A1 | 4.4 | F | Not Related | Mild | Pharyngitis |
| A8 | 4.6 | F | Not Related | Mild | Relapse of alcohol dependence |
| A11 | 4.6 | F | Not Related | Mild | Staph infection of fourth ray |
| A7 | 5.5 | F | Not Related | Mild | Acute pain left shoulder |
| A8 | 6.8 | F | Not Related | Mild | Left foot pain |
| A11 | 31.4 | P | Not Related | Mild | influenza |
| A9 | 32.7 | P | Not Related | Mild | **Device material fragmentation in index shoulder^b^** |
| A10 | 34.1 | P | Not Related | Mild | Epigastric pain |
| A4 | 34.3 | P | Not Related | Mild | Sleep apnea |
| A9 | 35.7 | P | Not Related | Mild | Bronchhitis |
| A11 | 36.7 | P | Not Related | Mild | Sleep disturbance |
| A11 | 36.7 | P | Not Related | Mild | Tinnitus |
| A11 | 36.7 | P | Not Related | Mild | Hearing loss |
| A8 | 37.2 | P | Not Related | Mild | Acute cystitis |
| A9 | 39.5 | P | Not Related | Mild | Bursal hematoma left knee |
| A4 | 40.4 | P | Not Related | Mild | Benign prostatic hyperplasia |
| A5 | 40.6 | P | Not Related | Mild | Cataract |
| A5 | 40.9 | P | Not Related | Mild | Back pain |
| A7 | 41,4 | P | Not Related | Mild | Baclofen allergy |

**Supplementary Table S2.** Treatment-emergent adverse events reported by those subjects who were treated with injection of corticosteroid. Adverse events related to the index shoulder are given boldface. *M* month post-treatment when the treatment-emergent adverse event started, *F* former study, *P* present study, *R* relation to the investigated treatment, *^a^* outlined in detail in Supplementary Table S9, *^b^* this subject was enrolled in the former study but not in the present study, *^c^* this subject fell off a ladder at 32.3 months post-treatment. He reported increased pain and discomfort and difficulty with overhead activity. An MRI performed during the second study visit at 33.3 months post-treatment revealed a full thickness tear of anterior superior fibers of the supraspinatus tendon. An MRI performed during the second study visit at 41.2 months post-treatment showed a near full thickness tear of the anterosuperior fibers of the supraspinatus tendon. *^d^* this subject reported pain in his left shoulder at 37.2 months post-treatment that kept him awake at night; he was unable to sleep on his left side.

| **Subject** | **M** | **Study** | **R** | **Severity** | **Descriptions/Comments** |
| --- | --- | --- | --- | --- | --- |
| C4 | 1.4 | F | Possible | Moderate | **Anterior pain in index shoulder^a^** |
| C2 | 1.6 | F | Possible | Moderate | **Worsening index shoulder pain^a^** |
| C2 | 0.1 | F | Unlikely | Mild | Pharyngitis**^a^** |
| C5**^b^** | 2.1 | F | Unlikely | Mild | **Worsening index shoulder pain^a^** |
| C3 | 5.2 | F | Unlikely | Mild | **Worsening index shoulder pain^a^** |
| C3 | 1.0 | F | Not Related | Moderate | Biceps tear at elbow |
| C2 | 5.4 | F | Not Related | Moderate | Right upper broken tooth |
| C1 | 36.1 | P | Not Related | Moderate | Multilevel lumbar degenerative disc disease |
| C2 | 38.2 | P | Not Related | Moderate | Actinic keratoses |
| C2 | 38.2 | P | Not Related | Moderate | Squamous cell carcinoma of skin, left neck |
| C2 | 38.2 | P | Not Related | Moderate | Basal cell carcinoma, left inferior neck |
| C5**^b^** | 0.7 | F | Not Related | Mild | Coronary artery disease |
| C3 | 1.1 | F | Not Related | Mild | Left finger sprain |
| C2 | 3.7 | F | Not Related | Mild | Hip Pain |
| C1 | 4.8 | F | Not Related | Mild | Chest pain |
| C2 | 4.8 | F | Not Related | Mild | Cough |
| C2 | 32.3 | P | Not Related | Mild | **Index shoulder pain^c^** |
| C4 | 32.3 | P | Not Related | Mild | Upper respiratory infection |
| C3 | 34.9 | P | Not Related | Mild | Benign skin lesion |
| C1 | 35.3 | P | Not Related | Mild | Otitis media |
| C4 | 36.5 | P | Not Related | Mild | Hypogammaglobulinemi**^a^** |
| C1 | 37.2 | P | Not Related | Mild | **Index shoulder pain^d^** |
| C2 | 37.8 | P | Not Related | Mild | Nausea |
| C2 | 40.5 | P | Not Related | Mild | Lymphadenopathy |
| C2 | 40.5 | P | Not Related | Mild | Atrial fibrillation |

**2.2. Statistical analysis of all** **treatment-emergent adverse events** **that occurred during the present and the former studies**

The total number of TEAEs was 83, of which 58 (69.9%) occurred in the UA-ADRCs group and 25 (30.1%) in the corticosteroid group (Supplementary Tables S1 and S2). Accordingly, the average number of TEAEs per subject was 5.3 ± 2.7 (mean ± standard error of the mean) in the UA-ADRCs group and 5.0 ± 1.8 in the corticosteroid group.

The distribution of these 83 TEAEs with regard to severity and relation to the investigated treatment was as follows: there were…

- four severe TEAEs (4.8%), none of which were related to the investigated treatment (all in the UA-ADRCs group),
- three moderate TEAEs probably related to the investigated treatment (3.6%) (one in the UA-ADRCs group and two in the corticosteroid group),
- three mild TEAEs probably related to the investigated treatment (3.6%) (all in the UA-ADRCs group),
- two moderate TEAEs unlikely to be related to the investigated treatment (2.4%) (all in the UA-ADRCs group),
- seven mild TEAEs unlikely to be related to the investigated treatment (8.4%) (four in the UA-ADRCs group and three in the corticosteroid group),
- 19 moderate TEAEs not related to the investigated treatment (22.9%), and
- 45 mild TEAEs not related to the investigated treatment (54.2%).

Supplementary Table S3 shows group-specific numbers of subjects who experienced a certain number of TEAEs (between 0 and 12) in the present and the former studies.

**Supplementary Table S3.** Group-specific numbers of subjects who experienced a certain number of treatment-emergent adverse events (between 0 and 12) in the present and the former studies. *TP* time period, *BL* baseline, *W24* study visit scheduled in the former study at 24 weeks post-treatment, *FSV* first study visit of the present study, *SSV* second study visit of the present study, *UA-ADRCs* treatment with a single injection of fresh, uncultured, unmodified, autologous, adipose-derived regenerative cells, *C* treatment with a single injection of corticosteroid.

| **TP** | **Group** | **Number of TEAEs** | | | | | | | | | | | | |
| --- | --- | --- | --- | --- | --- | --- | --- | --- | --- | --- | --- | --- | --- | --- |
|  |  | **0** | **1** | **2** | **3** | **4** | **5** | **6** | **7** | **8** | **9** | **10** | **11** | **12** |
| BL-W24 | UA-ADRCs | 0 | 1 | 5 | 3 | 2 | 0 | 0 | 0 | 0 | 0 | 0 | 0 | 0 |
| BL-FSV | UA-ADRCs | 0 | 1 | 3 | 2 | 3 | 0 | 1 | 1 | 0 | 0 | 0 | 0 | 0 |
| BL-SSV | UA-ADRCs | 0 | 1 | 0 | 2 | 3 | 0 | 1 | 1 | 2 | 0 | 1 | 0 | 0 |
| BL-W24 | C | 0 | 2 | 1 | 1 | 0 | 1 | 0 | 0 | 0 | 0 | 0 | 0 | 0 |
| BL-FSV | C | 0 | 1 | 2 | 1 | 0 | 0 | 1 | 0 | 0 | 0 | 0 | 0 | 0 |
| BL-SSV | C | 0 | 0 | 1 | 1 | 2 | 0 | 0 | 0 | 0 | 0 | 0 | 0 | 1 |

For all investigated time periods there was no statistically significant difference between the groups with regard to the numbers of subjects who experienced a certain number of TEAEs (between 0 and 12) in the present and the former studies (Chi-square test for trend):

- from baseline to W24 in the former study: p = 0.809,
- from baseline to the first study visit of the present study: p = 0.488, and
- from baseline to the second study visit of the present study: p = 0.778.

Supplementary Table S4 shows group-specific numbers of TEAEs that were classified as {mild / moderate / severe} in the present and the former studies. Supplementary Table S5 summarizes absolute numbers of all TEAEs reported in the present and the former studies, stratified by the relation to the investigated treatment, severity and time period during which the TEAEs occurred. Supplementary Table S6 summarizes the corresponding mean numbers of TEAEs per subject in each group.

**Supplementary Table S4.** Group-specific numbers of TEAEs that were classified as {mild / moderate / severe} in the present and the former studies. *TP* time period, *BL* baseline, *W24* study visit scheduled in the former study at 24 weeks post-treatment, *FSV* first study visit of the present study, *SSV* second study visit of the present study, *UA-ADRCs* treatment with a single injection of fresh, uncultured, unmodified, autologous, adipose-derived regenerative cells, *C* treatment with a single injection of corticosteroid.

| **TP** | **Group** | **Classification of TEAEs** | | |
| --- | --- | --- | --- | --- |
|  |  | **Mild** | **Moderate** | **Severe** |
| BL-W24 | UA-ADRCs | 21 | 5 | 2 |
| BL-FSV | UA-ADRCs | 25 | 10 | 3 |
| BL-SSV | UA-ADRCs | 38 | 16 | 4 |
| BL-W24 | C | 8 | 4 | 0 |
| BL-FSV | C | 10 | 4 | 0 |
| BL-SSV | C | 17 | 8 | 0 |

For all investigated time periods there was no statistically significant difference between the groups with regard to the numbers of TEAEs that were classified as {mild / moderate / severe} in the present and the former studies (Chi-square test for trend):

- from baseline to W24 in the former study: p = 0.951,
- from baseline to the first study visit of the present study: p = 0.468, and
- from baseline to the second study visit of the present study: p = 0.497.

**Supplementary Table S5.** Absolute numbers of TEAEs reported in the present and the former studies, stratified by the relation to the investigated treatment, severity and time period during which the TEAEs occurred. *TP* time period, *BL* baseline, *W24* study visit scheduled in the former study at 24 weeks post-treatment, *FSV* first study visit of the present study, *SSV* second study visit of the present study, *UA-ADRCs* treatment with a single injection of fresh, uncultured, unmodified, autologous, adipose-derived regenerative cells, *C* treatment with a single injection of corticosteroid, *P* relation to the investigated treatment probable, *U* relation to the investigated treatment unlikely, *NR* no relation to the investigated treatment, *se* severe, *mo* moderate, *mi*, mild.

| **TP** | **Group** | **P-se** | **P-**  **mo** | **P-**  **mi** | **U-**  **se** | **U-**  **mo** | **U-**  **mi** | **NR-se** | **NR-mo** | **NR-mi** |
| --- | --- | --- | --- | --- | --- | --- | --- | --- | --- | --- |
| BL-W24 | UA-ADRCs | 0 | 1 | 3 | 0 | 2 | 2 | 2 | 2 | 16 |
| BL-FSV | UA-ADRCs | 0 | 1 | 3 | 0 | 2 | 4 | 3 | 7 | 18 |
| BL-SSV | UA-ADRCs | 0 | 1 | 3 | 0 | 2 | 4 | 4 | 13 | 31 |
| BL-W24 | C | 0 | 2 | 0 | 0 | 0 | 3 | 0 | 2 | 5 |
| BL-FSV | C | 0 | 2 | 0 | 0 | 0 | 3 | 0 | 2 | 7 |
| BL-SSV | C | 0 | 2 | 0 | 0 | 0 | 3 | 0 | 6 | 14 |

**Supplementary Table S6.** Group-specific mean numbers of treatment-emergent adverse events per subject reported in the present and the former studies, stratified by the relation to the investigated treatment, severity and time period during which the TEAEs occurred. *TP* time period, *BL* baseline, *W24* study visit scheduled in the former study at 24 weeks post-treatment, *FSV* first study visit of the present study, *SSV* second study visit of the present study, *UA-ADRCs* treatment with a single injection of fresh, uncultured, unmodified, autologous, adipose-derived regenerative cells, *C* treatment with a single injection of corticosteroid, *P* relation to the investigated treatment probable, *U* relation to the investigated treatment unlikely, *NR* no relation to the investigated treatment, *se* severe, *mo* moderate, *mi* mild.

| **TP** | **Group** | **P-**  **se** | **P-**  **mo** | **P-**  **mi** | **U-**  **se** | **U-**  **mo** | **U-**  **mi** | **NR-se** | **NR-mo** | **NR-mi** |
| --- | --- | --- | --- | --- | --- | --- | --- | --- | --- | --- |
| BL-W24 | UA-ADRCs | 0 | 0.09 | 0.27 | 0 | 0.18 | 0.18 | 0.18 | 0.18 | 1.45 |
| BL-FSV | UA-ADRCs | 0 | 0.09 | 0.27 | 0 | 0.18 | 0.36 | 0.27 | 0.64 | 1.64 |
| BL-SSV | UA-ADRCs | 0 | 0.09 | 0.27 | 0 | 0.18 | 0.36 | 0.36 | 1.18 | 2.82 |
| BL-W24 | C | 0 | 0.40 | 0 | 0 | 0 | 0.60 | 0 | 0.40 | 1.00 |
| BL-FSV | C | 0 | 0.40 | 0 | 0 | 0 | 0.60 | 0 | 0.40 | 1.40 |
| BL-SSV | C | 0 | 0.40 | 0 | 0 | 0 | 0.60 | 0 | 1.20 | 2.80 |

Supplementary Table S7 shows group-specific numbers of TEAEs that were classified as {not related / unlikely to be related / possibly related / probably related / definitely related} to the investigated treatment in the present and the former studies. Supplementary Tables S8 and S9 summarize the individual courses of all TEAEs classified as {unlikely to be / possibly} related to the investigated treatment that occurred during the present and the former studies, experienced by those subjects who were treated with injection of UA-ADRCs (Supplementary Table 8) and those subjects who were treated with injection of corticosteroid (Supplementary Table S9).

**Supplementary Tabe S7.** Group-specific numbers of TEAEs that were classified as {not related / unlikely to be related / possibly related / probably related / definitely related} to the investigated treatment in the present and the former studies. *TP* time period, *BL* baseline, W24 study visit scheduled in the former study at 24 weeks post-treatment, *FSV* first study visit of the present study, *SSV* second study visit of the present study, *UA-ADRCs* treatment with a single injection of fresh, uncultured, unmodified, autologous, adipose-derived regenerative cells, *C* treatment with a single injection of corticosteroid.

| **TP** | **Group** | **Classification of TEAEs** | | | | |
| --- | --- | --- | --- | --- | --- | --- |
|  |  | **not related** | **unlikely to be related** | **possibly related** | **probably related** | **pefinitely related** |
| BL-W24 | UA-ADRCs | 20 | 4 | 4 | 0 | 0 |
| BL-FSV | UA-ADRCs | 28 | 6 | 4 | 0 | 0 |
| BL-SSV | UA-ADRCs | 48 | 6 | 4 | 0 | 0 |
| BL-W24 | C | 7 | 3 | 2 | 0 | 0 |
| BL-FSV | C | 9 | 3 | 2 | 0 | 0 |
| BL-SSV | C | 20 | 3 | 2 | 0 | 0 |

For all investigated time periods there was no statistically significant difference between the groups with regard to the numbers of TEAEs that were classified as {not related / unlikely to be related / possibly related / probably related / definitely related} to the investigated treatment in the present and the former studies (Chi-square test):

- from baseline to W24 in the former study: p = 0.672,
- from baseline to the first study visit of the present study: p = 0.802, and
- from baseline to the second study visit of the present study: p = 0.956.

**Supplementary Table S8.** Individual courses of the treatment-related adverse events classified as {unlikely to be / possibly related} to the investigated treatment that occurred during the present and the former studies, experienced by those subjects who were treated with injection of UA-ADRCs. *UA-ADRCs*, treatment with a single injection of fresh, uncultured, unmodified, autologous, adipose-derived regenerative cells, *M* month post-treatment.

| **M** | **Event** |
| --- | --- |
| **Subject A1: worsening index shoulder pain (first TEAE of Subject A1), classified as *mild* and *unlikely* related to the investigated treatment** | |
| 1.4 | Subject woke up in the morning of this day with worsening pain in left (index) shoulder. The pain lasted about 4-6 hours and was then back to baseline. No action was taken by the subject to alleviate the pain. |
| **Subject A1: index shoulder pain (second TEAE of Subject A1), classified as *mild* and *unlikely* related to the investigated treatment** | |
| 28.4 | Subject reported development of pain in the lateral index (left) shoulder over the past few months. Pain was worse with lifting or overhead movement. The subject received a subacromial corticosteroid injection at this time. |
| 32.9 | Subject rated shoulder pain 3-4/10 during an office visit and received another subacromial corticosteroid injection. |
| 34.7 | Subject reported left shoulder pain 0/10 but expressed interest in proceeding with left shoulder rotator cuff repair during the next few months. |
| 36.5 | Subject reported that he started having left shoulder pain at night and treating with Tylenol as needed. |
| 36.8 | Subject underwent left shoulder arthroscopy with subacomial decompression and rotator cuff repair. |
| 37.5 | The coordinator followed up with the subject who reported that the left shoulder pain at night had resolved since surgery. |
| 39.7 | Subject reported a muscle knot in the left arm with pain during therapy and other times. Tissue massage, ultrasound and electrical stimulation did not help much. The subject exhausted post-op physical therapy sessions. |
| 43.0 | Subject transitioned to occupational therapy to continue work on the left shoulder for range of motion and strength. |
| **Subject A2: cold (first TEAE of Subject A2), classified as *mild* and *possibly* related to the investigated treatment** | |
| 0.5 | Subject reported cough and cold signs and symptoms. There was no fever. The subject took Airborn and Mucinex for a few days and both symptoms and cold went away. |

**Supplementary Table S8 (cont.)**

| **M** | **Event** |
| --- | --- |
| **Subject A2: shortness of breath (second TEAE of Subject A2), classified as *moderate* and *unlikely* related to the investigated treatment** | |
| 0.9 | Subject was seen in the emergency room (ER) for shortness of breath on 03/29/2017. EKG, chest X'ray and laboratory tests were unremarkable. Prednisone, Aspirin, normal saline and Solumedrol were given during the ER visit. The subject was sent home with a 5 day course of prednisone. |
| 1.4 | Subject reported continuous shortness of breath. |
| 2.0 | Subject reported that shortness of breath was not resolved but improving. |
| 2.7 | Subject reported that the shortness of breath continued, but only with activity. The subject reported history of exercised induced asthma as a child. |
| 4.0 | A pulmonary function test did not show any abnormalities. There were symptoms of wheezing, chest tightness, sputum production and shortness of breath with conversation of a dyspnea on exertion. A bronchoscopy was scheduled. |
| 4.3 | A bronchoscopy showed left upper lobe narrowed/edematous likely with variable obstruction based on airflow and edema. There was increased pitting of airway based on airflow and edema. Bronchial washings sent for culture were positive for <10,000 CFU/mL *Streptococcus alpha*, which is consistent with normal respiratory flora. |
| 4.5 | The pulmonologist started on Amoxicillin x 7 days, Arnuity inhaler and Albuterol inhaler. |
| 4.8 | An echocardiogram was normal. |
| 7.4 | There was shortness of breath, decreased voice and chest wall tightness. A chest X-ray showed no change. Complete blood count and sedimentation rate were normal. |
| 7.7 | The symptoms of the subject were considered incompletely controlled asthma based on symptoms alone. Therapy with Breo Ellipta daily was started, and the subject was advised to use the Albuterol inhaler consistently. Furthermore, it was recommend to start Fexfofenadine for seasonal allergies to help control symptoms. |
| 14.7 | Subject reported via phone that shortness of breath was resolved with laryngoplasty surgery at 9.4 months post-treatment. |
| **Subject A3: left mid-abdomen pain, classified as *mild* and *possibly* related to the investigated treatment** | |
| 0.0 | Subject reported that he had intermittent pain since his liposuction procedure. There was minimal focal tenderness. The subject reported that he was getting better and was overall not concerned. |
| **Subject A4: worsening index shoulder pain, classified as *mild* and *unlikely* related to the investigated treatment** | |
| 0.7 | Subject reported that he went back to work. He is a truck driver and he noticed that since then his shoulder pain was worse. |
| **Subject A5: mid-abdomen pain (first TEAE of Subject A5), classified as *mild* and *possibly* related to the investigated treatment** | |
| 0.0 | Subject reported pain and swelling of the mid-abdomen for two days. |
| **Subject A5: pulp necrosis with asymptomatic apical periodontitis Tooth #15 (second TEAE of Subject A5), classified as *moderate* and *unlikely* related to the investigated treatment** | |
| 1.2 | A cone beam computed tomography scan showed a large lesion with significant bone loss. Thermal testing was unresponsive. |
| **Subject A11: pain in the arm of the index shoulder (first TEAE of Subject A11), classified as *moderate* and *possibly* related to the investigated treatment** | |
| 0.1 | Subject reported right arm pain, starting three days post shoulder injection. |

**Supplementary Table S8 (cont.)**

| **M** | **Event** |
| --- | --- |
| **Subject A11: index shoulder pain (second TEAE of Subject A11), classified as *mild* and *unlikely* related to the investigated treatment** | |
| 18.7 | Subject reported worsening of pain in the index (right) shoulder. Pain experienced by the subject did not start for over a year after the target procedure and was most likely related to progression of other issues. In this regard the Principal Investigator (Dr. Lundeen) recalled that there was some discussion prior to target treatment about the eligibility of the subject and that there were some bony changes at baseline. |
| 24.0 | Arthroscopic examination revealed (i) a very small partial-thickness tearing at the far anterior border of the supraspinatus tendon, which extended approximately 10% to 15% through the substance of the tendon with no exposed footprint; (ii) Grade 2 chondromalacia involving the superolateral humeral head; (iii) unstable attachment site of the biceps tendon and labrum with some surrounding erythema at the synovium; (iv) a large anterior acromial osteophyte Type 2 bordering on Type 3; and (v) considerable degenerative change of the AC joint with a significant inferior osteophyte. On this basis the following surgical procedures were performed on the same day: (a) gentle debridement of partial-thickness tearing at the far anterior border of the supraspinatus tendon; (b) debridement of Grade 2 chondromalacia involving the superolateral humeral head; (c) debridement of the attachment site of the biceps tendon and labrum; (d) tenotomy of the biceps tendon; (e) resection of the subacromial bursa; (f) converting the large (Type 2 bordering on Type 3) anterior acromial osteophyte to Type 1 using a cutting block technique; and (g) distal clavicle excision removing approximately 1 cm of the lateral clavicle.  According to the Principal Investigator (M.L.) there is no need to assess the situation of this subject during the present study as failure of the initial index treatment. |

**Supplementary Table S9.** Individual courses of the treatment-related adverse events classified as {unlikely to be / possibly} related to the investigated treatment that occurred during the present and the former studies, experienced by those subjects who were treated with injection of corticosteroid. *M* month post-treatment, *BL* baseline.

| **M** | **Event** |
| --- | --- |
| **Subject C2: worsening index shoulder pain, classified as *moderate* and *possibly* related to the investigated treatment.** | |
| 1.6 | Subject reported musculoskeletal pain. Specifically, pain in the index shoulder fared up at times but then usually went back to baseline. |
| 8.0 | Subject reported that at this time his shoulder pain was back to its baseline. |
| **Subject C2: pharyngitis, classified as *mild* and *unlikely* related to the investigated treatment** | |
| 0.1 | Subject reported a sore throat for a few days, but did not take any medications for it. The sore throat went away on its own. |
| **Subject C3: worsening index shoulder pain, classified as *mild* and *unlikely* related to the investigated treatment** | |
| 5.2 | Subject stated definitely getting some pain in the index shoulder. |
| **Subject C4: anterior pain in the index shoulder, classified as *moderate* and *possibly* related to the investigated treatment** | |
| 1.4 | Subject complained of some anterior shoulder pain. |
| 7.4 | Subject received an additional corticosteroid injection into the index shoulder due to increased pain. |
| **Subject C5 in the former study: worsening index shoulder pain, classified as *mild* and *unlikely* related to the investigated treatment.** | |
| BL | MRI evaluation showed a partial-thickness tear of the supraspinatus tendon with the following dimensions: anterior-posterior extension, 9.5 mm; medial-lateral extension, 3.1 mm; tear depth, 2.8 mm; tear volume (calculated as volume of an ellipsoid), 43.2 mm^3^. |
| 5.2 | Subject reported definitely getting some pain in the index shoulder. |
| 7.3 | MRI evaluation showed a full-thickness tear of the supraspinatus tendon with the following dimensions: anterior-posterior extension, 10.0 mm (+0.5 mm [i.e., 105%] compared to baseline); medial-lateral extension, 13.0 mm (+9.9 mm [i.e., 420%] compared to baseline); tear depth, 6.0 mm (+3.2 mm [i.e., 210%] compared to baseline); tear volume, 408.4 mm^3^ (+365.2 mm^3^ [i.e., 950%] compared to baseline). In addition, MRI evaluation showed a dislocation of the long biceps tendon as well as a subscapularis tear of the index shoulder, which was not observerd at baseline. The subject withdrew consent after **diagnosis of full-thickness tear of the supraspinatus tendon** to seek alternative therapies or sugery and, thus, was lost to follow-up. Efficacy data of this subject beyond the examination performed at 12 weeks post-treatment in the former study were not collected, and the subject was not enrolled into the present study. |

Supplementary Table S10 shows group-specific numbers of TEAEs that were classified as {mild and unlikely to be related to the investigated treatment / mild and possibly related to the investigated treatment / moderate and unlikely to be related to the investigated treatment / moderate and possibly related to the investigated treatment} in the present and the former studies.

**Supplementary Table S10.** Group-specific numbers of TEAEs that were classified as {mild and unlikely to be related to the investigated treatment / mild and possibly related to the investigated treatment / moderate and unlikely to be related to the investigated treatment / moderate and possibly related to the investigated treatment} in the present and the former studies. *TP* time period, *BL* baseline, *W24* study visit scheduled in the former study at 24 weeks post-treatment, *FSV* first study visit of the present study, *SSV* second study visit of the present study, *UA-ADRCs* treatment with a single injection of fresh, uncultured, unmodified, autologous, adipose-derived regenerative cells, *C* treatment with a single injection of corticosteroid.

| **TP** | **Group** | **Classification of TEAEs** | | | |
| --- | --- | --- | --- | --- | --- |
|  |  | **mild and unlikely to be related** | **mild and possibly related** | **moderate and unlikely to be related** | **moderate and possibly related** |
| BL-W24 | UA-ADRCs | 2 | 3 | 2 | 1 |
| BL-FSV | UA-ADRCs | 4 | 3 | 2 | 1 |
| BL-SSV | UA-ADRCs | 4 | 3 | 2 | 1 |
| BL-W24 | C | 3 | 0 | 0 | 2 |
| BL-FSV | C | 3 | 0 | 0 | 2 |
| BL-SSV | C | 3 | 0 | 0 | 2 |

For all investigated time periods there was no statistically significant difference between the groups with regard to the numbers of TEAEs that were classified as {mild and unlikely to be related to the investigated treatment / mild and possibly related to the investigated treatment / moderate and unlikely to be related to the investigated treatment / moderate and possibly related to the investigated treatment} in the present and the former studies (Chi-square test for trend):

- from baseline to W24 in the former study: p = 0.941,
- from baseline to the first study visit of the present study: p = 0.757, and
- from baseline to the second study visit of the present study: p = 0.757.

**2.3. Severe treatment emergent adverse events**

Supplementary Tables S11-S13 summarize the individual courses of the four severe TEAEs that occurred during the present and the former studies, all of which were classified as not related to the investigated treatment.

**Supplementary Table S11.** Severe TEAE experienced by Subject A7 who was treated with UA-ADRCs. *UA-ADRCs* treatment with a single injection of fresh, uncultured, unmodified, autologous, adipose-derived regenerative cells, *M* month post-treatment.

| **M** | **Event** |
| --- | --- |
| 5.5 | Subject reported acute pain in the left shoulder (i.e., not in the index shoulder). A subacromial bursitis impingement and a small undersurface tear of the rotator cuff were diagnosed, but no treatment was performed. |
| 34.4 | Subject was diagnosed with a **spinoglenoid notch cyst in the left shoulder (i.e., not in the index shoulder)** (**severe TEAE experienced by this subject**), discovered by MRI. Pain at times radiated up towards the neck, and at times down the arm with some pain all the way into the hand. |
| 36.9 | Arthroscopic decompression of the cyst and repair of the posterior capsulolabral tissue was performed. |
| 37.4 | Subject reported pain 4/10 and occasional use of Tylenol and ice, as well as use of a SlingShot immobilizer. There was still some swelling present at this time, but this was decreasing. |
| 38.3 | There was still some discomfort, together with slow progress in improvement of range of motion. |
| 40.3 | The pain was nearly resolved and restricted to holding the left arm at shoulder height for extended periods of time. External rotation was lacking about 10 degrees, and internal rotation was lacking about two to three interspace levels. |
| 41.2 | Subject reported that the stopped physical therapy due to increased pain when using heavy weights. |

Data regarding the SAE of Subject A7 (this SAE occurred during the present study) was sent to the Institutional Review Board of the study site, the Data and Safety Monitoring Board of the present study and the U.S. Food and Drug Administration, and all noted that this SAE was not related to study treatment. The SAE was treated per standard of care.

**Supplementary Table S12.** Severe TEAE experienced by Subject A9 who was treated with UA-ADRCs. *UA-ADRCs* treatment with a single injection of fresh, uncultured, unmodified, autologous, adipose-derived regenerative cells, *M* month post-treatment.

| **M** | **Event** |
| --- | --- |
| 1.0 | Subject suffered from a **contusion of the right elbow (i.e., on the same side as the index shoulder) due to a fall on ice**. This TEAE was classified as mild and not related to the investigated treatment. |
| 7.4 | subject felt **some pain in the index shoulder and a little bit of weakness secondary to the pain (severe TEAE experienced by this subject).** |
| 13.4 | A surgical rotator cuff repair procedure was performed (note that this surgery of the index shoulder took place outside of the present and the former studies). |
| 32.7 | MRI examination showed a fragment of a broken anchor from a rotator cuff repair procedure performed on, which had lodged itself just above the superior labrum. This fragment of a broken anchor appeared to have found a quiet zone within the joint and was not impacting subject's performance or function in any way. No treatment was planned. |

**Supplementary Table S13.** Severe TEAEs experienced by Subject A4 who was treated with UA-ADRCs. *UA-ADRCs* treatment with a single injection of fresh, uncultured, unmodified, autologous, adipose-derived regenerative cells, *M* month post-treatment.

| **M** | **Event** |
| --- | --- |
| 3.0 | Subject was diagnosed with a **non ST elevation myocardial infarction (first severe TEAE experienced by this subject)**. The subject had some recurring pain intermittently, but had been stable while awaiting catheter examination over the weekend. The decision to wait for catheter examination was due to platelet count of 50,000. Stress testing on the weekend indicated inferior ischemia. |
| 3.1 | ACE-inhibitor was stopped and i.v. fluids were given. Initially the subject was doing well, but developed chest pain when showering; ECG was suggestive of ST elevation. The subject was taken emergently to the catheter lab on the same day. Stents were placed in a previous vein graft to the right posterior descending artery (RPDA) and the native RPDA. The decision to split treatment into two procedures was due to concerns with his kidneys. |
| 3.1 | Stents were also placed in a previous vein graft to the lateral left anterior descending artery. The subject did well on the rest of his stay and was discharged a few days later. A cardiology follow up was planned. Atenolol was also stopped due to bradycardia concerns. |
| 4.3 | Subject had some feelings of fatigue and has noted some left sided chest discomfort and left arm pain into the antecubital area with walking and relieved with rest. Treatment with Imdur daily was started; a stress test was abnormal. The subject was diagnosed with a **ST elevation myocardial infarction (second severe TEAE experienced by this subject**). |
| 4.6 | A cardiac angiogram showed no lesions amenable to revascularization. Adjustments to current medications were performed. |
| 6.3 | A follow up doctor visit showed that the subject was symptomatically doing well with medication changes. |

**Part 4 – Scheduled study visits and availability of ASES Total score, VAS pain score and SF-36 Total score data**

Supplementary Table S14 lists the scheduled visits of the present and the former studies, during which the primary endpoint *long term efficacy of pain and function through ASES Shoulder Score and* *SF-36 health questionnaires* between the two groups was investigated, and indicates whether (+) or not (-) the corresponding subject developed additional pathologies of the index shoulder (next to symptomatic, partial-thickness rotator cuff tear) and/or received additional treatments on the index shoulder (next to injection of UA-ADRCs or corticosteroid) during either of these studies. The corresponding reasons are summarized in Supplementary Table S15.

**Supplementary Table S14.** Scheduled visits of the present and the former studies, during which the primary endpoint long term efficacy of pain and function through ASES Shoulder Score and SF-36 health questionnaires between the two groups was investigated, and indication whether (+) or not (-) the corresponding subject developed additional pathologies of the index shoulder (next to symptomatic, partial-thickness rotator cuff tear) and/or received additional treatments on the index shoulder (next to injection of UA-ADRCs or corticosteroid) during either of these studies. The remarks are outlined in Supplementary Table S15. *UA-ADRCs* treatment with a single injection of fresh, uncultured, unmodified, autologous, adipose-derived regenerative cells, *C* treatment with a single injection of corticosteroid, *BL* baseline, *W24* 24 weeks post-treatment in the former study, *FSV / SSV* first / second study visit in the present study.

| **Subject** | **Group** |  |  |  |  | **Remark** |
| --- | --- | --- | --- | --- | --- | --- |
|  |  | **BL** | **W24** | **FSV** | **SSV** |  |
| A1 | UA-ADRCs | - | - | + | + | a |
| A2 | UA-ADRCs | - | - | - | - |  |
| A3 | UA-ADRCs | - | - | + | + | b |
| A4 | UA-ADRCs | - | - | - | - |  |
| A5 | UA-ADRCs | - | - | - | - |  |
| A6 | UA-ADRCs | - | - | - | - |  |
| A7 | UA-ADRCs | - | - | - | - |  |
| A8 | UA-ADRCs | - | - | - | - |  |
| A9 | UA-ADRCs | - | + | + | + | c |
| A10 | UA-ADRCs | - | - | - | - |  |
| A11 | UA-ADRCs | - | - | + | + | d |
| C1 | C | - | - | - | - |  |
| C2 | C | - | - | + | + | e |
| C3 | C | - | - | - | - |  |
| C4 | C | - | - | + | + | f |
| C5 | C | - | + | + | + | g |

**Supplementary Table S15.** Reasons why individual subjects enrolled in the present and the former studies developed additional pathologies of the index shoulder (next to symptomatic, partial-thickness rotator cuff tear) and/or received additional treatments on the index shoulder (next to injection of UA-ADRCs or corticosteroid) during either of these studies. The remarks refer to Supplementary Table S14. *M* months post-treatment.

| **M** | | **Event** |
| --- | --- | --- |
| **Remark a in Supplementary Table S14 – Subject A1** | | |
| 28.4 | Psubject reported development of pain in the lateral index (left) shoulder over the past few months. Pain was worse with lifting or overhead movement. The subject received a subacromial corticosteroid injection at this time. | |
| 32.9 | Subject rated shoulder pain 3-4/10 and received another subacromial corticosteroid injection. | |
| 34.7 | Subject reported left shoulder pain 0/10 but expressed interest in proceeding with left shoulder rotator cuff repair during the next few months. | |
| 36.5 | Subject reported that he started having left shoulder pain at night and treating with Tylenol as needed. | |
| 36.8 | Subject underwent left shoulder arthroscopy with subacomial decompression and rotator cuff repair. | |
| 37.5 | The study coordinator followed up with the subject. The subject reported the left shoulder pain at night resolved since surgery. | |
| 39.7 | Subject reported a muscle knot in left arm, with pain during therapy and other times. Tissue massage, ultrasound and electrical stimulation did not help much. The subject exhausted post-op physical therapy sessions. | |
| 40.1 | Subject had a nonstudy visit, and notes from this nonstudy visit were used to complete concomitant medications and adverse events. | |
| 43.0 | Subject transitioned to occupational therapy in order to continue work on the left shoulder for range of motion and strength. | |
| **Remark b in Supplementary Table S14 – Subject A3** | | |
| 33.3 | Subject reported that he was doing very well until 30.3 months post-treatment. At this time the subject moved into a new house and was doing a lot of work that involved repetitive use of his right (index) arm. The subject started to have increased pain in his right shoulder. Because of this the subject had a recent subacromial corticosteroid injection (i.e., before the first study visit). At the time of the first study visit the subject had no pain in his right shoulder. | |
| **Remark c in Supplementary Table S14 – Subject A9** | | |
| 6.3 | Subject reported a contusion of the right elbow due to fall on ice; the start date of this TEAE was at 1.0 month post-treatment. | |
| 7.4 | Subject had some pain in the right (index) shoulder and a little bit of weakness secondary to the pain. | |
| 32.7 | MRI showed a fragment of a broken anchor from a rotator cuff repair procedure performed at 13.4 months post-treatment, which had lodged itself just above the superior labrum. This fragment of a broken anchor appeared to have found a quiet zone within the joint and was not impacting subject's performance or function in any way. No treatment was planned. | |

**Supplementary Table S15 (cont.)**

| **M** | | **Event** |
| --- | --- | --- |
| **Remark d in Supplementary Table S14 – Subject A11** | | |
| 18.7 | Subject reported worsening of pain in the index (right) shoulder. Pain experienced by the subject did not start for over a year after the target procedure and was most likely related to progression of other issues. In this regard the Principal Investigator (Dr. Lundeen) recalled that there was some discussion prior to target treatment about the eligibility of the subject and that there were some bony changes at baseline. | |
| 24.0 | Arthroscopic examination revealed (i) a very small partial-thickness tearing at the far anterior border of the supraspinatus tendon, which extended approximately 10% to 15% through the substance of the tendon with no exposed footprint; (ii) Grade 2 chondromalacia involving the superolateral humeral head; (iii) unstable attachment site of the biceps tendon and labrum with some surrounding erythema at the synovium; (iv) a large anterior acromial osteophyte Type 2 bordering on Type 3; and (v) considerable degenerative change of the AC joint with a significant inferior osteophyte. On this basis the following surgical procedures were performed on the same day: (a) gentle debridement of partial-thickness tearing at the far anterior border of the supraspinatus tendon; (b) debridement of Grade 2 chondromalacia involving the superolateral humeral head; (c) debridement of the attachment site of the biceps tendon and labrum; (d) tenotomy of the biceps tendon; (e) resection of the subacromial bursa; (f) converting the large (Type 2 bordering on Type 3) anterior acromial osteophyte to Type 1 using a cutting block technique; and (g) distal clavicle excision removing approximately 1 cm of the lateral clavicle.  According to the Principal Investigator (M.L.) there is no need to assess the situation of this subject during the present study as failure of the initial index treatment. | |
| **Remark e in Supplementary Table S14 – Subject C2** | | |
| 32.3 | Subject fell off a ladder. He reported increased pain and discomfort and difficulty with overhead activity. | |
| 33.3 | An MRI revealed a full thickness tear of anterior superior fibers of the supraspinatus tendon. | |
| 41.2 | A second MRI showed a near full thickness tear of the anterosuperior fibers of the supraspinatus tendon. | |
| **Remark f in Supplementary Table S14 – Subject C4** | | |
| 1.4 | Subject complained of some anterior shoulder pain. | |
| 7.4 | Subject received an additional corticosteroid injection into the index shoulder due to increased pain. | |
| **Remark g in Supplementary Table S14 – Subject C5** | | |
| BL | MRI evaluation showed a partial-thickness tear of the supraspinatus tendon with the following dimensions: anterior-posterior extension, 9.5 mm; medial-lateral extension, 3.1 mm; tear depth, 2.8 mm; tear volume (calculated as volume of an ellipsoid), 43.2 mm^3^. | |
| 2.1 | Subject reported definitely getting some pain in the index shoulder. | |
| 3.7 | MRI evaluation showed a full-thickness tear of the supraspinatus tendon with the following dimensions: anterior-posterior extension, 10.0 mm (+0.5 mm [i.e., 105%] compared to baseline); medial-lateral extension, 13.0 mm (+9.9 mm [i.e., 420%] compared to baseline); tear depth, 6.0 mm (+3.2 mm [i.e., 210%] compared to baseline); tear volume, 408.4 mm^3^ (+365.2 mm^3^ [i.e., 950%] compared to baseline). In addition, MRI evaluation showed a dislocation of the long biceps tendon as well as a subscapularis tear of the index shoulder, which was not observerd at baseline. The subject withdrew consent after **diagnosis of full-thickness tear of the supraspinatus tendon** to seek alternative therapies or sugery and, thus, was lost to follow-up. Efficacy data of this subject beyond the examination performed at 12 weeks post-treatment in the former study were not collected, and the subject was not enrolled into the present study. | |

**Part 5 – Efficacy data as a function of time post-treatment**

**Supplementary Figure S18** (on the next page). Individual ASES Total score data as a function of time post-treatment of subjects treated with injection of either UA-ADRCs (Subjects A1-A11) or corticosteroid (Subjects C1-C5). The data are arranged in descending order of individual treatment success (i.e., the data of the subjects with the hightest ASES Total score are shown in the top row of the left column (Subject A2 treated with injection of UA-ADRCs) and the right column (Subject C3 treated with injection of corticosteroid). In the graphs (i) green dots and green lines indicate data of the present and the former studies that were available and could be used to assess treatment outcome, (ii) black dots and black lines (Subjects A11, A13 and C2) indicate data of the present study that were imputed uisng the Last Observation Carried Forward approach and could be used to assess treatment outcome (reasons are indicated in the corresponding panels and outlined in detail in Supplementary Table S15), and (iii) red dots and red lines (Subjects A1, C4 and C5) indicate data of the present and the former studies that were imputed as "failures" (reasons are indicated in the corresponding panels and outlined in detail in Supplementary Table S15). *A* accident affecting the index shoulder, *C* injection of corticosteroid into the index shoulder, *P* pain in the index shoulder, *S* surgery of the index shoulder.

**Supplementary Figure S18 (cont.)**


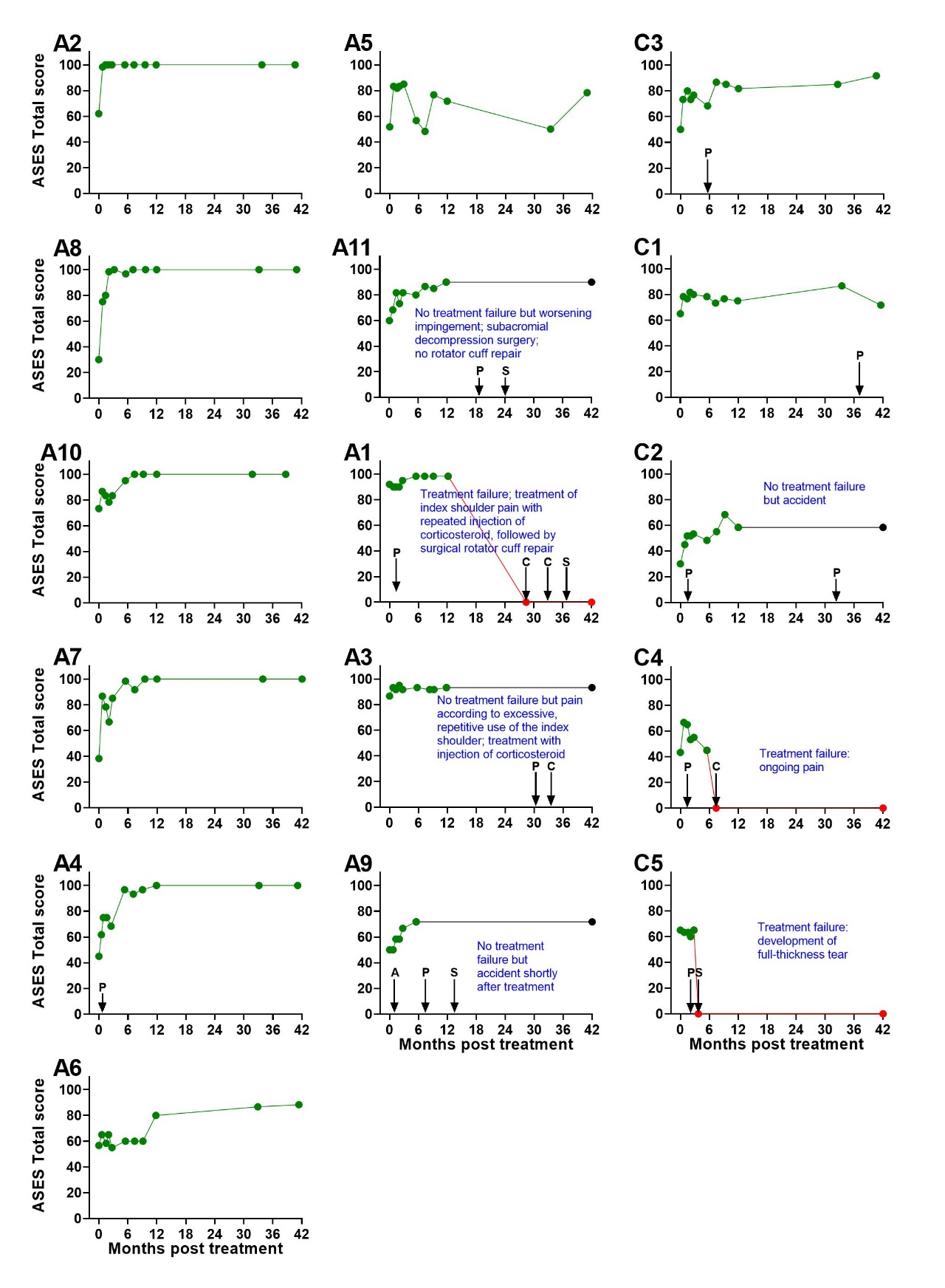


**Supplementary Figure S19** (on the next page). Individual SF-36 Total score as a function of time post-treatment of subjects treated with injection of either UA-ADRCs (Subjects A1-A11) or corticosteroid (Subjects C1-C5). The data are arranged in descending order of individual treatment success (i.e., the data of the subjects with the hightest ASES Total scores are shown in the top row of the left column (Subject A2 treated with injection of UA-ADRCs) and the right column (Subject C3 treated with injection of corticosteroid) (c.f. Supplementary Fig. 18). In the graphs (i) green dots and green lines indicate data of the present and the former studies that were available and could be used to assess treatment outcome, (ii) black dots and black lines (Subjects A11, A13 and C2) indicate data of the present study that were imputed uisng the Last Observation Carried Forward approach and could be used to assess treatment outcome (reasons are indicated in the corresponding panels and outlined in detail in Supplementary Table S15), and (iii) red dots and red lines (Subjects A1, C4 and C5) indicate data of the present and the former studies that were imputed as "failures" (reasons are indicated in the corresponding panels and outlined in detail in Supplementary Table S15). *A* accident affecting the index shoulder, *C* injection of corticosteroid into the index shoulder, *P* pain in the index shoulder, *S* surgery of the index shoulder.

**Supplementary Figure S19 (cont.)**


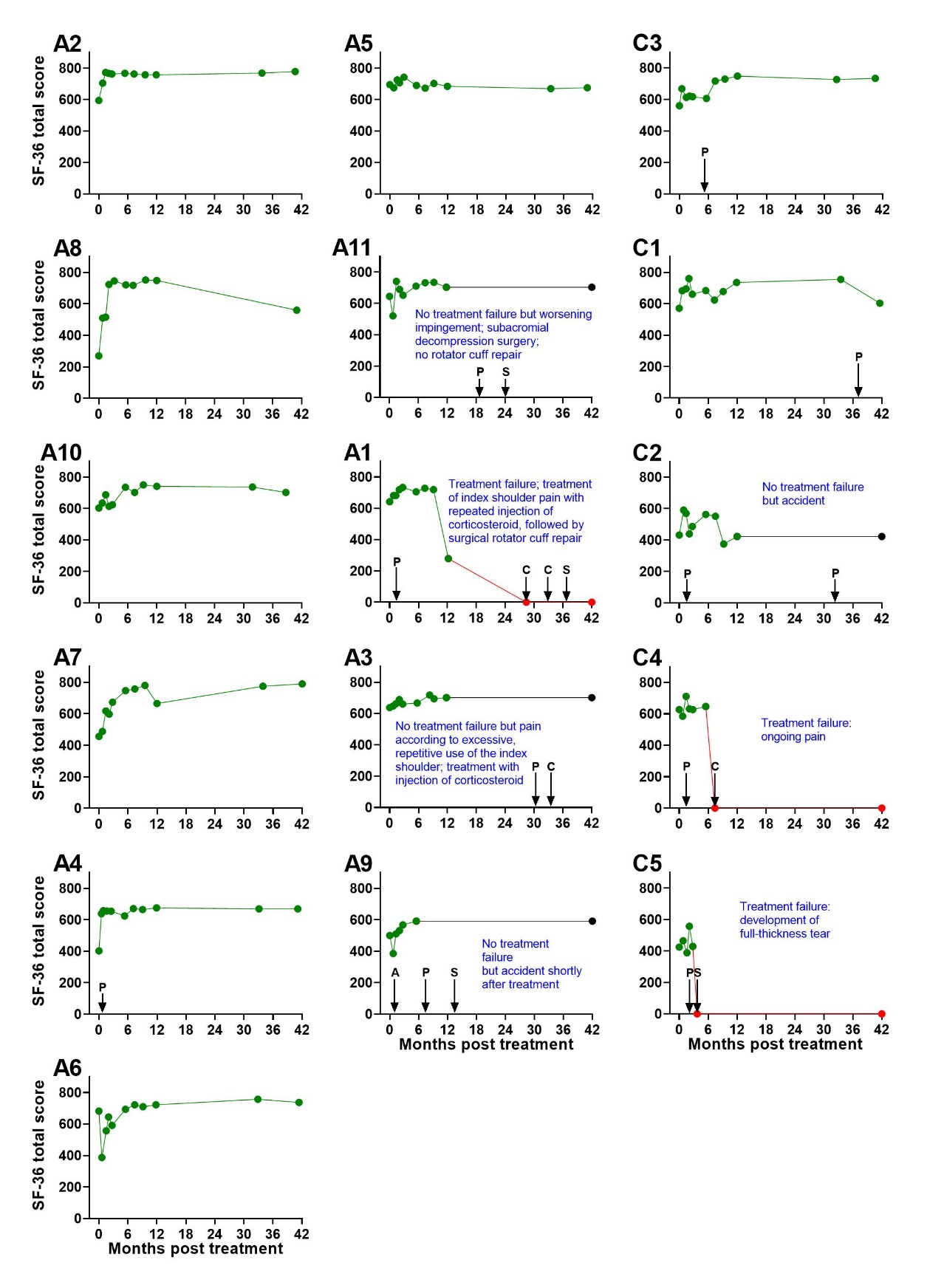


**Supplementary Figure S20** (on the next page). Individual VAS pain score (collected together with the ASES score) as a function of time post-treatment of subjects treated with injection of either UA-ADRCs (Subjects A1-A11) or corticosteroid (Subjects C1-C5). The data are arranged in descending order of individual treatment success (i.e., the data of the subjects with the hightest ASES Total scores are shown in the top row of the left column (Subject A2 treated with injection of UA-ADRCs) and the right column (Subject C3 treated with injection of corticosteroid) (c.f. Supplementary Fig. 18). In the graphs (i) green dots and green lines indicate data of the present and the former studies that were available and could be used to assess treatment outcome, (ii) black dots and black lines (Subjects A11, A13 and C2) indicate data of the present study that were imputed uisng the Last Observation Carried Forward approach and could be used to assess treatment outcome (reasons are indicated in the corresponding panels and outlined in detail in Supplementary Table S15), and (iii) red dots and red lines (Subjects A1, C4 and C5) indicate data of the present and the former studies that were imputed as "failures" (reasons are indicated in the corresponding panels and outlined in detail in Supplementary Table S15). *A* accident affecting the index shoulder, *C* injection of corticosteroid into the index shoulder, *P* pain in the index shoulder, *S* surgery of the index shoulder.

**Supplementary Figure S20 (cont.)**


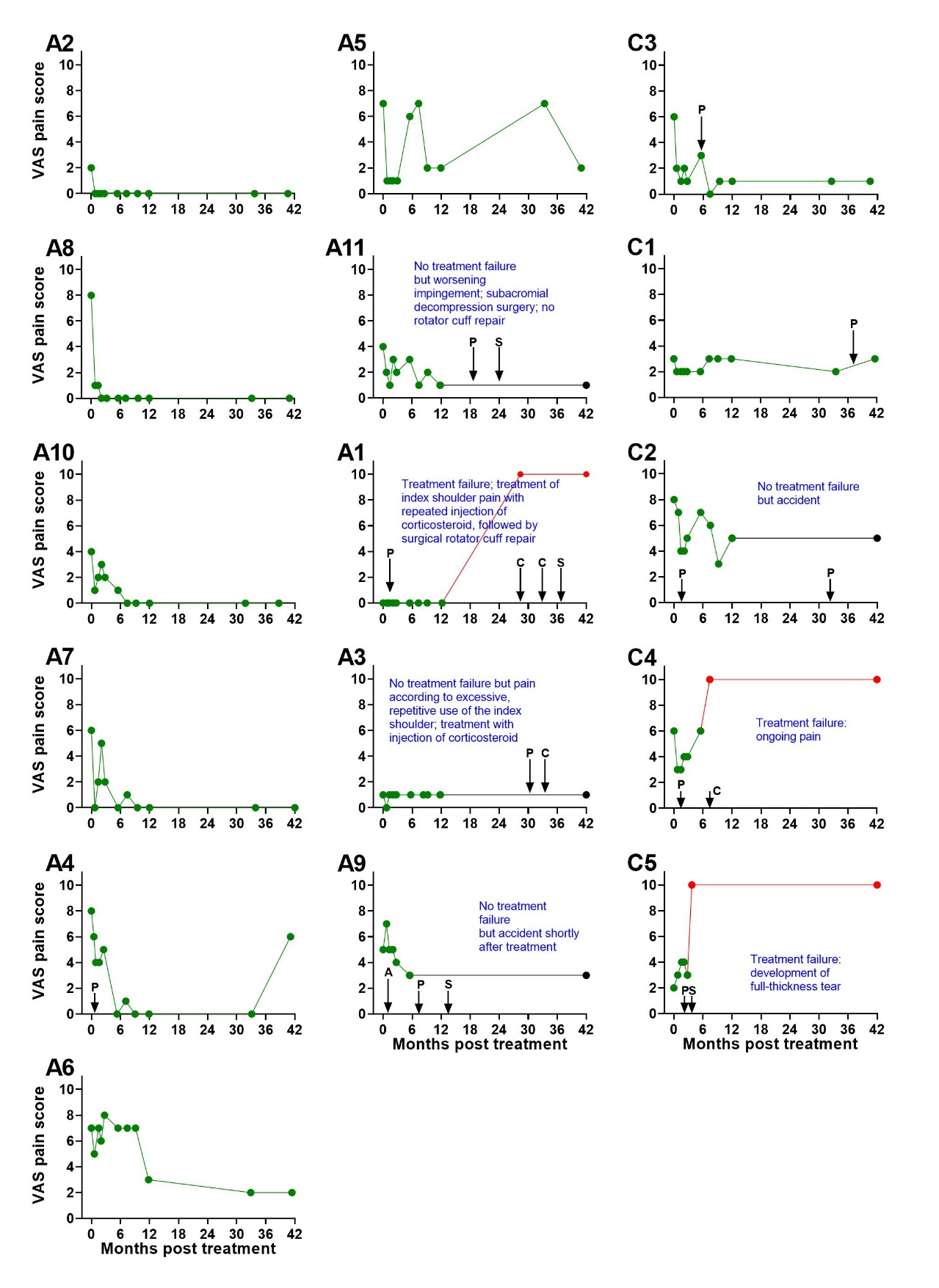


**Supplementary Table S16.** Mean, standard error of the mean and median of the ASES Total score collected during the present and the former studies after treating subjects suffering from sPTRCT with injection of either UA-ADRCs or corticosteroid. *sPTRCT* symptomatic, partial-thickness rotator cuff tear, *UA-ADRCs* fresh, uncultured, unmodified, autologous, adipose-derived regenerative cells, *D (U/C)* ratio of mean values between the subjects in the UA-ADRCs group and the subjects in the corticosteroid group, *p* result (p value) of Mann-Whitney test, *SEM* standard error of the mean, *BL* baseline, *W24 / W52* study visits scheduled in the former study at 24 and 52 weeks post-treatment, *FSV* first study visit of the present study, *SSV* second study visit of the present study.

| **Time point** | **Subjects treated with injection of UA-ADRCs** | | **Subjects treated with injection of corticosteroid** | | **D (U/C)** | **p** |
| --- | --- | --- | --- | --- | --- | --- |
|  | Mean ± SEM | Median | Mean ± SEM | Median |  |  |
| BL | 58.7 ± 5.8 | 56.7 | 50.6 ± 6.7 | 50 | +16.0% | 0.569 |
| W24 | 86.1 ± 4.9 | 95 | 48.0 ± 13.5 | 48.3 | +79.4% | 0.008 |
| W52 | 89.4 ± 4.9 | 98.3 | 43.0 ± 17.7 | 58.3 | +107.9% | 0.011 |
| FSV | 79.6 ± 9.9 | 98.3 | 46.0 ± 19.4 | 58.3 | +72.9% | 0.052 |
| SSV | 82.3 ± 9.4 | 98.3 | 44.3 ± 18.9 | 58.3 | +85.5 | 0.048 |

**Supplementary Table S17.** Mean, standard error of the mean and median of the SF-36 Total score collected during the present and the former studies after treating subjects suffering from sPTRCT with injection of either UA-ADRCs or corticosteroid. *sPTRCT* symptomatic, partial-thickness rotator cuff tear, *UA-ADRCs* fresh, uncultured, unmodified, autologous, adipose-derived regenerative cells, *D (U/C)* ratio of mean values of the subjects in the UA-ADRCs group and the subjects in the corticosteroid group, *p* result (p value) of Mann-Whitney test, *SEM* standard error of the mean, *BL* baseline, *W24 / W52* study visits scheduled in the former study at 24 and 52 weeks post treatment, *FSV* first study visit of the present study, *SSV* second study visit of the present study.

| **Time point** | **Subjects treated with injection of UA-ADRCs** | | **Subjects treated with injection of corticosteroid** | | **D (U/C)** | **p** |
| --- | --- | --- | --- | --- | --- | --- |
|  | Mean ± SEM | Median | Mean ± SEM | Median |  |  |
| BL | 557 ± 40.4 | 603.7 | 523 ± 40.4 | 560 | +6.5% | 0.320 |
| W24 | 696 ± 15.7 | 705.5 | 500 ± 127 | 606.5 | +39.1% | 0.013 |
| W52 | 654 ± 43.9 | 701.5 | 381 ± 166 | 422.7 | +71.5% | 0.307 |
| FSV | 602 ± 75.6 | 732 | 381 ± 166 | 422.7 | +58.0% | 0.220 |
| SSV | 582 ± 73.8 | 675.5 | 352 ± 152 | 422.7 | +65.4% | 0.179 |

**Supplementary Table S18.** Mean, standard error of the mean and median of the VAS Pain score (collected together with the ASES Total score) collected during the present and the former studies after treating subjects suffering from sPTRCT with injection of either UA-ADRCs or corticosteroid. *sPTRCT* symptomatic, partial-thickness rotator cuff tear, *UA-ADRCs* fresh, uncultured, unmodified, autologous, adipose-derived regenerative cells, *D (U/C)* ratio of mean values between the subjects in the UA-ADRCs group and the subjects in the corticosteroid group, *p* result (p value) of Mann-Whitney test, *SEM* standard error of the mean, *BL* baseline, *W24 / W52* study visits scheduled in the former study at 24 and 52 weeks post treatment, *FSV* first study visit of the present study, *SSV* second study visit of the present study.

| **Time point** | **Subjects treated with injection of UA-ADRCs** | | **Subjects treated with injection of corticosteroid** | | **D (U/C)** | **p** |
| --- | --- | --- | --- | --- | --- | --- |
|  | Mean ± SEM | Median | Mean ± SEM | Median |  |  |
| BL | 4.7 ± 0.8 | 5 | 5.0 ± 1.1 | 6 | -5.5% | 0.930 |
| W24 | 1.9 ± 0.8 | 1 | 5.6 ± 1.4 | 6 | -65.9% | 0.031 |
| W52 | 1.1 ± 0.5 | 0 | 5.8 ± 1.8 | 5 | -81.2% | 0.009 |
| FSV | 2.3 ± 1.1 | 0 | 5.6 ± 1.9 | 5 | -59.4% | 0.059 |
| SSV | 2.4 ± 1.0 | 1 | 5.8 ± 1.8 | 5 | -59.2% | 0.080 |

**Part 6 – Analysis of MRI scans**

**Supplementary Figure S21** (on the next page). Individual tear size (calculated as ellipsoid volume) as a function of time post-treatment of subjects treated with injection of either UA-ADRCs (Subjects A1-A11) or corticosteroid (Subjects C1-C5). The data are arranged in descending order of individual treatment success (i.e., the data of the subjects with the hightest ASES Total scores are shown in the top row of the left column (Subject A2 treated with injection of corticosteroid) and the right column (Subject C3 treated with injection of UA-ADRCs) (c.f. Supplementary Fig. S18). In the graphs (i) green dots and green lines indicate data of the present and the former studies that were available and could be used to assess treatment outcome, (ii) black dots and black lines (Subjects A11, A13 and C2) indicate data of the present study that were imputed uisng the Last Observation Carried Forward approach and could be used to assess treatment outcome (reasons are indicated in the corresponding panels and outlined in detail in SupplementaryTable S15), and (iii) red dots and red lines (Subjects A1, C4 and C5) indicate data of the present and the former studies that were imputed as "failures" (reasons are indicated in the corresponding panels and outlined in detail in Supplementary Table S15). *A* accident affecting the index shoulder, *C* injection of corticosteroid into the index shoulder, *P* pain in the index shoulder, *S* surgery of the index shoulder.

**Supplementary Figure S21 (cont.)**


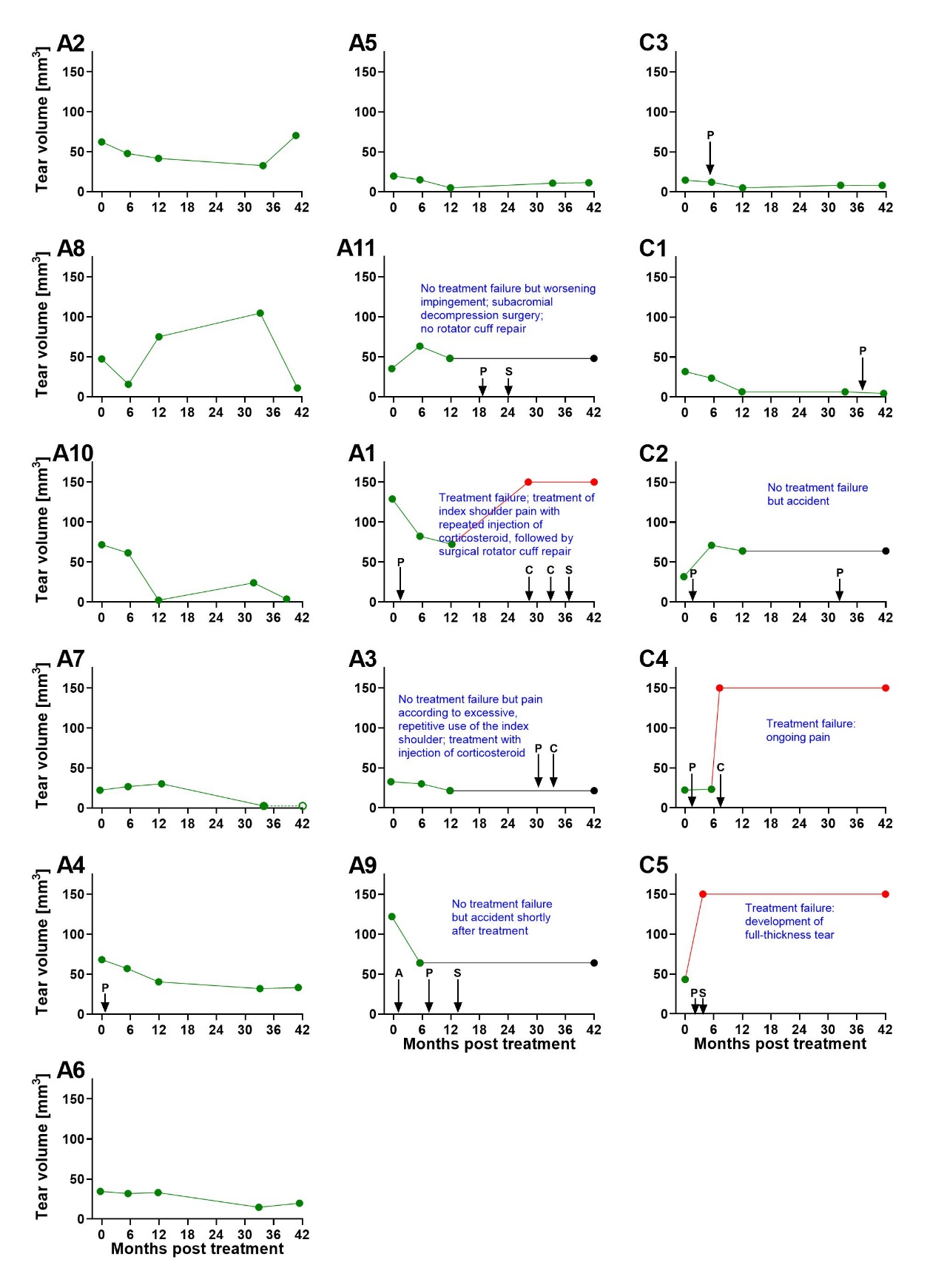


**Supplementary Table S19.** Mean, standard error of the mean and median of the tear size (calculated as ellipsoid volume) collected during the present and the former studies after treating subjects suffering from sPTRCT with injection of either UA-ADRCs or corticosteroid. *sPTRCT* symptomatic, partial-thickness rotator cuff tear, *UA-ADRCs* fresh, uncultured, unmodified, autologous, adipose-derived regenerative cells, *D (U/C)* ratio of mean values of subjects in the UA-ADRCs group and subjects in the corticosteroid group, *p* result (p value) of Mann-Whitney test, *SEM* standard error of the mean, *BL* baseline, *W24 / W52* study visits scheduled in the former study at 24 and 52 weeks post treatment, *FSV* first study visit of the present study, *SSV* second study visit of the present study.

| **Time point** | **Subjects treated with injection of UA-ADRCs** | | **Subjects treated with injection of corticosteroid** | | **D (U/C)** | **p** |
| --- | --- | --- | --- | --- | --- | --- |
|  | Mean ± SEM [mm^3^] | Median [mm^3^] | Mean ± SEM [mm^3^] | Median [mm^3^] |  |  |
| BL | 58.6 ± 11.3 | 47.3 | 28.7 ± 4.8 | 31.7 | +104% | 0.055 |
| W24 | 45.0 ± 6.8 | 47.7 | 55.9 ± 25.6 | 23.5 | -19.6% | 0.827 |
| W52 | 44.5 ± 10.3 | 40.5 | 75.1 ± 32.4 | 63.9 | -40.7% | 0.564 |
| FSV | 55.7 ± 14.9 | 32.6 | 75.7 ± 32.1 | 63.9 | -26.5% | 0.827 |
| SSV | 49.3 ± 14.9 | 33.3 | 75.3 ± 32.3 | 63.9 | -34.5% | 0.660 |

**Part 7 – Relationship between treatment outcome and baseline data**

**Supplementary Figure S22** (on the next pages). Individual ASES Total score as a function of time post-treatment, and individual data at baseline (ASES Total score, tear volume, age, body mass index, cell yield and cell viability) of the subjects who were treated with injection of UA-ADRCs (Subjects A1-A11). The data are arranged in descending order of individual treatment success (i.e., the data of the subject with the best treatment outcome (Subject A2) are shown in the top row). In the graphs showing individual ASES Total scores as a function of time post-treatment (i) green dots and green lines (Subjects A2, A4, A5, A6, A7, A8 and A10) indicate that all data of the present and the former studies were available and could be used for assessing treatment outcome, (ii) black dots and black lines (Subjects A1, A3 and A11) indicate that all data of the former study were available and could be used for assessing treatment outcome, but in the time period between the present and the former studies there was an incidence that rendered the data of the present study unsuitable for assessing treatment outcome (reasons are indicated in the corresponding panels and outlined in detail in Supplementary Table S15), and (iii) red dots and red lines (Subject A9) indicate that there was an incidence during the former study that rendered a part of the data of the former study (and, thus, all data of the present study in case they were collected) unsuitable for assessing treatment outcome (reasons are indicated in the corresponding panel and outlined in detail in Supplementary Table S15). Furthermore, in the graphs showing individual data at baseline (ASES Total score, tear volume, age, body mass index, cell yield and cell viability) (i) the individual data of the subject whose ASES Total score as a function of time post-treatment is shown in the same row are given on the left ("Subject"), and (ii) the data of all subjects in the corticosteroid group are given on the right ("All"). The horizontal lines represent mean values. In addition, for each variable the subject treated with UA-ADRCs with (i) presumably the best prognosis (at baseline highest ASES Total score, smallest tear volume, youngest age and lowest body mass index, as well as highest cell yield and highest cell viability) is indicated with a green dot and green frame surrounding the corresponding data, and (ii) presumably the worst prognonis (at baseline lowest ASES Total score, largest tear volume, oldest age and highest body mass index, as well as lowest cell yield and lowest cell viability) is indicated with a red dot and red frame surrounding the corresponding data. *A* accident affecting the index shoulder, *C* injection of corticosteroid into the index shoulder, *P* pain in the index shoulder, *S* surgery of the index shoulder.

**Supplemental Figure S22 (cont.)**


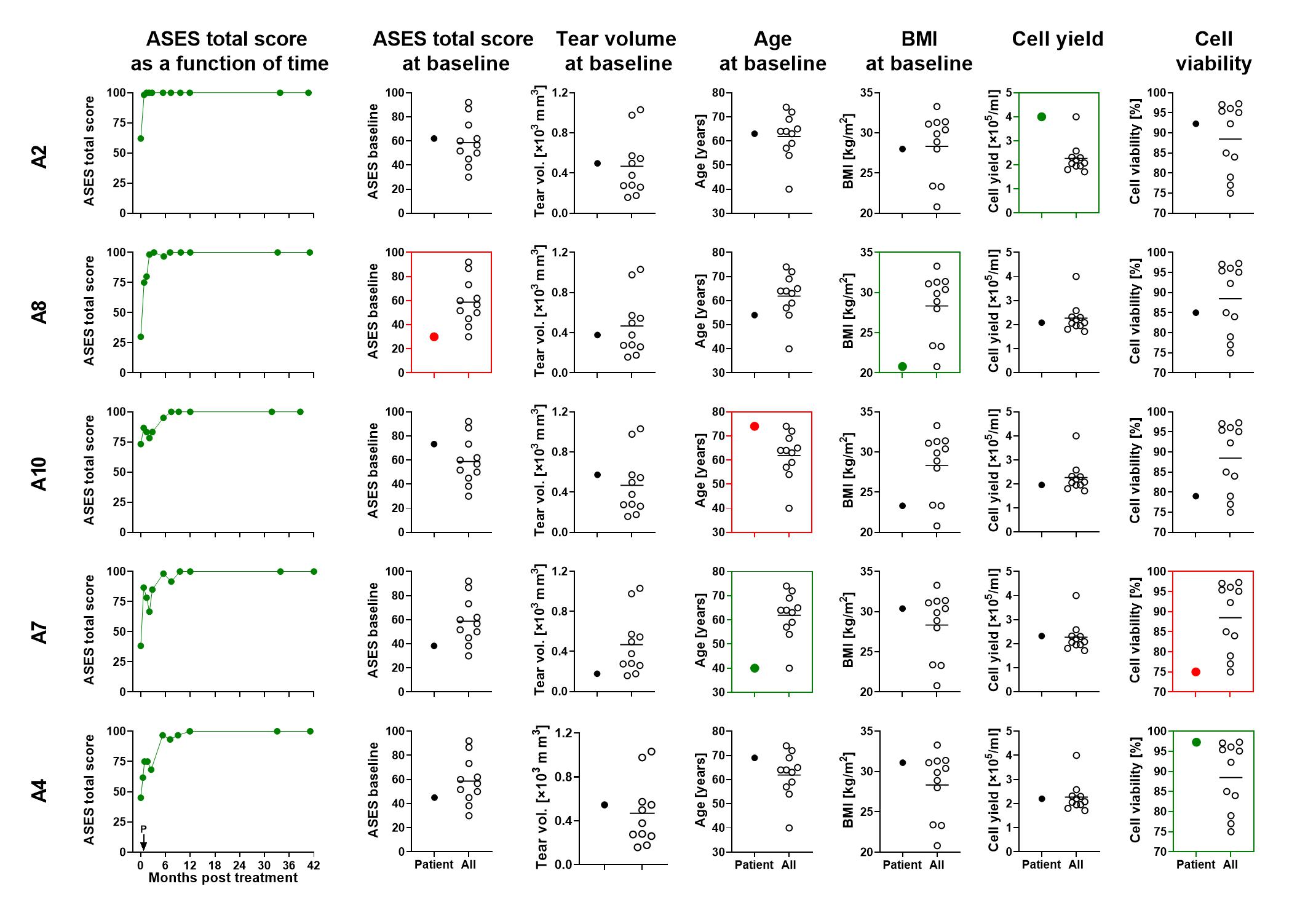


**Supplemental Figure S22 (cont.)**


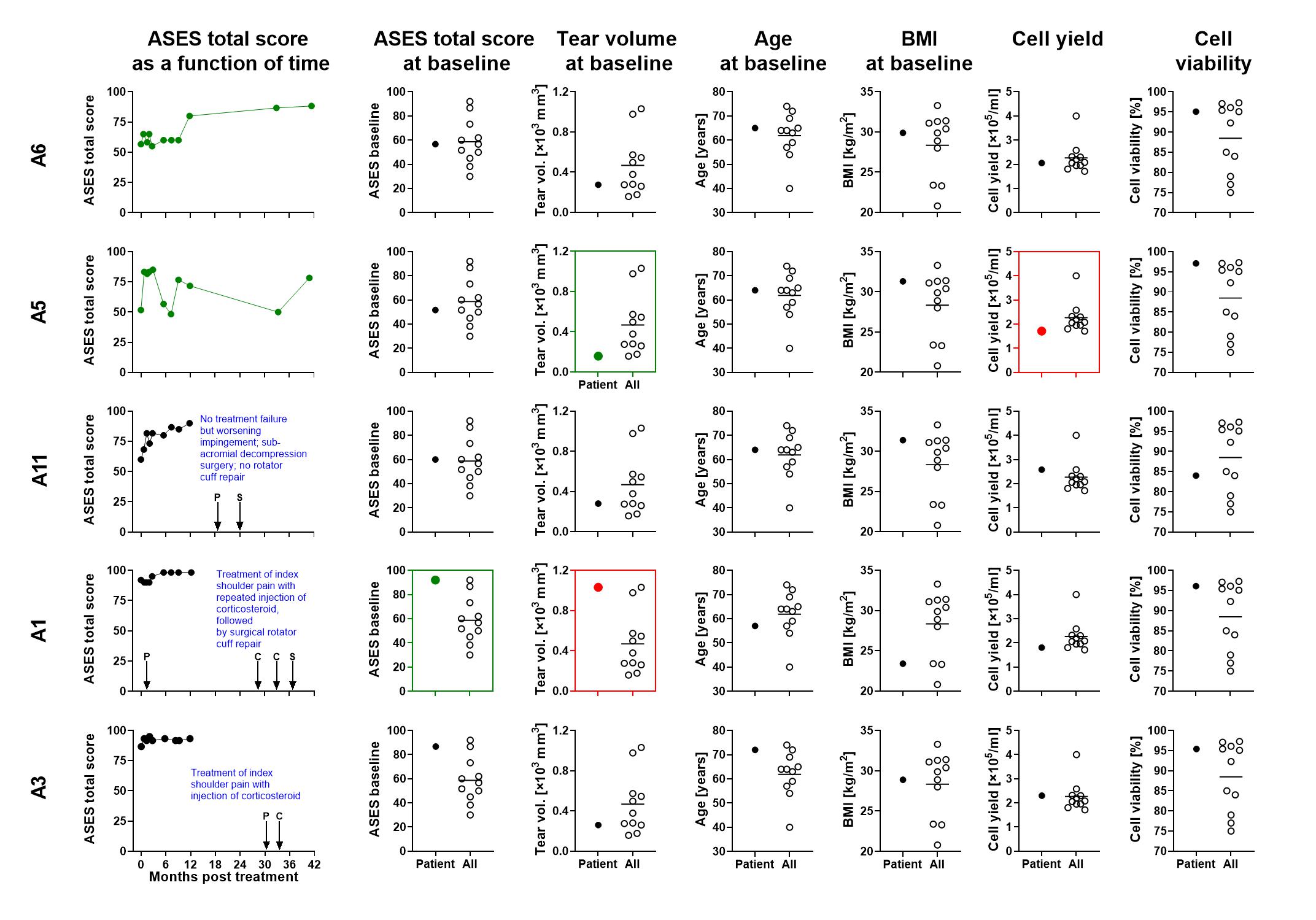


**Supplemental Figure S22 (cont.)**


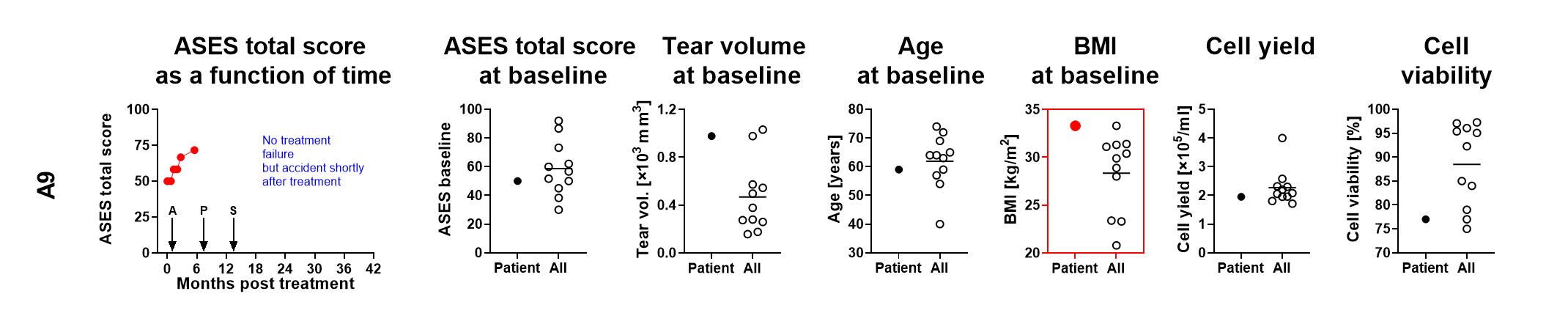


**Supplementary Figure S23** (on the next page). Individual ASES Total score as a function of time post-treatment, and individual data at baseline (ASES Total score, tear volume, age and body mass index) of the subjects who were treated with corticosteroid. The data are arranged in descending order of individual treatment success (i.e., the data of the subject with the best treatment outcome (C3) are shown in the top row). In the graphs showing individual ASES Total scores as a function of time post-treatment (i) green dots and green lines (Subjects A2, A4, A5, A6, A7, A8 and A10) indicate that all data of the present and the former studies were available and could be used for assessing treatment outcome, (ii) black dots and black lines (Subjects A1, A3 and A11) indicate that all data of the former study were available and could be used for assessing treatment outcome, but in the time period between the present and the former studies there was an incidence that rendered the data of the present study unsuitable for assessing treatment outcome (reasons are indicated in the corresponding panels and outlined in detail in Supplementary Table S15), and (iii) red dots and red lines (Subject A9) indicate that there was an incidence during the former study that rendered a part of the data of the former study (and, thus, all data of the present study in case they were collected) unsuitable for assessing treatment outcome (reasons are indicated in the corresponding panel and outlined in detail in Supplementary Table S15). Furthermore, in the graphs showing individual data at baseline (ASES Total score, tear volume, age and body mass index) (i) the individual data of the subject whose ASES Total score as a function of time post-treatment is shown in the same row are given on the left ("Subject"), and (ii) the data of all subjects in the corticosteroid group are given on the right ("All"). The horizontal lines represent mean values. In addition, for each variable the subject treated with corticosteroid with (i) presumably the best prognosis (at baseline highest ASES Total score, smallest tear volume, youngest age and lowest body mass index) is indicated with a green dot and green frame surrounding the corresponding data, and (ii) presumably the worst prognonis (at baseline lowest ASES Total score, largest tear volume, oldest age and highest body mass index) is indicated with a red dot and red frame surrounding the corresponding data (note that this was not possible in case of the BMI at baseline because for Subject C5 no height was collected in the former study and, thus, no BMI could be calculated). *P* pain in the index shoulder, *C* injection of corticosteroid into the index shoulder, *S* surgery of the index shoulder.

**Supplementary Figure S23 (cont.)**


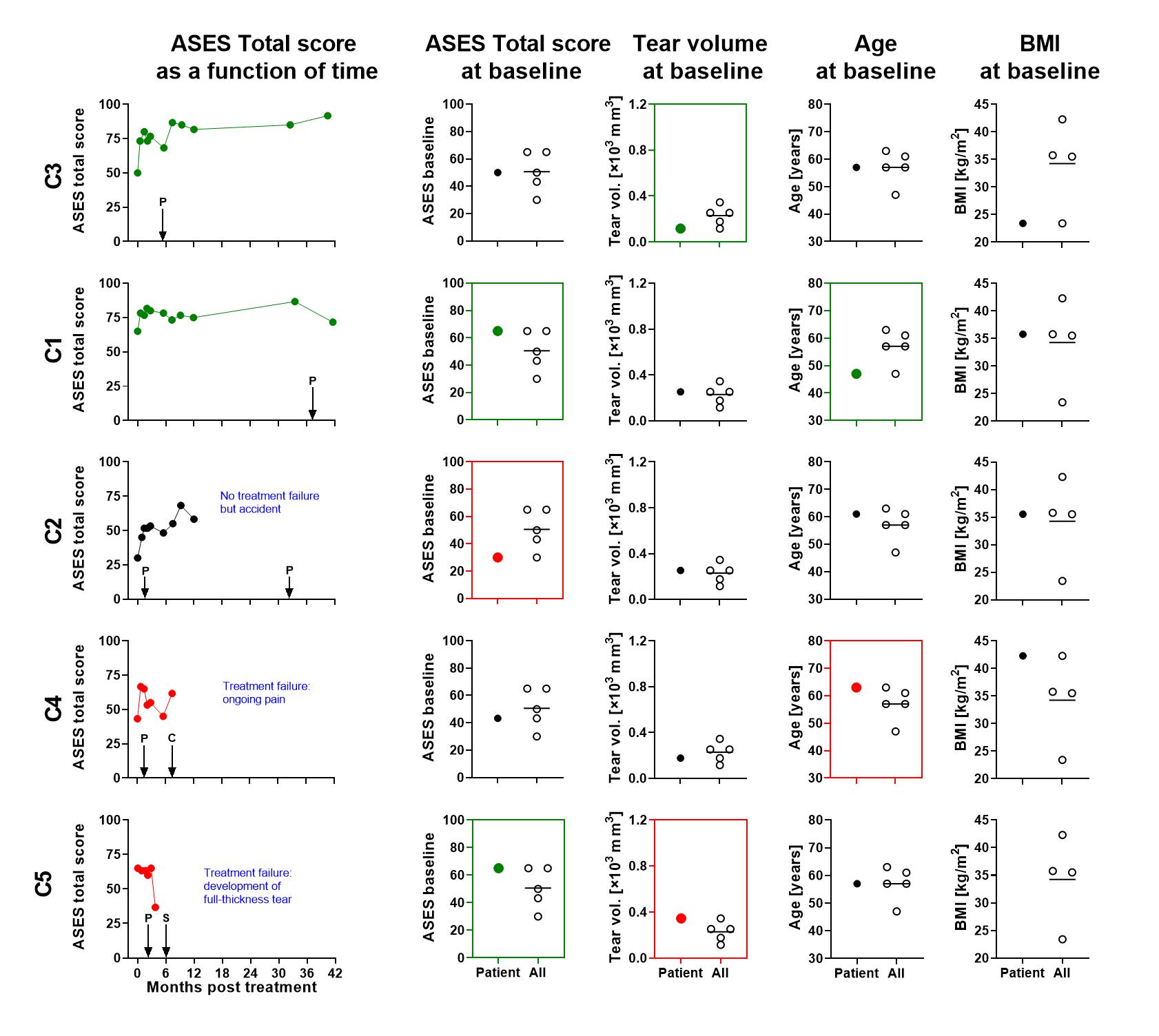


**Part 8 – Comparison of the results obtained in the present and the former studies after treatment of sPTRCT with injection of corticosteroid with corresponding results of other studies in the literature**

**Supplementary Table S20.** Details of published studies that investigated the efficacy of treating partial-thickness rotator cuff tears with injections of corticosteroids. References are in the main text. *HE* hydrocortisone equivalent (https://clincalc.com/Corticosteroids/), *HED* hydrocortisone equivalent dose [mg], *^a^* incomplete description of dosage ("patients received a cortisone injection [Dermapharm Kenacort^®^ 40 mg (triamcinolone acetonide, crystal suspension)] by means of a 5-ml syringe under aseptic conditions.").

| **Study** | **Treatment** | **HE** | **HED** | **Additional injection of local anesthetic** |
| --- | --- | --- | --- | --- |
| [12] | 2 mL 40 mg/ml methylprednisolone | 5 | 1 x 400 | 3 mL of 0.25% bupivacaine |
| [41] | 1 ml of 6 mg betamethasone | 25 | 1 x 150 | 4 mL of 2% xylocaine |
| [42] | 5 ml of 40 mg/ml triamcinolone acetonide* | 5 | 1 x 1000 (?)**^a^** | -- |
| [43] | 1mL of 40 mg/mL methylprednisolone acetate | 5 | 1 x 200 | 1mL of 1% lignocaine hydrochloride |
| [44] | 7 mg betamethasone (3x, 1x/week) | 25 | 3 x 175 | -- |
| [45] | 2 mL of 40 mg/ml triamcinolone acetonide | 5 | 1 x 400 | 2 mL of 1% lidocaine |

**Supplementary Table S21.** Results of the studies summarized in Supplementary Table S20. References are in the main text. *BL* baseline, *M6* six months post baseline, *n* number of subjects investigated, *SD* standard deviation, *Sig* level of significance (p values), *--* not reported, *^a^* pain with Neer impingement sign, *^b^* scored on a five-point Likert scale (very bad, bad, poor, fair, good) and converted to a numerical score from 0 to 4, *^c^* median data.

| **Study** | **VAS pain score** | | | | | | **ASES total score** | | | | | |
| --- | --- | --- | --- | --- | --- | --- | --- | --- | --- | --- | --- | --- |
|  |  | **BL** | | **M6** | |  |  | **BL** | | **M6** | |  |
|  | **n** | **Mean** | **SD** | **Mean** | **SD** | **Sig** | **n** | **Mean** | **SD** | **Mean** | **SD** | **Sig** |
| [12] | 5 | 41.2 | 24.7 | 39.0 | 29.4 | >0.05 | 5 | 50.6 | 15.0 | 64.8 | 12.5 | <0.05 |
| [41] | 30 | 54.8**^a^** | 24 | 45.2 | 27.7 | -- | 30 | 46.9 | 18.3 | 62.3 | 22.9 | -- |
| [42] | 25 | -- | -- | -- | -- | -- | 25 | 50.6 | 14 | 77.1 | -- | <0.01 |
| [43] | 19 | 1.8**^b^** | 0.2 | 2.4**^b^** | 0.3 | 0.24 | -- | -- | -- | -- | -- | -- |
| [44] | 16 | 65**^c^** | -- | 40**^c^** | -- | -- | -- | -- | -- | -- | -- | -- |
| [45] | 33 | 56.3 | 9.3 | 37.7 | 14.1 | <0.001 | 33 | 40.13 | 8.18 | 55.63 | 11 | <0.001 |

**Part 9 – Estimand of the present study according to *The International Council for Harmonisation of Technical Requirements for Pharmaceuticals for Human Use (ICH) E9 (R1) Addendum***

**1. Population**

The population of the present study comprised subjects who fulfilled the following inclusion criteria and did not fulfill any of the following exclusion criteria:

**1.1 Inclusion criteria of the present study**

1. Subject completed participation in RC-001 (NCT02918136) study.
2. Must have the ability to understand and sign a written informed consent form (ICF), which must be obtained prior to initiation of study procedures associated with this trial.

**1.2 Exclusion criteria of the present study**

1. None.

**1.3 Inclusion criteria of the former study**

1. Males and females 30-75 years of age.
2. Clinical symptoms consistent with a rotator cuff lesion including but not limited to pain, muscle weakness, or active-limited range of motion (AROM).
3. Subjects who have not responded to physical therapy treatments for at least six weeks.
4. Subjects with >70% passive range of motion (PROM).
5. Diagnosed with >50% tear to supraspinatus muscle or < 5mm separation assessed by MRI.
6. Diagnosed with a partial-thickness rotator cuff tear.
7. The ability of subjects to give appropriate consent.

**1.2 Exclusion criteria of the former study**

1. Age <30 or >75.
2. Diagnosed with a full-thickness rotator cuff tear.
3. Insufficient amount of subcutaneous tissue to allow recovery of 50 mL of lipoaspirate.
4. History of systemic malignant neoplasms within last 5 years.
5. History of local neoplasm within the last 6 months and any history of local neoplasm at site of administration.
6. Subject is receiving immunosuppressant therapy or has known immunosuppressive or severe autoimmune disease that requires chronic immunosuppressive therapy (e.g., human immunodeficiency virus, systemic lupus erythematosus, etc.).
7. Subjects who are known to be HIV positive.
8. Patients who have received a corticosteroid injection in rotator cuff site within last 3 months.
9. Severe arthrosis of the glenohumeral or acromioclavicular joint.
10. Irreparable rotator cuff tear (including rotator cuff tear arthropathy).
11. Fatty atrophy above Grade 2 in affected shoulder.
12. Previous shoulder surgeries in affected shoulder.
13. Any contraindication to MRI scan according to MRI guidelines, or unwillingness to undergo MRI procedures.
14. History of tobacco use within the last 3 months.
15. Patient is on an active regimen of chemotherapy.
16. Patients with a documented history of liver disease or an ALT value >400.
17. Allergy to sodium citrate of any “caine” type of local anesthetic.
18. Patient is pregnant or breast feeding.
19. Subject is, in the opinion of the investigator or designee, unable to comply with the requirements of the study protocol or is unsuitable for the study for any reason. This includes completion of Patient Reported Outcome instruments.
20. Subject is currently participating in another clinical trial that has not yet completed its primary endpoint.
21. Subject is part of a vulnerable population who, in the judgment of the investigator, is unable to give Informed Consent for reasons of incapacity, immaturity, adverse personal circumstances or lack of autonomy. This may include: individuals with mental disability, persons in nursing homes, children, impoverished persons, persons in emergency situations, homeless persons, nomads, refugees, and those incapable of giving informed consent. Vulnerable populations also may include members of a group with a hierarchical structure such as university students, subordinate hospital and laboratory personnel, employees of the sponsor, members of the armed forces, and persons kept in detention.
22. Uncooperative patients or those with neurological/psychiatric disorders who are incapable of following directions or who are predictably unwilling to return for follow-up examinations.

Note: the criteria 20-23 were related to the former study (and, thus, also to the present study) and may not apply in management of sPTRCT using UA-ADRCs generated by the Transpose RT System (InGeneron Inc., Houston, TX, USA).

**2. Variables**

The variables of the present study were the following:

**2.1 Variables evaluating long-term effectiveness:**

- ASES Total score (used in the primary endpoints)
- SF-36 Total score (used in the primary endpoints)
- Magnetic resonance imaging (used as secondary endpoint)
- VAS – Pain score (collected together with the ASES Total score)

**2.2 Variables evaluating long-term safety:**

- Adverse event rate between the UA-ADRCs group and the corticosteroid group

**3. Intercurrent Events**

The possible intercurrent events of the present and the former studies comprised

- adverse events,
- use of concomitant medication,
- single missed study visits,
- loss to follow-up,
- additional assessments outside the visit window,
- discontinuation by investigator, and
- discontinuation by subject.

These possible intercurrent events, strategies to handle them according to ICH E9(R1) and imputation of possible subjects' missing data related to each type of intercurrent event are described in detail in the following.

**3.1 Adverse events**

Since the protocol of the present study specified group-specific comparisons of adverse events for the time periods (i) from BL to W24 post-treatment (considering only data of the former study, (ii) from BL to FSV in the present study, and (iii) from BL to SSV in the present study (each considering data of the present and the former studies), the following description of the different types of adverse events applies to the present and the former studies.

**3.1.1 Anticipated adverse events**

This type of intercurrent event did not comprise serious adverse events, because no serious adverse events were anticipated in the present and the former studies.

This type of intercurrent event comprised fever, bleeding, bruising, persistent swelling at injection site, tenderness at injection site, pain at injection site, infection at injection site, redness or swelling at injection site, lightening of the skin around the injection site, joint infection, inflammatory flare, thinning of the skin and soft tissue around the injection site, tendon weakening, shoulder pain, worsening shoulder pain, nerve damage, death of nearby bone, calcium deposits on the tendon site, death of cartilage, potential allergic reactions (including anaphylaxis), prolonged numbness, tingling, a feeling of “pins and needles”, temporary skin, discoloration, itching or swelling where the medication was injected, the feeling of anxiousness, shakiness, dizziness, restlessness, or depression, drowsiness, vomiting and nausea.

This type of intercurrent event could occur in the UA-ADRCs group and the corticosteroid group, and the intercurrent events could or could not lead to subjects' missing data.

**3.1.1.1 Anticipated adverse events that were possibly, probably or definitely related to the study treatments or to the liposuction procedure, were temporary, and were not serious adverse events**

Anticipated adverse events could have possibly, probably or definitely been related to the study treatments or to the liposuction procedure. It was expected that most of these anticipated adverse events are temporary intercurrent events, and are not serious adverse events. These intercurrent events were expected to also occur in clinical use of UA-ADRCs generated by the Transpose RT System (InGeneron) or in clinical use of corticosteroid, and as such were handled using the Treatment Policy, i.e., whether this type of intercurrent event had occurred or not was irrelevant, and the data were collected and analyzed regardless. Possible subjects' missing data related to this type of intercurrent event were imputed using the Last Observation Carried Forward approach.

This type of intercurrent event did occur in the present and the former studies (Tables S1 and S2).

**3.1.1.2 Anticipated adverse events at the index shoulder that were possibly, probably or definitely related to the study treatments and required additional injections into the index shoulder**

If any of the anticipated adverse events *tendon weakening* (of a tendon in the index shoulder), *shoulder pain* (of the index shoulder), *worsening shoulder pain* (of the index shoulder), *calcium deposits on the tendon site* (of a tendon in the index shoulder), *death of nearby bone* (at the index shoulder) and *death of cartilage* (at the index shoulder) were possibly, probably or definitely related to the study treatments and required additional injections into the index shoulder after study treatment (e.g., injection of corticosteroid as additional treatment during the follow-up period, regardless of the study treatment), they were considered representing treatment failure. In this case these intercurrent events were handled using a combination of the While-on-Treatment Strategy and the Composite Strategy. Specifically, response to study treatment before the occurrence of the intercurrent event was handled using the While-on-Treatment Strategy, whereas response to study treatment after the occurrence of the intercurrent event was imputed according to the Composite Variable Strategy as minimum ASES Total score (0), minimum SF-36 Total score (0), maximum VAS pain score (10) and maximum tear volume measured on MRIs (150 mm^3^, which was greater than all data measured during the present and the former studies). Accordingly, after occurrence of these intercurrent events subjects' missing data related to this type of intercurrent event were imputed as “failures” (i.e. non-responders).

This type of intercurrent event did occur in the present and the former studies (Tables S2 and S15).

**3.1.2 Unanticipated adverse events**

This type of intercurrent event comprised non-serious adverse events and serious adverse events (note that no serious adverse events were anticipated in the present and the former studies).

This type of intercurrent event comprised the death of a subject, a life-threatening illness or injury, a permanent impairment of a body structure or a body function, hospitalization or prolonged existing hospitalization, or an important medical event defined as an event requiring medical or surgical intervention to prevent one of the outcomes listed above in this definition.

Although unanticipated, this type of intercurrent event could occur in the UA-ADRCs group and the corticosteroid group, and the intercurrent events could or could not lead to subjects' missing data. Possible subjects' missing data related to this type of intercurrent event were imputed using the Last Observation Carried Forward approach.

**3.1.2.1 Death of a subject in the UA-ADRCs group** **that would possibly, probably or definitely have been related to the study treatment (i.e., to the injection of UA-ADRCs)**

In the extremely unlikely event of the death of a subject in the UA-ADRCs group that would possibly, probably or definitely have been related to the study treatment (i.e., to the injection of UA-ADRCs), it was planned to handle response to study treatment before the occurrence of the intercurrent event according to the While-on-Treatment Strategy, and to prematurely discontinue the study because evidence would have emerged that would have made the study unethical. In this case subjects' missing data related to this type of intercurrent event would have been imputed as “failures” (i.e. non-responders).

This type of intercurrent event **did not occur** in the present and the former studies.

**3.1.2.2 Death of a subject in the UA-ADRCs group or the corticosteroid group** **that would possibly, probably or definitely have been related to the liposuction procedure**

In the extremely unlikely event of the death of a subject in the UA-ADRCs group that would have been possibly, probably or definitely related to the liposuction procedure, it was planned to handle response to study treatment before the occurrence of the intercurrent event according to the While-on-Treatment Strategy, and to prematurely discontinue the study because evidence would have emerged that would have made the study unethical. In this case subjects' missing data related to this type of intercurrent event would have been imputed as “failures” (i.e. non-responders).

This type of intercurrent event **did not occur** in the present and the former studies.

**3.1.2.3 Death of a subject in the corticosteroid group** **that would possibly, probably or definitely have been related to the study treatment (i.e., to the injection of corticosteroid)**

In the extremely unlikely event of the death of a subject in the corticosteroid group that would possibly, probably or definitely have been related to the study treatment (i.e., to the injection of corticosteroid), it was planned to handle response to study treatment before the occurrence of the intercurrent event according to the While-on-Treatment Strategy. Subjects' missing data related to this type of intercurrent event would have been imputed as “failures” (i.e. non-responders).

This type of intercurrent event **did not occur** in the present and the former studies.

**3.1.2.4 Death of a subject in the UA-ADRCs group or the corticosteroid group that would have been unlikely related or unrelated to the study treatments or the liposuction procedure (e.g., infection with SARS-CoV2 resulting in subject's death), or whose causality could not have been determined**

In the event of the death of a subject in the UA-ADRCs group or the corticosteroid group that would have been unlikely related or unrelated to the study treatments or the liposuction procedure (e.g., infection with SARS-CoV2 resulting in subject's death), or whose causality could not have been determined, it was planned to handle response to study treatment before the occurrence of the intercurrent event according to the While-on-Treatment Strategy. Subjects' missing data related to this type of intercurrent event would have been imputed using their last available observation.

This type of intercurrent event **did not occur** in the present and the former studies.

**3.1.2.5 Unanticipated, serious adverse events that were possibly, probably or definitely related to the study treatments, required surgical intervention, and were not joint infection**

If any of the anticipated adverse events *tendon weakening* (of a tendon in the index shoulder), *shoulder pain* (of the index shoulder), *worsening shoulder pain* (of the index shoulder), *calcium deposits on the tendon site* (of a tendon in the index shoulder), *death of nearby bone* (at the index shoulder) and *death of cartilage* (at the index shoulder) were possibly, probably or definitely related to the study treatments and required surgery of the index shoulder after study treatment (i.e., during the follow-up period of the study), they were considered representing treatment failure.

In this case these intercurrent events were handled using a combination of the While-on-Treatment Strategy and the Composite Strategy. Specifically, response to study treatment before the occurrence of the intercurrent event was handled using the While-on-Treatment Strategy, whereas response to study treatment after the occurrence of the intercurrent event was imputed according to the Composite Variable Strategy as minimum ASES Total score (0), minimum SF-36 Total score (0), maximum VAS pain score (10) and maximum tear volume measured on MRIs (150 mm^3^, which was greater than all data measured during the present and the former studies). Accordingly, after occurrence of these intercurrent events subjects' missing data related to this type of intercurrent event were imputed as “failures” (i.e. non-responders).

This type of intercurrent event did occur in the present and the former studies (Tables S1, S2 and S15).

**3.1.2.6 Joint infections of the index shoulder that were possibly, probably or definitely related to the study treatments and require surgical intervention**

If the anticipated adverse event *joint infection* (of the index shoulder) would have required surgery of the index shoulder after study treatment, it would have been necessary to rate this type of intercurrent event as serious adverse events. However, it was not anticipated that such intercurrent events would occur in the present and the former studies.

In case such an intercurrent event would have occured in the present and the former studies, response to study treatment before the occurrence of the intercurrent event would have been handled according to the While-on-Treatment Strategy, whereas response to study treatment after the occurrence of the intercurrent event would have been imputed according to a Hypothetical Strategy in which the intercurrent event would not have occured (because the intercurrent event would have been related to the injection procedure but not to the study treatment itself). Possible subjects' missing data related to this type of intercurrent event would have been imputed using the Last Observation Carried Forward approach.

This type of intercurrent event **did not occur** in the present and the former studies.

**3.1.2.7 Unanticipated serious adverse events that were possibly, probably or definitely related to the study treatments or the liposuction procedure, but were not related to the index shoulder**

All available (published and unpublished) data indicated that this type of intercurrent event would not occur in the present and the former studies.

In the unlikely event that this type of intercurrent event would have nevertheless occurred in the UA-ADRCs group or the corticosteroid group (with the possibility of subjects' missing data) during the present and the former studies, response to study treatment before and after the occurrence of the intercurrent event would have been handled according to Treatment Policy, i.e., whether this type of intercurrent event would have occurred or not is irrelevant, and the data would have been collected and analyzed regardless. Possible subjects' missing data related to this type of intercurrent event would have been imputed using the Last Observation Carried Forward approach.

This type of intercurrent event **did not occur** in the present and the former studies.

**3.1.2.8 Adverse events and serious adverse events that were unlikely related or unrelated to the study treatments or the liposuction procedure, or whose causality could not be determined, were related to the index shoulder (e.g., accidents involving the index shoulder) but did not result in the subjects' death**

This type of intercurrent event could occur in the UA-ADRCs group and the corticosteroid group, and the intercurrent events could or could not lead to subjects' missing data.

In case this type of intercurrent event occured in the present and the former studies, it was handled using a combination of the While-on-Treatment Strategy and a Hyopthetical Strategy. Specifically, response to the study treatment before the occurrence of the intercurrent event was handled according to the While-on-Treatment Strategy, whereas response to the study treatment after the occurrence of the intercurrent event was imputed according to a Hypothetical Strategy in which the intercurrent event would not occur. Possible subjects' missing data related to this type of intercurrent event was imputed using the Last Observation Carried Forward approach.

This type of intercurrent event did occur in the present and the former studies (Tables S1, S2 and S15).

**3.1.2.9 Non-serious adverse events that were unlikely related or unrelated to the study treatments or the liposuction procedure, or whose causality could not be determined, and were not related to the index shoulder (e.g., infection with SARS-CoV-2 that did not require hospitalization)**

This type of intercurrent event could occur in the UA-ADRCs group and the corticosteroid group, and the intercurrent events could or could not lead to subjects' missing data.

In case this type of intercurrent event occured in the present and the former studies, it was handled according to Treatment Policy, i.e., whether this type of intercurrent event had occurred or not was irrelevant, and the data were collected and analyzed regardless. Possible subjects' missing data related to this type of intercurrent event were imputed using the Last Observation Carried Forward approach.

This type of intercurrent event did occur in the present and the former studies (Tables S1 and S2).

**3.1.2.10 Serious adverse events that would have been unlikely related or unrelated to the study treatments or the liposuction procedure, or whose causality could not have been determined, would not have been related to the index shoulder, and would have made collection of study data temporarily impossible (e.g., infection with SARS-CoV-2 that would have required hospitalization or car accidents)**

This type of intercurrent event could have occured in the UA-ADRCs group and the corticosteroid group, and the intercurrent events could or could not have led to subjects' missing data.

In case this type of intercurrent event would have occured in the study, it would have been handled using a combination of the While-on-Treatment Strategy and a Hyopthetical Strategy. Specifically, response to the study treatment before the occurrence of the intercurrent event would have been handled according to the While-on-Treatment Strategy, whereas response to the study treatment after the occurrence of the intercurrent event would have been imputed according to a Hypothetical Strategy in which the intercurrent event would not have occured. Possible subjects' missing data related to this type of intercurrent event would have been imputed using the Last Observation Carried Forward approach.

This type of intercurrent event **did not occur** in the present and the former studies.

**3.2 Use of concomitant medication (except for injections into the index shoulder after randomization that did not represent study treatments)**

This type of intercurrent event could occur in the UA-ADRCs group and the corticosteroid group.

In case this type of intercurrent event occured during the present and the former studies, response to study treatment before and after the occurrence of the intercurrent event was handled according to Treatment Policy, i.e., the data was collected and analyzed regardless.

This type of intercurrent event did occur in the present and the former studies (data not shown).

**3.3 Single missed study visits**

This type of intercurrent event could have occured in the UA-ADRCs group and the corticosteroid group, and the intercurrent events could have led to subjects' missing data.

In case this type of intercurrent event would have occurred during the present and the former studies, response to study treatment before and after the occurrence of the intercurrent event would have been handled according to a Hypothetical Strategy in which the intercurrent event would not have occured. Subjects' missing data related to this type of intercurrent event would have been imputed using the Last Observation Carried Forward approach.

This type of intercurrent event **did not occur** in the present and the former studies.

**3.4 Loss to Follow-up**

Loss to follow-up would have occured when a subject would have missed two consecutive, scheduled follow-up time points (study visits). If attempts to contact the subject or subject’s healthcare provider would have been unsuccessful, then the subject would have been considered lost-to-follow-up.

This type of intercurrent event could have occured in the UA-ADRCs group and the corticosteroid group, and the intercurrent events could have led to subjects' missing data.

In case this type of intercurrent event would have occurred during the present and the former studies, we would have determined whether loss to follow-up is related to any of the types of intercurrent events outlined in this section, and would have applied the corresponding strategy for addressing the intercurrent event and adequate imputation of subjects' missing data. Otherwise this type of intercurrent event would have been handled using a combination of the While-on-Treatment Strategy and a Hyopthetical Strategy. Specifically, response to the study treatment before the occurrence of the intercurrent event would have been handled according to the While-on-Treatment Strategy, whereas response to the study treatment after the occurrence of the intercurrent event would have been imputed according to a Hypothetical Strategy in which the intercurrent event would not have occured. Subjects' missing data related to this type of intercurrent event would have been imputed using the Last Observation Carried Forward approach.

This type of intercurrent event **did not occur** in the present and the former studies.

**3.5 Assessments outside the study visit windows**

This type of intercurrent event could occur in the UA-ADRCs group and the corticosteroid group.

In case this type of intercurrent event occured during the present and the former studies, response to study treatment before and after the occurrence of the intercurrent event was handled according to a Hypothetical Strategy in which the intercurrent event would not occur.

This type of intercurrent event did occur in the present and the former studies (Table S15).

**3.6 Discontinuation by investigator because the subject moved out of the country**

This type of intercurrent event could have occured in the UA-ADRCs group and the corticosteroid group, and the intercurrent events could have led to subjects' missing data.

In case this type of intercurrent event would have occured during the present and the former studies, it would have been handled using a combination of the While-on-Treatment Strategy and a Hyopthetical Strategy. Specifically, response to the study treatment before the occurrence of the intercurrent event would have been handled according to the While-on-Treatment Strategy, whereas response to the study treatment after the occurrence of the intercurrent event would have been imputed according to a Hypothetical Strategy in which the intercurrent event would not have occured. Possible subjects' missing data related to this type of intercurrent event would have been imputed using the Last Observation Carried Forward approach.

This type of intercurrent event **did not occur** in the present and the former studies.

**3.6 Discontinuation by investigator for other reasons than the subject having moved out of the country**

This type of intercurrent event could occur in the UA-ADRCs group and the corticosteroid group, and the intercurrent events could lead to subjects' missing data.

In case this type of intercurrent event occured during the present and the former studies, the corresponding strategy for addressing the intercurrent event and adequate imputation of missing data was performed according to the reason that motivated the Investigator to discontinue the subject’s participation in the study (these reasons are addressed in this section).

This type of intercurrent event did occur in the present and the former studies (Table S15).

**3.7 Discontinuation by subject**

This type of intercurrent event could occur in the UA-ADRCs group and the corticosteroid group, and the intercurrent events could lead to missing data.

Subjects' participation in the present and the former studies was voluntary, and the subjects could discontinue participation (refuse all subsequent testing/follow-up) at any time without loss of benefits or penalty.

In case this type of intercurrent event occurred during the present and the former studies, we determined whether withdrawal of consent by the subject was related to any of the types of intercurrent events outlined in this section, and applied the corresponding strategy for addressing the intercurrent event and adequate imputation of missing data. Otherwise this type of intercurrent event was handled using a combination of the While-on-Treatment Strategy and a Hypothetical Strategy. Specifically, response to the study treatment before the occurrence of the intercurrent event was handled according to the While-on-Treatment Strategy, whereas response to the study treatment after the occurrence of the intercurrent event was imputed according to a Hypothetical Strategy in which the intercurrent event did not occur. Subjects' missing data related to this type of intercurrent event were imputed using the Last Observation Carried Forward approach.

This type of intercurrent event did occur in the present and the former studies.

**4. Population-Level Summary**

The Population-Level Summary of the present study (i.e., the variables on which the comparison between treatments were based) is described in detail in the Sections "*Outcome measurements and assessments*", "*Analysis of MRI scans*" and "*Statistical Analysis*" in the *Methods* section of this paper.

**5. Missing data**

Missing data were handled according to the strategies outlined in Section 3 ("*Intercurrent Events*") of this document.
